# The long non-coding RNA GHSROS reprograms prostate cancer cell lines toward a more aggressive phenotype

**DOI:** 10.7717/peerj.10280

**Published:** 2021-02-01

**Authors:** Patrick B. Thomas, Penny Jeffery, Manuel D. Gahete, Eliza Whiteside, Carina Walpole, Michelle Maugham, Lidija Jovanovic, Jennifer Gunter, Elizabeth Williams, Colleen Nelson, Adrian Herington, Raul M. Luque, Rakesh Veedu, Lisa K. Chopin, Inge Seim

**Affiliations:** 1 Ghrelin Research Group, Translational Research Institute, School of Biomedical Sciences, Queensland University of Technology, Brisbane, Queensland, Australia; 2 Comparative and Endocrine Biology Laboratory, Translational Research Institute, School of Biomedical Sciences, Queensland University of Technology, Brisbane, Queensland, Australia; 3 Australian Prostate Cancer Research Centre - Queensland, Queensland University of Technology, Brisbane, Queensland, Australia; 4 Maimonides Institute of Biomedical Research of Cordoba (IMIBIC), Cordoba, Spain; 5 Department of Cell Biology, Physiology and Immunology, University of Cordoba, Cordoba, Spain; 6 Hospital Universitario Reina Sofía (HURS), Cordoba, Spain; 7 Campus de Excelencia Internacional Agroalimentario (ceiA3), Cordoba, Spain; 8 CIBER de la Fisiopatología de la Obesidad y Nutrición (CIBERobn), Cordoba, Spain; 9 Centre for Health Research, University of Southern Queensland, Toowoomba, Queensland, Australia; 10 Institute for Life Sciences and the Environment, University of Southern Queensland, Toowoomba, Queensland, Australia; 11 Centre for Comparative Genomics, Murdoch University, Perth, Western Australia, Australia; 12 Integrative Biology Laboratory, College of Life Sciences, Nanjing Normal University, Nanjing, China

**Keywords:** Long non-coding RNA, lncRNA, Prostate cancer, Antisense transcript, Tumour growth, Gene expression

## Abstract

It is now appreciated that long non-coding RNAs (lncRNAs) are important players in orchestrating cancer progression. In this study we characterized *GHSROS*, a human lncRNA gene on the opposite DNA strand (antisense) to the ghrelin receptor gene, in prostate cancer. The lncRNA was upregulated by prostate tumors from different clinical datasets. Transcriptome data revealed that *GHSROS* alters the expression of cancer-associated genes. Functional analyses in vitro showed that *GHSROS* mediates tumor growth, migration and survival, and resistance to the cytotoxic drug docetaxel. Increased cellular proliferation of *GHSROS*-overexpressing PC3, DU145, and LNCaP prostate cancer cell lines in vitro was recapitulated in a subcutaneous xenograft model. Conversely, in vitro antisense oligonucleotide inhibition of the lncRNA reciprocally regulated cell growth and migration, and gene expression. Notably, *GHSROS* modulates the expression of *PPP2R2C*, the loss of which may drive androgen receptor pathway-independent prostate tumor progression in a subset of prostate cancers. Collectively, our findings suggest that *GHSROS* can reprogram prostate cancer cells toward a more aggressive phenotype and that this lncRNA may represent a potential therapeutic target.

## Introduction

The human genome yields a multitude of RNA transcripts with no obvious protein-coding ability, collectively termed non-coding RNAs (ncRNAs) (Mattick & Rinn, 2015). This concept is currently being challenged however, as some ncRNA give rise to small peptides and proteins (Ruiz-Orera et al., 2014; Choi, Kim & Nam 2019; Wang et al., 2019). A decade of intensive research has revealed that many ncRNAs greater than 200 nucleotides in length have expression patterns and functions as diverse as protein-coding RNAs (Mattick & Rinn, 2015; Huarte, 2015). These long non-coding RNAs (lncRNAs) have emerged as important regulators of gene expression, acting on nearby (*cis*) or distant (*trans*) protein-coding genes (Huarte, 2015). Although the vast majority of lncRNAs remain uncharacterized, it is clear that they play key regulatory roles in development, normal physiology, and disease.

**Figure 1:**
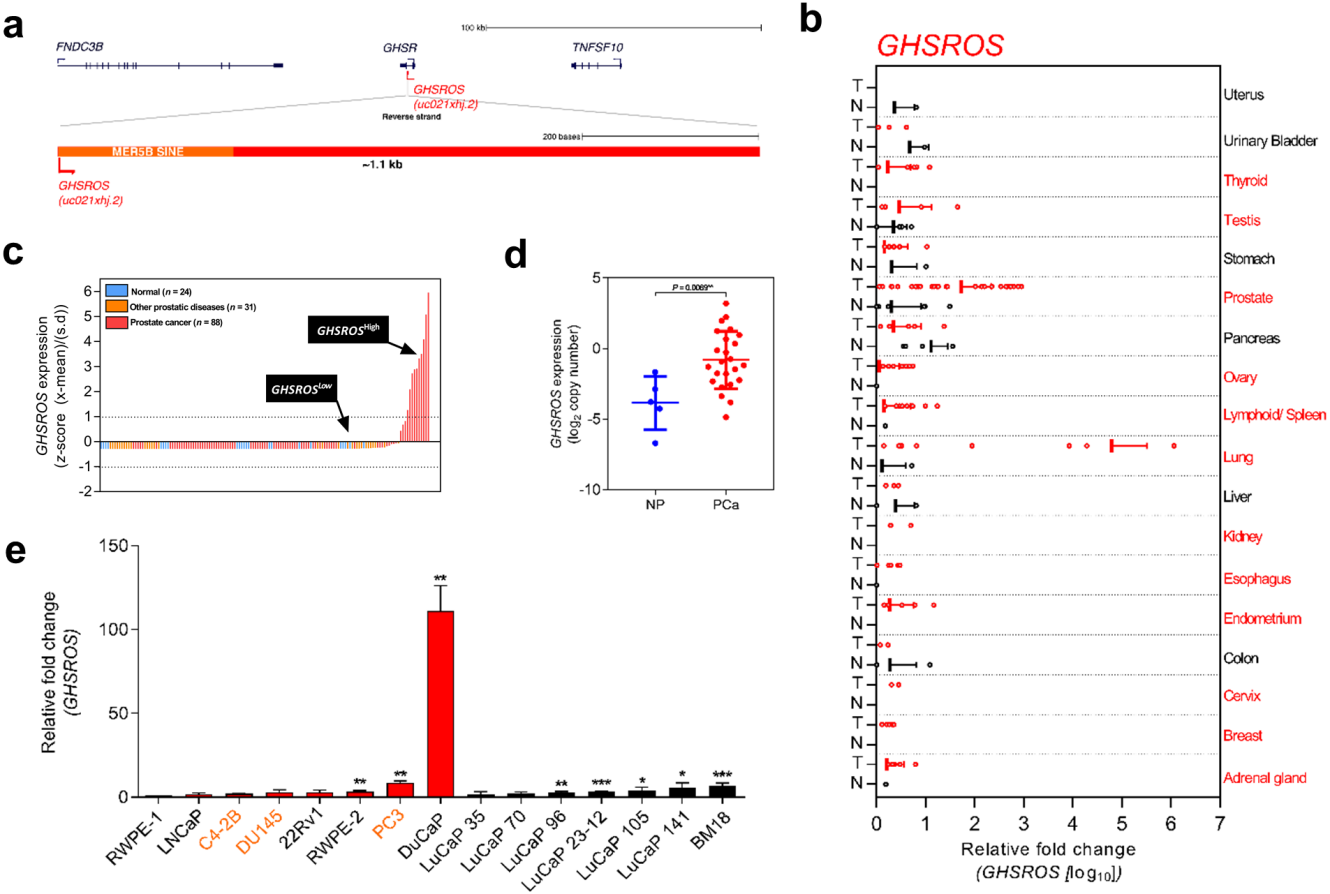
Overview of the lncRNA *GHSROS* and its expression in cancer. (A) The *GHSR* and *GHSROS* gene loci. *GHSR* exons (black), *GHSROS* exon (red), repetitive elements (orange), introns (lines). (B) *GHSROS* expression in 19 cancers (TissueScan Cancer Survey Tissue qPCR panel). N (black) denotes normal tissue; T tumor (red). For each cancer, data are expressed as mean fold change using the comparative 2^−ΔΔ*Ct*^ method against a non-malignant control tissue. Normalized to *β*-actin (*ACTB*). (C) Relative gene expression of *GHSROS* in OriGene cDNA panels of tissues from normal prostate (*n* = 24; blue), primary prostate cancer (*n* = 88; red), and other prostatic diseases (*n* = 31; orange). Determined by qRT-PCR, normalized to ribosomal protein L32 (*RPL32*), and represented as standardized expression values (*Z*-scores). (D) *GHSROS* expression in an Andalusian Biobank prostate tissue cohort. Absolute expression levels were determined by qRT-PCR and adjusted by a normalization factor calculated from the expression levels of three housekeeping genes (*HPRT*, *ACTB*, and *GAPDH*). NP denotes non-malignant prostate. *P ≤ 0.05, Mann–Whitney-Wilcoxon test. (E) Expression of *GHSROS* in immortalized, cultured cell lines and patient-derived xenograft (PDX) lines. Mean ±  s.e.m. (*n* = 3). *P ≤ 0.05, **P ≤ 0.01, *** *P* ≤ 0.001, Student’s *t*-test. Normalized as in (b) to the RWPE-1 non-malignant cell line. Androgen-independent lines are labeled in orange.

We previously identified *GHSROS* (also known as *AS-GHSR*), a 1.1-kb capped and polyadenylated lncRNA gene antisense to the intronic region of the ghrelin receptor gene (*GHSR*) (Whiteside et al., 2013) (Fig. 1A). *GHSROS* harbors a putative human-specific promoter in a transposable element (Whiteside et al., 2013), a pattern frequently found in promoters of lncRNAs which have high tissue specificity and low expression levels (Saxonov, Berg & Brutlag, 2006; Derrien et al., 2012). It is now appreciated that many lncRNAs are equivalent to classical oncogenes or tumor suppressors and drive similar transcriptional programs in diverse cancer types (Huarte, 2015). Indeed, our earlier study showed that *GHSROS* is overexpressed in lung and breast cancer and that its forced overexpression increases migration in derived cancer lines (Whiteside et al., 2013; Thomas et al., 2019). We speculated that *GHSROS* plays a role in other cancers. Prostate cancer is a disease diagnosed in nearly 1.5 million men worldwide annually (Fitzmaurice et al., 2015). Recent studies have revealed that, like breast cancer, prostate cancer is a heterogeneous disease with multiple molecular phenotypes (Tosoian & Antonarakis, 2017; Shoag & Barbieri, 2016; Dagogo-Jack & Shaw, 2018). The identification of genes that drive or mediate these distinct phenotypes is crucial. Although a number of lncRNAs have been reported in prostate cancer, few have been functionally characterized or assessed as therapeutic targets (Mouraviev et al., 2016). Here, we report that *GHSROS* is highly expressed in a subset of prostate tumors. We provide evidence that this lncRNA reprograms prostate cancer cells toward a more aggressive phenotype, possibly by repressing the expression of the tumor suppressor PPP2R2C to allow androgen-independent growth.

## Materials and Methods

### Assessment of *GHSROS* transcription in public high-throughput datasets

To expand on Northern blot and qRT-PCR analyses, which suggest that the lncRNA *GHSROS* is expressed at low levels (Whiteside et al., 2013), we interrogated ∼4,000 oligonucleotide microarrays with probes for known and predicted exons (Affymetrix GeneChip Exon 1.0 ST). An RNA-sequencing dataset averaging ∼160M reads from metastatic castration-resistant prostate cancer was also examined. See Supplementary information and Table S10.

### Cell culture, prostate cancer patient derived xenograft (PDX) models, and treatments

The PC3 (ATCC CRL-1435), DU145 (ATCC HTB-81), LNCaP (ATCC CRL-1740), and 22Rv1 (ATCC CRL-2505) prostate cancer cell lines, the ES-2 ovarian cancer cell line (ATCC CRL-1978), and the A549 lung cancer cell line (ATCC CCL-185), were obtained from the American Type Culture Collection (ATCC, Rockville, MD, USA) and the DuCaP (Lee et al., 2001) cell line from. The C4-2B (Thalmann et al., 1994) prostate cancer cell line, six LuCaP prostate-derived xenograft (PDX) lines (Nguyen et al., 2017), and the BM18 PDX cell line (McCulloch et al., 2005) were available in our laboratory. All prostate cancer and the ovarian cancer cell line were maintained in Roswell Park Memorial Institute (RPMI) 1640 medium (RPMI-1640; Invitrogen, Carlsbad, CA) with 10% Fetal Calf Serum (FCS, Thermo Fisher Scientific Australia, Scoresby, VIC, Australia), supplemented with 100 U/mL penicillin G and 100 ng/mL streptomycin (Invitrogen). The A549 cell line was maintained in Dulbecco’s Modified Eagle Medium: Nutrient Mixture F-12 (DMEM/F12) medium (Invitrogen) with 10% FCS (Thermo Fisher Scientific Australia) supplemented with 100 U/mL penicillin G and 100 ng/mL streptomycin (Invitrogen). The non-tumorigenic RWPE-1 (ATCC CRL-11609) and the transformed, tumorigenic RWPE-2 (ATCC CRL-11610) prostate epithelium-derived cell lines were cultured in keratinocyte serum-free medium (Invitrogen) supplemented with 50 µg/mL bovine pituitary extract and five ng/mL epidermal growth factor (Invitrogen). All cell lines were passaged at 2- to 3-day intervals on reaching 70% confluency using TrypLE Select (Invitrogen). Cell morphology and viability were monitored by microscopic observation and regular Mycoplasma testing was performed (Universal Mycoplasma Detection Kit, ATCC). Cells were treated with 10 µM enzalutamide (ENZ; Selleck Chemicals, Houston, TX, USA) or 1–20 nM docetaxel (DTX; Sigma Aldrich, St. Louis, MO, USA) for 96 h (functional assays) or 48 h (qRT-PCR) and compared to dimethyl sulfoxide (DMSO) (Sigma Aldrich, St. Louis, MO, USA) vehicle control.

### *GHSROS* qRT-PCR interrogation of human tissue specimens

To investigate the expression of *GHSROS* in cancer, we initially interrogated a TissueScan Cancer Survey Tissue qPCR panel (CSRT102; OriGene, Rockville, MD, USA); cDNA arrayed on multi-well PCR plates. Expression was compared between tumour and normal tissue. For each cancer type, data were expressed as mean fold change using the comparative 2^−ΔΔ*Ct*^ method against a non-malignant control tissue and normalized to *β*-actin (*ACTB*).

To further investigate the expression of *GHSROS* in prostate cancer TissueScan Prostate Cancer Tissue qPCR panels (HPRT101, HPRT102, and HPRT103) were obtained from OriGene. The cDNA panels contained of a total of 24 normal prostate-derived samples, 31 abnormal prostate samples (defined as lesions), and 88 prostate tumor samples. These panels were examined by qRT-PCR, as described above, except that the housekeeping gene ribosomal protein L32 (*RPL32*) was employed.

An independent cohort was obtained from the Andalusian Biobank (Servicio Andaluz de Salud, Spain). It consisted of tissue from 28 patients with clinical high-grade prostate cancer (10 localized and 18 metastatic tumors) and 8 normal prostate tissue samples. RT-PCR was performed using Brilliant III SYBR Green Master Mix and a Stratagene Mx3000p instrument (both from Agilent, La Jolla, CA, USA), as previously described (Hormaechea-Agulla et al., 2016). Briefly, samples on the same plate were analysed with a standard curve to estimate mRNA copy number (tenfold dilutions of synthetic cDNA template for each transcript). No-RNA controls were carried out for all primer pairs. To control for variations in the amount of RNA used, and the efficiency of the reverse-transcription reaction, the expression level (copy number) of each transcript was adjusted by a normalization factor (NF) obtained from the expression of three housekeeping genes (*ACTB*, *HPRT*, and *GAPDH*) using the geNorm algorithm (Vandesompele et al., 2002). Primers used are listed in Table S11.

### Production of *GHSROS* overexpressing cancer cell lines

Full-length *GHSROS* transcript was cloned into the *pTargeT* mammalian expression vector (Promega, Madison, WI). PC3, DU145, and A549 cell lines were transfected with *GHSROS-pTargeT* DNA, or vector alone (empty vector), (using Lipofectamine LTX, Invitrogen) according to the manufacturer’s instructions. Cells were incubated for 24 h in LTX and selected with geneticin (100–1,500 µg/mL G418, Invitrogen). As LNCaP prostate cancer cells were difficult to transfect using lipid-mediated transfection, we employed lentiviral transduction. Briefly, *pReceiver-Lv105* vectors, expressing full length *GHSROS,* or empty control vectors, were obtained from GeneCopoeia (Rockville, MD). For stable overexpression, LNCaP cells were seeded at 50–60% confluency and transduced with *GHSROS,* or empty vector control lentiviral constructs in the presence of 8 µg/ml polybrene (Sigma Aldrich). Following a 48-hour incubation period, transduced cells were selected with 1 µg/mL puromycin (Invitrogen). *GHSROS* expression was confirmed approximately 3 weeks after selection by qRT-PCR, every 2–3 weeks, and before every functional experiment (see Fig. S5).

### RNA extraction, reverse transcription and quantitative reverse transcription Polymerase Chain Reaction (qRT-PCR)

Total RNA was extracted from cell pellets using an RNeasy Plus Mini Kit (QIAGEN, Hilden, Germany) with a genomic DNA (gDNA) Eliminator spin column. To remove contaminating genomic DNA, 1 µg RNA was DNase treated prior to cDNA synthesis with Superscript III (Invitrogen). qRT-PCR was performed using the AB7500 FAST sequence detection thermal cycler (Applied Biosystems, Foster City, CA), or the ViiA Real-Time PCR system (Applied Biosystems) with SYBR Green PCR Master Mix (QIAGEN) using primers listed in Table S11. A negative control (water instead of template) was used in each real-time plate for each primer set. All real-time experiments were performed in triplicate. Baseline and threshold values (C_t_) were obtained using ABI 7500 Prism and the relative expression of mRNA was calculated using the comparative 2^−ΔΔ*Ct*^ method (Livak & Schmittgen, 2001). Expression was normalized to the housekeeping gene ribosomal protein L32 (*RPL32*). Statistical analyses were performed using GraphPad Prism v.6.01 software (GraphPad Software, Inc., San Diego, CA). Student’s *t*-test or Mann–Whitney-Wilcoxon tests were used to assess the statistical significance of all the direct comparisons.

### Cell proliferation assays

Proliferation assays were performed using an xCELLigence real-time cell analyzer (RTCA) DP instrument (ACEA Biosciences, San Diego, CA). This system employs sensor impedance technology to quantify the status of the cell using a unit-less parameter termed the cell index (CI). The CI represents the status of the cell based on the measured relative changes in electrical impedance that occur in the presence and absence of cells in the wells (generated by the software, according to the formula CI = (Z_i_–Z_0_)/15 Ω, where Z _i_ is the impedance at an individual point of time during the experiment and Z_0_ the impedance at the start of the experiment). Impedance is measured at three different frequencies (10, 25 or 50 kHz). Briefly, 5 × 10^3^ cells were trypsinized and seeded into a 96 well plate (E-plate) and grown for 48 h in 150 µl growth media. Cell index was measured every 15 min and all experiments were performed in triplicate, with at least three independent repeats. Because cells did not attach well to the gold microelectrodes of the xCELLigence instrument, LNCaP proliferation was quantified by measuring the cleavage of WST-1 (Roche, Basel, Switzerland). Briefly, 5 × 10^4^ cells/ well were seeded in 96-well plates (BD Biosciences, Franklin Lakes, NJ, USA) and propagated for 72 h in complete medium. To determine cell number, absorbance was measured using the FLUOstar Omega spectrophotometer (BMG, Ortenberg, Germany) at 440 nm using a reference wavelength of 600 nm. All proliferation experiments were performed independently three times, with 8 replicates each.

### Cell viability assay

LNCaP and PC3 vector or *GHSROS* over-expressing cells (5,000 cells/well) were seeded in 96-well plates (BD Biosciences) and propagated overnight in complete medium. LNCaP cells were treated with standard doses of test compounds in both charcoal stripped FCS (CSS) or 2% FCS. PC3 cells were treated with increasing doses of docetaxel in 2% FCS. After a 96-hour period cell viability was measured using a WST-1 cell proliferation assay (Roche, Nonnenwald, Penzberg, Germany) according to the manufacturer’s instructions. All viability experiments were performed independently three times, with 4 replicates each.

### Cell migration assays

Migration assays were performed using an xCELLigence RTCA DP instrument (ACEA Biosciences). Briefly, 5 × 10^4^ cells/well were seeded on the top chamber in 150 µl serum-free media. The lower chamber contained 160 µl media with 10% FCS as a chemo-attractant. Cell index was measured every 15 min for 24 h to indicate the rate of cell migration to the lower chamber. All experiments were performed in triplicate with at least 3 independent repeats. Because cells did not attach well to the gold microelectrodes of the xCELLigence instrument, LNCaPs migration was assessed using a transwell assay. Briefly, 6 × 10^5^ cells were suspended in serum-free medium and added to the upper chamber of inserts coated with a polycarbonate membrane (8 µm pore size; BD Biosciences). Cells in 12-well plates were allowed to migrate for 24 h in response to a chemoattractant (10% FBS) in the lower chamber. After 24 h, cells remaining in the upper chamber were removed. Cells that had migrated to the lower surface of the membrane were fixed with methanol (100%) and stained with 1% crystal violet. Acetic acid (10%, v/v) was used to extract the crystal violet and absorbance was measured at 595 nm. Each experiment consisted of three replicates and was repeated independently three times.

### Locked nucleic acid-antisense oligonucleotides (LNA-ASO)

Two distinct Locked nucleic acid (LNA) ASOs, RNV104L and RNV124, complementary to different regions of *GHSROS* (see Fig. S6), were designed in-house and synthesized commercially (Exiqon, Vedbæk, Denmark). The ASOs contained two consecutive LNA nucleotides at the 5′-end and three consecutive LNA nucleotides at the 3′-end –in line with gapmer design principles. The LNA ASO sequences were as follows: scrambled control sequence: 5′-GC TTCGACTCGTAATCACCTA-3′; RNV124 (underlined bases denote LNA nucleotides): 5′-ATAA ACCTGCTAGTGTCCTCC-3′; RNV104L: 5′-GTTAACTTTCTTCTTCCTTG-3′. Lyophilized oligonucleotides were resuspended in ultrapure H_2_O (Invitrogen) and stored as a 100 µM stock solution at −20 °C. Briefly, LNA-ASOs were diluted to 20 µM in OptiMEM I Reduced Serum Medium (Invitrogen) and cultured cells were transfected according to the manufacturer’s instructions. Cultured cells were incubated at 37 °C in 5% CO_2_ for 4 h, before 500 µl growth medium, containing 30% FCS, was added to the serum-free medium. The cells were transfected for 24–72 h and *GHSROS* levels assessed by qRT-PCR.

### Mouse subcutaneous in vivo xenograft models

All mouse studies were carried out with approval from the University of Queensland and the Queensland University of Technology Animal Ethics Committees. PC3-GHSROS, PC3-vector, DU145-GHSROS, DU145-vector, LNCaP-GHSROS, and LNCaP-vector cell lines were injected subcutaneously into the flank of 4–5-week-old male NSG mice (Shultz et al., 2005) (obtained from Animal Resource Centre, Murdoch, WA, Australia). Cells were injected in a 1:1 ratio with growth factor-reduced Matrigel (Thermo Fisher) (*n* = 8–10 per cell line) and tumors measured twice weekly with digital calipers (ProSciTech, Kirwan, QLD, Australia). Neither randomization nor blinding for animal use was performed because we commercially obtained these mice with the same genetic background. Animals were euthanized once tumor volume reached 1,000 mm, or at other ethical endpoints. At the experimental endpoint, the primary tumor was resected, divided in half, snap frozen and stored at −80 °C.

### Histology and immunohistochemistry

For histological analysis, cryosections (6–10 µm thick) were prepared using a Leica CM1850 cryotome (Wetzlar, Germany). Sections were collected onto warm, charged Menzel Superfrost slides (Thermo Fisher), fixed in ice-cold 100% acetone, air dried and stored at −80 °C. For immunohistochemistry, tissues were fixed in paraformaldehyde and dehydrated through a graded series of ethanol and xylene, before being embedded in paraffin. Sections (5 µm) were mounted on to glass Menzel Superfrost slides (ThermoFisher Scientific). Immunohistochemistry was performed using antibodies for the proliferation marker Ki67 (rabbit anti-human Ki67, Abcam, Cambridge, UK) and for the infiltration of murine blood vessels using rabbit anti-murine CD31 antibody (Abcam). Tissue sections were incubated with HRP-polymer conjugates (SuperPicture, Thermo Fisher Scientific), and incubated with the chromagen diaminobenzidine (DAB) (Dako, Glostrup, Denmark), as per manufacturer’s specifications. Slides were counterstained with Mayer’s hematoxylin, dehydrated, and mounted with coverslips using D.P.X neutral mounting medium (Sigma Aldrich). All sections were counterstained with Mayer’s hematoxylin (Sigma Aldrich) and mounted with coverslips using D.P.X with Colourfast (Fronine, ThermoFisher Scientific).

### RNA-sequencing of *GHSROS* overexpressing PC3 and LNCaP cells

Total RNA was extracted from in vitro cultured PC3-GHSROS cells and controls, as outlined above. RNA purity was analysed using an Agilent 2100 Bioanalyzer, and RNA with an RNA Integrity Number (RIN) above 7 used for RNA-sequencing (RNA-seq). Strand-specific RNA-seq was performed by Macrogen, South Korea. A TruSeq stranded mRNA library (Illumina) was constructed and RNA sequencing performed (50 million reads) on a HiSeq 2000 instrument (Illumina) with 100 bp paired end reads. For the LNCaP-GHSROS xenograft tumors and controls (empty vector control lentiviral constructs), total RNA and RNA purity was extracted analysed as above. Strand-specific RNA-seq was performed by the South Australian Health and Medical Research Institute (SAHMRI, Adelaide, SA, Australia). A TruSeq stranded mRNA library (Illumina) was constructed and RNA sequencing performed (35 million reads) on a Nextseq 500 instrument (Illumina) with 75bp single end reads. See Supplementary information for details regarding RNA-seq analysis. Raw and processed RNA-sequencing (transcriptome) data have been deposited in Gene Expression Omnibus (GEO) with the accession codes GSE86097 (*GHSROS* overexpression in cultured PC3 cells) and GSE103320 (*GHSROS* overexpression in LNCaP xenografts).

### Gene Ontology (GO) term analyses and Oncomine Concept analysis

Gene Ontology (GO) term analyses were performed using DAVID (Database for Annotation, Visualization and Integrated Discovery) (Da Huang, Sherman & Lempicki, 2009). Briefly, to test for enrichment we interrogated DAVID’s GO FAT database with genes differentially expressed in PC3-*GHSROS* cells. The DAVID functional annotation tool categorizes GO terms and calculates an ‘enrichment score’ or EASE score (a modified Fisher’s exact test-derived *P*-value). Categories with smaller *P*-values (*P* ≤ 0.01) and larger fold-enrichments (≥2.0) were considered interesting and most likely to convey biological meaning.

To perform Oncomine meta-analysis, genes differentially expressed in PC3-GHSROS were separated into ‘over-expressed’ and ‘under-expressed’ gene sets. The Oncomine database (Hodes et al., 2007) was interrogated by importing these genes, and enriched concepts were generated and ordered by *P*-values (calculated using Fisher’s exact test). Only datasets with an odds ratio ≥ 3.0 and a *P-* value ≤ 0.01 were retained. The datasets were exported as nodes and edges for network visualization in Cytoscape (Shannon et al., 2003) (v3.4.0). The network layout and node position were generated using the Force-Directed Layout algorithm (Suderman & Hallett, 2007), with odds ratio as the leading parameter for the edge weight. Using our custom concept generated lists, we next sought to assess the differential expression of our gene lists in two prostate cancer microarray datasets: Grasso (Grasso et al., 2012) (59 localized and 35 metastatic prostate tumors) and Taylor (Taylor et al., 2010) (123 localized and 27 metastatic prostate tumors). Differentially expressed genes were ranked and results exported as fold change (log_2_ transformed, median centered). Data was filtered for significance with *P*-value set at ≤0.05 and Benjamini–Hochberg false discovery rate (FDR) *Q*-value (Benjamini & Hochberg, 1995) at ≤ 0.25; a threshold deemed suitable to find biologically relevant transcriptional signatures (Luck et al., 2014; Simola et al., 2013).

### LP50 prostate cancer cell line *AR* knockdown microarray

Publicly available Affymetrix HG-U133 Plus 2.0 microarray data (NCBI GEO accession no. GSE22483) from a substrain of the LNCaP cell line: the androgen-independent late passage LNCaP cells (LP50) was interrogated. This cell line was subjected to androgen receptor (*AR*) knockdown by shRNA (Gonit et al., 2011). The array (*n* = 2, of AR shRNA and scrambled control) was normalized to housekeeping genes using the Affymetrix Gene Chip Operating System v1.4 (Gonit et al., 2011). Prior to differential expression analysis, the probe set was pre-filtered, using the R statistical programming language, as follows: probes with mean expression values in the lowest 20^th^ percentile of the array was removed. Differential expression was determined by the R package ‘limma’ (Ritchie et al., 2015) and probes with a Benjamini–Hochberg adjusted *P*-value (*Q*; BH-FDR) ≤ 0.05 considered significant. Gene annotations were obtained using the R/Bioconductor packages ‘Biobase’ (Robinson & Oshlack, 2010) and ‘GEOquery’ (Davis & Meltzer, 2007).

### Survival analysis in clinical gene expression datasets

Two datasets were interrogated: Taylor (Taylor et al., 2010) and TCGA-PRAD from The Cancer Genomics Atlas (TCGA) consortium, which contains tumors from patients with moderate- (∼39% Gleason score 6 and 3 + 4) and high- (∼61% Gleason 4+3 and Gleason score 8-10) risk localized prostate carcinoma (Cancer Genome Atlas Research Network, 2015). Briefly, in the case of TCGA-PRAD, the UCSC Xena Browser (Casper et al., 2015) was used to obtain normalized gene expression values, represented as log_2_ (normalized counts+1), from the ‘TCGA TARGET GTeX’ dataset consisting of ∼12,000 tissue samples from 31 cancers (Vivian et al., 2017). To obtain up-to-date overall survival (OS) and disease-free survival (DFS) information, we manually queried cBioPortal for Cancer Genomics (Cerami et al., 2012; Gao et al., 2013) (last accessed 05.08.16). See Supplementary information for details.

### Statistical analyses

Data values were expressed as mean ± s.e.m. of at least two independent experiments and evaluated using Student’s *t*-test for unpaired samples, or otherwise specified. Mean differences were considered significant when *P* ≤ 0.05. *Q*-values denote multiple testing correction (Benjamini–Hochberg) adjusted *P*-values (Benjamini & Hochberg, 1995). Normalized high-throughput gene expression data were analyzed using LIMMA, employing a modified version of the Student’s *t*-test (moderated *t*-test) where the standard errors are reduced toward a common value using an empirical Bayesian model robust for datasets with few biological replicates (Ritchie et al., 2015). Statistical analyses were performed using GraphPad Prism v.6.01 software (GraphPad Software, Inc., San Diego, CA), or the R statistical programming language.

## Results

### *GHSROS* is expressed in prostate cancer

Microarrays and RNA-sequencing are commonly used to assess the expression of genes. LncRNAs are often expressed at orders of magnitude lower than protein-coding transcripts, however, making them difficult to detect (Derrien et al., 2012; Ruiz-Orera et al., 2014; Kutter et al., 2012; Cabili et al., 2011; Necsulea et al., 2014; Wang et al., 2011). Interrogation of exon arrays harboring four different strand-specific probes against *GHSROS* demonstrated that the lncRNA is actively transcribed, although expressed at low levels in cancer cell lines and tissues (Fig. S1), consistent with previous observations from Northern blotting and RT-PCR experiments (Whiteside et al., 2013). The low expression across the *GHSROS* and *GHSR* loci in RNA-seq datasets is illustrated in Fig.  S2. Collectively, these data demonstrate that it is currently not possible to detect *GHSROS* in public genome-wide gene expression datasets.

We next evaluated *GHSROS* expression in a qRT-PCR tissue array of 18 cancers. This analysis revealed particularly high *GHSROS* expression in lung tumors, as previously reported (Whiteside et al., 2013), and elevated expression in prostate tumors (Fig. 1B). Analysis of additional prostate tissue-derived cDNA arrays revealed that *GHSROS* could be detected in approximately 41.7% of all normal prostate tissues (*n* = 24), 55.7% of tumors (*n* = 88), and 58.1% of other prostatic diseases (e.g., prostatitis; *n* = 31) (Table S1). *GHSROS* was highly expressed by a subset of prostate tumors (∼11.4%; *Z*-score >  1) (Fig. 1C) and elevated in tumors with Gleason scores 8–10 (Fig. S3; Table S1; Mann–Whitney-Wilcoxon test *P* = 0.0021). To expand on these observations, we examined an independent cohort of eight normal prostate tissue specimens and 28 primary tumors with high Gleason scores (18 of which had metastases at biopsy). Similarly, *GHSROS* expression was significantly elevated in tumors compared to normal prostate tissue (Mann–Whitney-Wilcoxon test, *P* = 0.0070) (Fig. 1D; Fig. S4; Table S2).

As the functional thresholds of long non-coding RNAs are difficult to gauge and likely to be cell specific (Geisler & Coller, 2013), we identified cell lines with a range of endogenous *GHSROS* expression. Compared to the RWPE-1 benign prostate-derived cell line, higher expression was observed in the PC3 (*P* = 0.00040, Student’s *t-* test) (Fig. 1E) and DuCaP prostate cancer cell lines (*P* = 0.0024), and expression was similar to RWPE-1 in the DU145 (*P* = 0.29) and LNCaP prostate cancer cell lines (*P* = 0.49). We also assessed the expression of *GHSROS* in patient-derived xenografts (PDXs). Compared to the RWPE-1 cell line, *GHSROS* was significantly upregulated (*P* ≤** 0.05) in 4/6 of the LuCaP series of PDX lines (Nguyen et al., 2017) and in the BM18 femoral metastasis-derived androgen-responsive PDX line (McCulloch et al., 2005) (*P* = 0.0005) (Fig. 1E).

**Figure 2:**
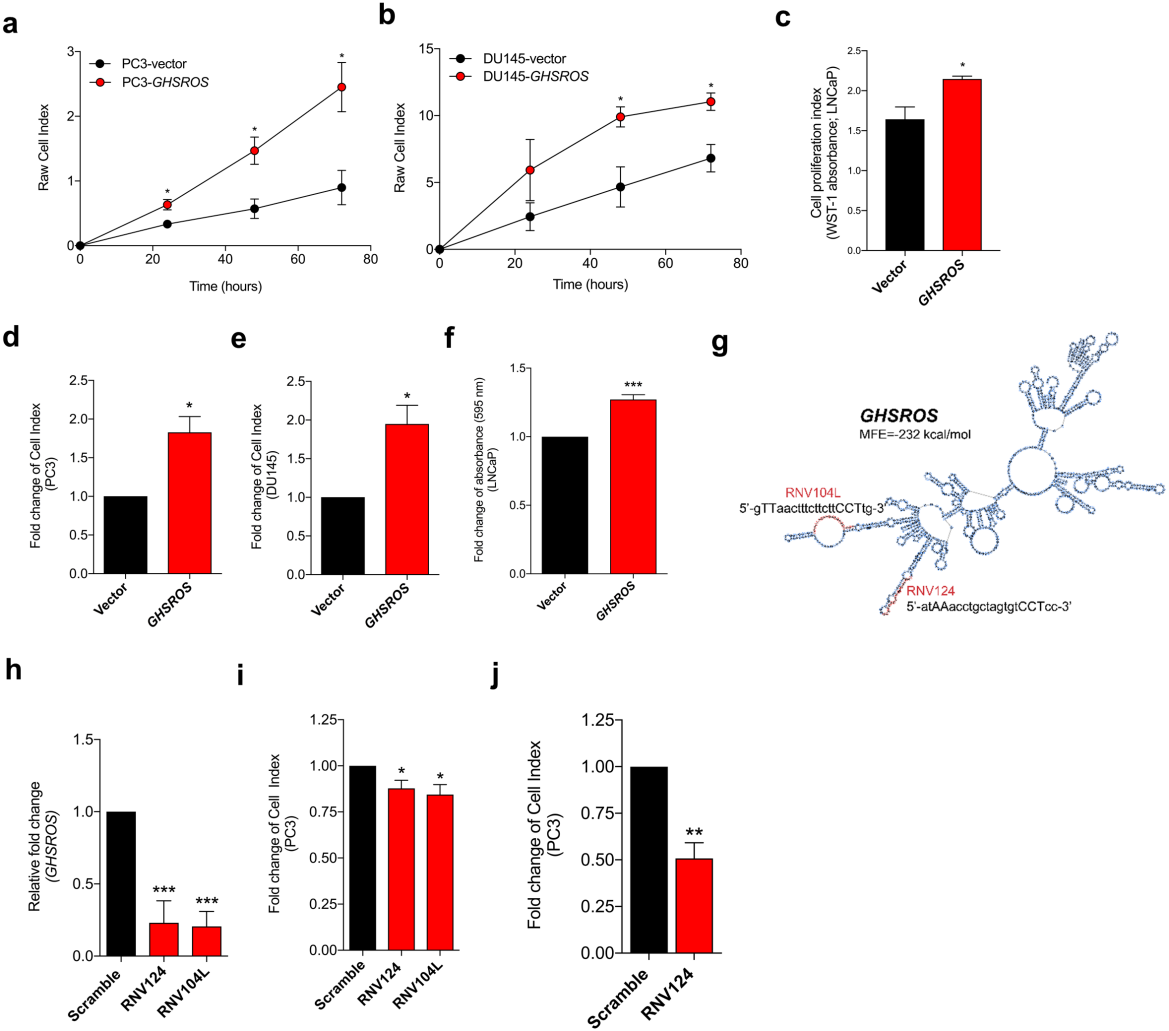
*GHSROS* promotes human prostate cancer cell line growth and motility in vitro. (A, B, C) Increased proliferation in *GHSROS*-overexpressing cells. PC3 and DU145 cells were assessed using an xCELLigence real-time cell analyzer for 72 h; LNCaP using a WST-1 assay at 72 h. Vector denotes empty control plasmid. Mean ±  s.e.m. (*n* = 3). *P ≤ 0.05, **P ≤ 0.01, ***P ≤ 0.001, Student’s *t*-test. (D, E, F) Increased migration in *GHSROS*-overexpressing cells. PC3 and DU145 cells were assessed using an xCELLigence real-time cell analyzer for 24 h; LNCaP using a transwell assay (at 24 h; *n* = 3). Parameters and annotations as in (A). (G) Prediction of *GHSROS* RNA secondary structure. The location of locked nucleic antisense oligonucleotides (LNA ASOs) that target the lncRNA are shown in red. MFE denotes minimum free energy. (H) LNA ASOs reduced *GHSROS* expression in PC3 cells (measured 48 h post-transfection). Fold-enrichment of *GHSROS* normalized to *RPL32* and compared to scrambled control (*n* = 3). Parameters and annotations as in (A). (i) *GHSROS* knockdown reduces PC3 proliferation (*n* = 3). Parameters and annotations as in (A). *GHSROS* knockdown reduces PC3 migration. (J) RNV124 reduced cell migration at 18 h (*n* = 3). Parameters and annotations as in (C).

### *GHSROS* promotes growth and motility of prostate cancer cells in vitro

To gain insights into *GHSROS*, we assessed its function in three prostate-derived cell lines by stably overexpressing the lncRNA in PC3, DU145, and LNCaP cells (denoted PC3-GHSROS, DU145-GHSROS, and LNCaP-GHSROS) (Fig. S5). Cell proliferation over 72 h (measured by a xCELLigence real-time cell analysis instrument) was increased in PC3 (*P* = 0.029, Student’s *t*-test) and DU145 (*P* = 0.026) *GHSROS*-overexpressing cells (Figs. 2A, 2B). LNCaP cells did not attach well to the gold electrodes of the xCELLigence instrument (data not shown), and we therefore utilized a WST-1 assay to assess this cell line. Similar to PC3 and DU145 cells overexpressing *GHSROS*, proliferation was also increased in LNCaP-GHSROS cells at 72 h (*P* = 0.040) (Fig. 2C). *GHSROS* overexpression also increased the rate of cell migration in PC3 (*P* = 0.0064, Student’s *t*-test), DU145 (*P* = 0.017), and LNCaP cell lines (*P* = 0.00020) over 24 h (Figs. 2D, 2E, 2F) (where LNCaP was assessed by a standard transwell migration assay; PC3 and DU145 using the xCELLigence instrument). To confirm the in vitro functional effects of *GHSROS*, we designed locked nucleic antisense oligonucleotides (LNA-ASOs) to strand-specifically silence endogenous *GHSROS* expression (Fig. 2G; Fig. S6). Two LNA-ASOs targeting distinct regions of *GHSROS*, RNV124 and RNV104L, independently reduced the expression of *GHSROS* (percentage knockdown of ∼63% and ∼71%, respectively) in native PC3 cells 48 h post transfection compared to scrambled control (*P* = 0.0002 and *P* = 0.0001, Student’s *t*-test) (Fig. 2H). Moreover, *GHSROS* knockdown attenuated cell proliferation (RNV124, *P* = 0.049; RNV104L, *P* = 0.030) (Fig. 2I) and migration in the PC3 cell line over 18 h (RNV124, *P* = 0.0042) (Fig. 2J)—the reciprocal effects observed when *GHSROS* was forcibly overexpressed.

### *GHSROS* is associated with cell survival and resistance to the cytotoxic drug docetaxel

**Figure 3:**
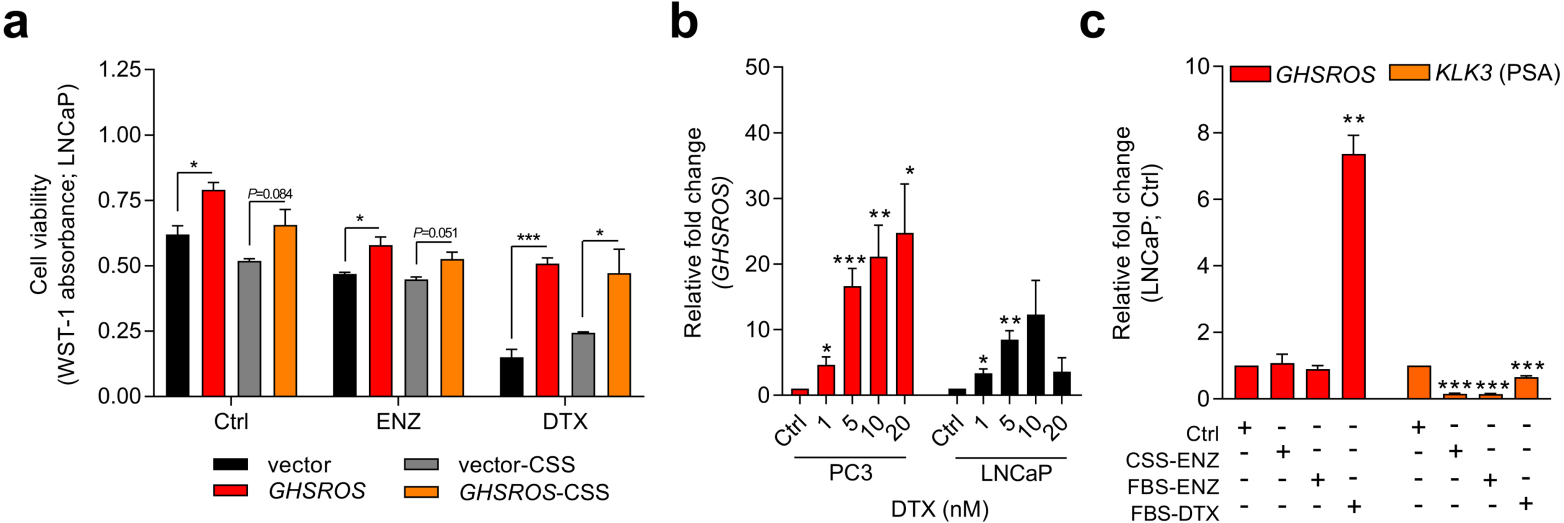
*GHSROS* mediates cell survival and resistance to the cytotoxic drug docetaxel. (A) Viability of *GHSROS*-overexpressing LNCaP cells under different culture conditions. Cell number was assessed using WST-1. Cells were treated with enzalutamide (ENZ; 10 µM) or docetaxel (DTX; 5 nM) for 96 h and grown in either 2% FBS or 5% charcoal stripped serum (CSS) RPMI-1640 media (*n* = 3). Mean ±   s.e.m. *P ≤ 0.05, ***P ≤ 0.001, Student’s *t*-test. (B) *GHSROS* expression of native PC3 and LNCaP cells treated with docetaxel. Cells were grown in RPMI-1640 media with 2% FBS and treated with 1–20 nM docetaxel (DTX) for 48 h (*n* = 3). Fold-enrichment of *GHSROS* normalized to *RPL32* and compared to empty vector control. Parameters and annotations as in (A). (C) *GHSROS* and PSA (*KLK3*) expression of native LNCaP cells treated with ENZ (10 µM in 2% FBS or 5% CSS RPMI-1640) or DTX (5 nM in 2% FBS RPMI-1640) for 48 h (*n* = 3). Parameters and annotations as in (A).

Knockdown experiments also revealed that *GHSROS* protected PC3 prostate cancer cells from death by serum starvation (Fig. S7). This observation led us to examine whether *GHSROS* contributes to cell survival following chemotherapy. The current treatment of choice for advanced, castration-resistant prostate cancer (CRPC; the fatal final stage of the disease) after the failure of hormonal therapy is the cytotoxic drug docetaxel, a semi-synthetic taxoid that induces cell cycle arrest. At the half maximal inhibitory concentration (IC_50_) of docetaxel (5 nM for LNCaP (Gan et al., 2016)), survival was significantly increased in *GHSROS*-overexpressing LNCaP cells (*P* ≤ 0.05, Student’s *t*-test) (Fig. 3A) after 96 h. A similar, less pronounced response was observed in LNCaP cells treated with enzalutamide, a hormonal therapy used to target the androgen receptor in metastatic, castration-resistant tumors (Drake, Sharma & Gerritsen, 2014) (Fig. 3A).

Survival pathways are induced after docetaxel treatment in prostate cancer (Sonpavde et al., 2015; Chandrasekar et al., 2015), and resistance may develop after chemotherapy (acquired resistance) or exist in treatment-naïve patients (innate resistance) (Sonpavde et al., 2015). The pronounced survival following docetaxel treatment in *GHSROS*-overexpressing LNCaP cells led us to speculate that endogenous *GHSROS* expression also contributed to drug resistance. Docetaxel significantly increased *GHSROS* expression in native LNCaP and PC3 cells—in a dose-dependent manner and at concentrations both above and below their respective IC_50_ values (Fig. 3B). The lncRNA was not differentially expressed in charcoal stripped serum (CSS), used to simulate androgen deprivation therapy, or following treatment with enzalutamide (Fig. 3C). In agreement with previous reports (Lee et al., 2014; Tran et al., 2009), the gene coding for prostate specific antigen (PSA; *KLK3*) was downregulated by docetaxel and enzalutamide in LNCaP cells (-6.6-fold, *P* = 0.00070, Student’s *t*-test) (Fig. 3C). Taken together, these data suggest that *GHSROS* mediates tumor survival and resistance to the cytotoxic chemotherapy docetaxel.

### *GHSROS* potentiates tumor growth in vivo

**Figure 4:**
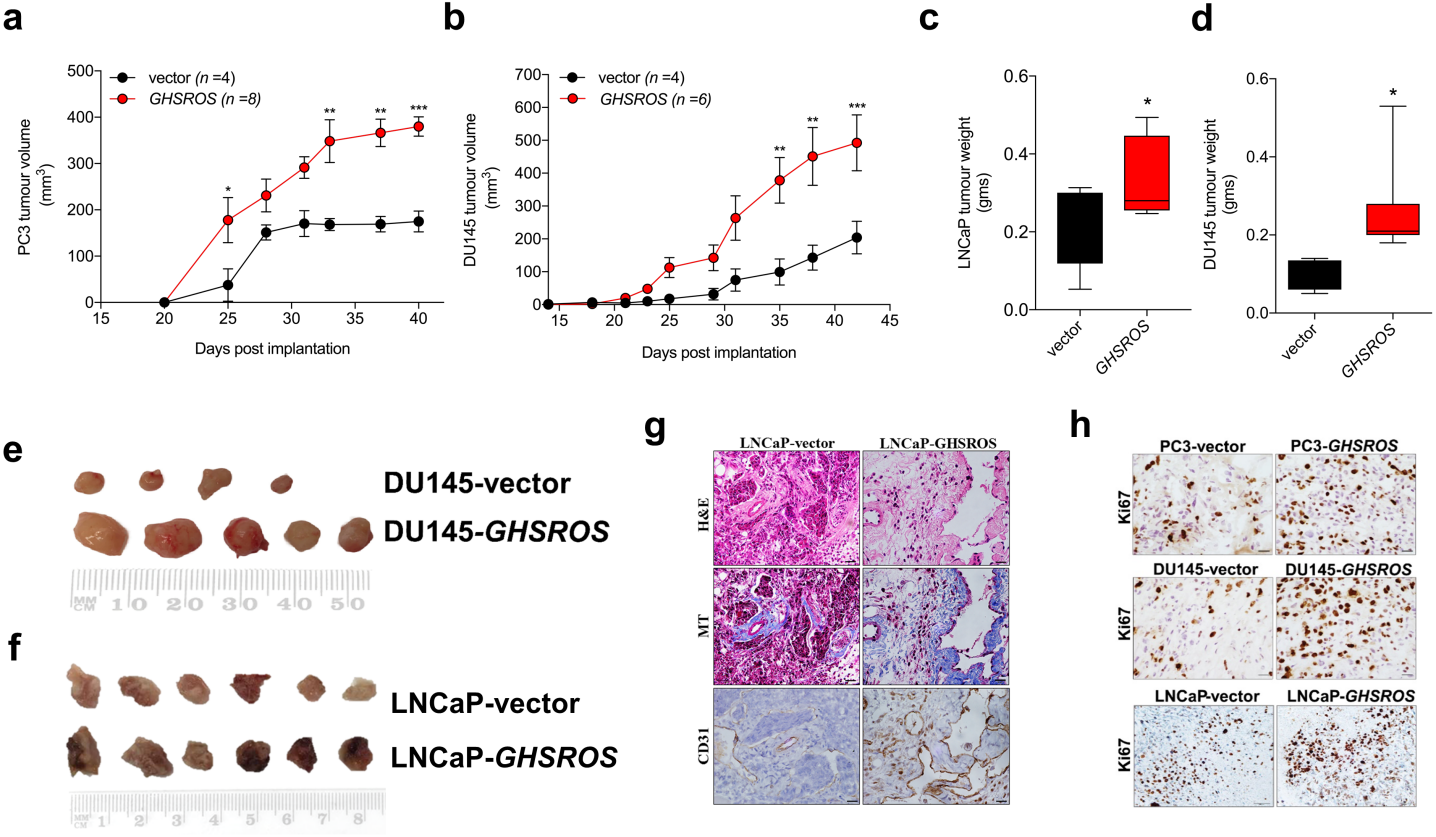
*GHSROS* promotes human prostate cancer cell line growth in vivo. (A) Time course for PC3-GHSROS (*n* = 8) and vector control (*n* = 4) xenograft tumor volumes. (B) DU145-GHSROS (*n* = 6) and vector control (*n* = 4). Mean ±  s.e.m. *P ≤ 0.05, **P ≤ 0.01, ***P ≤ 0.001, two-way ANOVA with Bonferonni’s *post hoc* analysis. Tumors were measured with digital calipers. (C) Tumor weights of LNCaP ( *GHSROS*-overexpressing *n* = 9, vector *n* = 8) or (D) DU145. *P ≤ 0.05, Mann–Whitney-Wilcoxon test. (E) Size comparisons of DU145 (top panel) and (F) LNCaP (bottom panel) xenografts overexpressing *GHSROS* or empty vector. (G) Representative morphology of LNCaP xenografts overexpressing *GHSROS* or empty vector. Tissue was stained with hematoxylin and eosin (H&E), Masson’s Trichrome (MT; collagen; blue) and CD31 (endothelial marker; brown immunoreactivity). Scale bar = 20 µm. (H) Representative**** Ki67 immunostaining of PC3 xenografts, DU145 xenografts, and LNCaP xenografts. Scale = 20 µm.

In order to firmly establish a role for *GHSROS* in tumor growth, we established subcutaneous *GHSROS*-overexpressing androgen-independent (PC3 and DU145) and androgen-responsive (LNCaP) cell line xenografts in NOD/SCID IL2R *γ* (NSG) mice. Subcutaneous graft sites allow easy implantation and monitoring of tumor growth (using calipers) (Lin et al., 2014)—ideal for exploring the role of a new gene such as *GHSROS* in vivo. Overexpression of *GHSROS* in xenografts was confirmed post-mortem by qRT-PCR (Fig. S8). Compared to vector controls, xenograft tumor volumes were significantly greater at day 25 in PC3-GHSROS mice (*P* = 0.0040, Mann–Whitney-Wilcoxon test) and at day 35 in DU145-GHSROS mice (*P* = 0.0011) (Figs. 4A, 4B). While xenograft tumors were not palpable in LNCaP-GHSROS mice in vivo, tumors were significantly larger by weight post-mortem (at 72 days) (*P* = 0.042) (Fig. 4C)—with a size increase similar to that seen for DU145-GHSROS xenografts (Fig. 4D). LNCaP-GHSROS tumors invaded the flank muscle and the peritoneum (data not shown) and were more vascularized than control tumors (observed grossly and estimated by CD31^+^ immunostaining) (Fig. 4G). Representative Ki67 immunostaining for proliferating xenograft tumor cells is shown in Fig. 4H.

### *GHSROS* modulates the expression of cancer-associated genes

Having established that *GHSROS* plays a role in regulating hallmarks of cancer—including cell proliferation, invasion, and migration (Hanahan & Weinberg, 2011)—we sought to determine the genes likely to mediate its function by examining the transcriptomes of cultured PC3 cells and LNCaP xenografts overexpressing this lncRNA.

**Figure 5:**
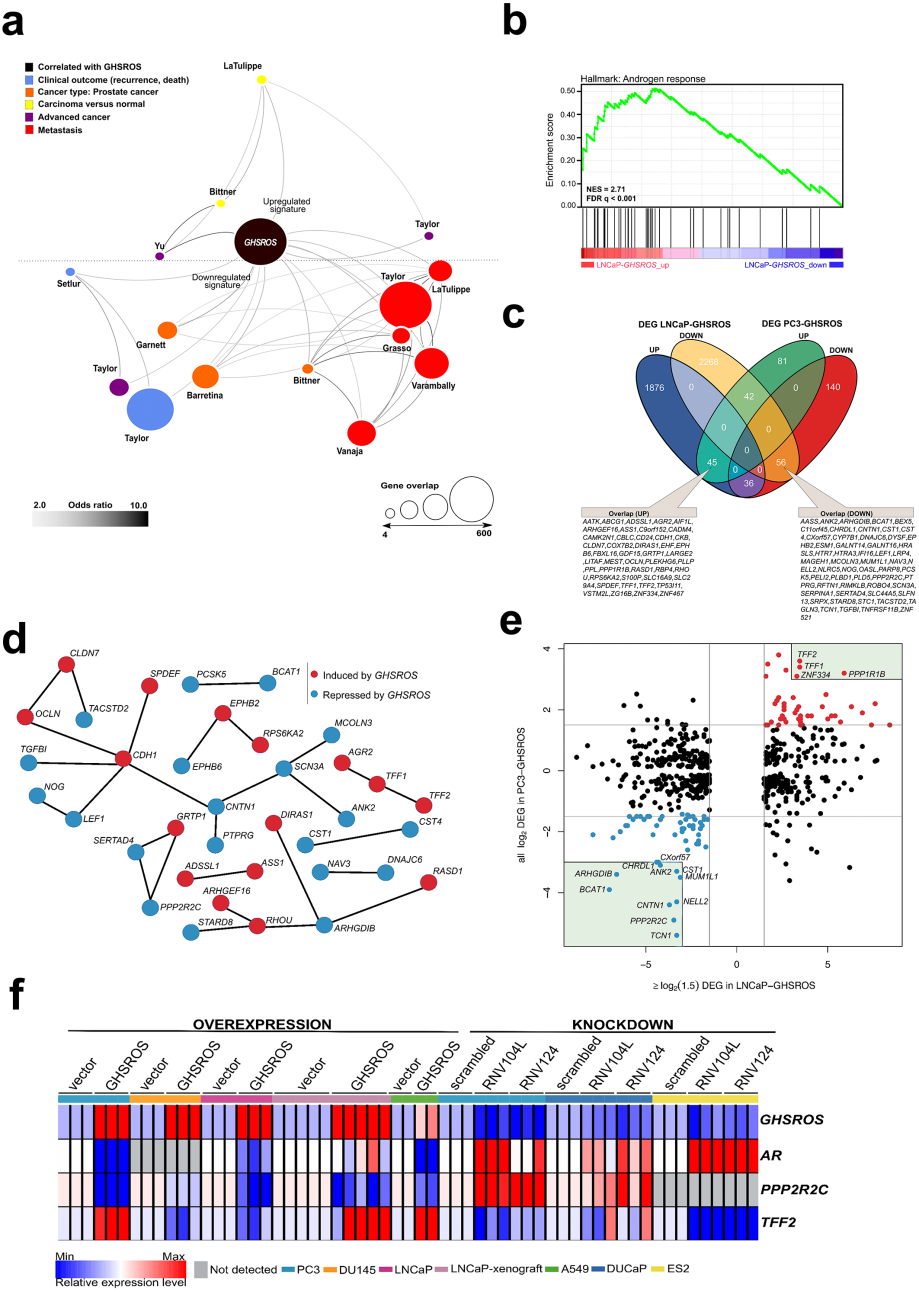
*GHSROS* overexpression modulates the expression of cancer-associated genes. (A) Oncomine network representation of genes differentially expressed by cultured PC3-GHSROS cells visualized using Cytoscape. Node sizes (gene overlap) reflect the number of genes per molecular concept. Nodes are colored according to concept categories indicated in the left corner. Edges connect enriched nodes (odds ratio ≥ 3.0) and darker edge shading indicates a higher odds ratio. (B) Gene set enrichment analysis (GSEA) of genes differentially expressed by LNCaP-GHSROS xenografts reveals enrichment for the androgen response. The normalized enrichment score (NES) and GSEA false-discovery corrected *P*-value (*Q*) are indicated. (C) Venn diagram of differentially expressed genes (DEG) in LNCaP-GHSROS and PC3-GHSROS cells. Symbols of 101 overlapping genes are indicated in text boxes. (D) Interaction of 101 genes differentially expressed in PC3-GHSROS and LNCaP-GHSROS cells (see (C)). Lines represent protein-protein interaction networks from the STRING database. Genes induced (red) or repressed (blue) by *GHSROS*-overexpression are indicated. (E) Gene expression scatter plot comparing *GHSROS*-overexpressing PC3 and LNCaP cells. Differentially expressed genes (DEGs) in both datasets shown in red (induced) and blue (repressed); of which ≥ 8-fold (log_2_ cutoff at −3 and 3) DEGs are highlighted by a green box. (F) Heat map of gene expression in *GHSROS*-perturbed cells. Each row shows the relative expression of a single gene and each column a sample (biological replicate). Fold-enrichment of each gene normalized to *RPL32* and compared to empty vector control (overexpression) or scrambled control (LNA-ASO knockdown). Fold-changes were log_2_-transformed and are displayed in the heat map as the relative expression of a gene in a sample compared to all other samples (*Z*-score).

High-throughput RNA-seq of cultured PC3-GHSROS cells (∼50M reads) revealed that 400 genes were differentially expressed (168 upregulated and 232 downregulated; moderated *t*-test; cutoff set at log_2_ fold-change ±1.5, *Q* ≤0.05) (Table S3) compared with empty vector control cells. Supporting our functional data, gene ontology analysis (using DAVID) showed enrichment for processes such as epithelial structure maintenance, response to hormone stimulus, steroid hormone stimulus, estradiol stimulus, response to hypoxia, and drug response (Tables S4 and S5). Given that *GHSROS* is not readily detectable by high-throughput sequencing and array technologies, we queried the 400 genes differentially expressed in PC3-GHSROS cells by Oncomine concept map analysis (Hodes et al., 2007). Enriched Oncomine concepts included poor clinical outcome and metastatic progression (Fig. 5A; Table S6).

Complementary lower-coverage (∼30M reads) RNA-seq data from LNCaP-GHSROS xenografts demonstrated that a surprisingly large number of genes were differentially expressed (1,961 upregulated, 2,372 downregulated, moderated *t*-test; cutoff set at log_2_ fold-change ±1.5, *Q* ≤ 0.05) (Dataset S1). Selected genes with low expression counts were validated by qRT-PCR (Fig. S9). In LNCaP-GHSROS xenografts, *GHSROS*-regulated genes were enriched for the androgen response (gene set enrichment analysis; NES = 2.71, *Q* ≤ 0.001) (Fig. 5B), and included PSA (*KLK3*) (750.9-fold, *Q* = 3.6 × 10^−6^) and transmembrane protease serine 2 (*TMPRSS2*) (335.4-fold, *Q* = 4.5 × 10^−6^) (Dataset S2). We also observed downregulation of numerous genes associated with cell migration and adhesion, epithelial–mesenchymal transition (EMT) (including *ZEB1;* -97.0-fold, *Q* = 1.5 × 10^−5^), and angiogenesis and vasculature development (Dataset S3). As mentioned above, subcutaneous LNCaP-GHSROS xenografts infiltrated flank muscle and the peritoneum and were more vascularized at 72 days post injection in NSG mice, which may indicate that these invasive tumors may have undergone EMT and promoted angiogenesis.

It is appreciated that the bone metastasis-derived, androgen-independent PC3 and the lymph node metastasis-derived, androgen-responsive LNCaP prostate cancer cell lines represent genetically and presumably metabolically distinct subtypes (Seim et al., 2017). They are therefore useful for revealing broad, functional gene expression changes associated with aggressive disease in forced overexpression and knockdown experiments. Despite the differences between these cell lines, a quarter (25.3%; 101) of genes differentially expressed by PC3 cells overexpressing *GHSROS* were also differentially expressed by LNCaP-*GHSROS* cells (Fig. 5C) (*P* = 0.000020, hypergeometric test). These genes represent candidate mediators of *GHSROS* function.

We interrogated the STRING database (Gao et al., 2013) to reveal protein interactions between the 101 genes regulated by *GHSROS* in both cell lines. A number of genes associated with cell–cell adhesion, migration, and growth were connected, indicating functional enrichment of these proteins in *GHSROS*-overexpressing prostate cancer cells (Fig. 5D). This included increased expression of epithelial cadherin (*CDH1*), occludin (*OCLN*), and claudin-7 (*CLDN7*); and decreased contactin 1 (*CNT1*), noggin (*NOG*), and transforming growth factor beta induced (*TGFBI*) in *GHSROS*-overexpressing cells. Of note, increased *CDH1* expression is associated with exit from EMT and growth of aggressive, metastatic prostate tumors (Putzke et al., 2011). A second, interesting upregulated network included anterior gradient 2 (*AGR2*) and trefoil factors 1 and 2 (*TFF1* and *TFF2*). Trefoil factors are small proteins associated with mucin glycoproteins. Their expression is increased in castration-resistant prostate cancer (CRPC) and may facilitate the acquisition of hormone independence (Vestergaard et al., 2006; Kani et al., 2013). Similarly, *AGR2* has been associated with the propensity of a number of aggressive tumor types to metastasize, including prostate cancer (Zweitzig et al., 2007).

Ten out of the 101 genes were differentially expressed in metastatic tumors compared to primary tumors in two clinical prostate datasets: Grasso (Taylor et al., 2010) (59 localized and 35 metastatic prostate tumors) and Taylor (Benjamini & Hochberg, 1995) (123 localized and 27 metastatic prostate tumors) (Tables S7 and S8) (*Q* ≤ 0.25, moderated *t*-test). *DIRAS1*, *FBXL16*, *TP53I11*, *TFF2*, and *ZNF467* were upregulated in both metastatic tumors and *GHSROS*-overexpressing PC3 and LNCaP cells, while *AASS*, *CHRDL1*, *CNTN1*, *IFI16*, and *MUM1L1* were downregulated. We investigated whether the expression of these genes contributes to adverse disease outcome by assessing survival in two independent datasets: the Taylor dataset and TCGA-PRAD. The latter is a dataset of localized prostate tumors generated by The Cancer Genomics Atlas (TCGA) consortium (Casper et al., 2015; Cancer Genome Atlas Research Network, 2015). As overall survival data was available for a small number of patients in these datasets, we assessed disease-free survival (relapse). Relapse is a suitable surrogate for overall survival in prostate cancer given that recurrence of disease would be expected to contribute significantly to mortality, and metastatic disease is incurable. Unsupervised *k*-means clustering was employed to divide each dataset into two groups based on gene expression alone. Two genes, zinc finger protein 467 (*ZNF467*; which was induced by forced *GHSROS*-overexpression) and chordin-like 1 (*CHRDL1*; which was repressed), correlated with relapse in both datasets (Table S9). Chordin-like 1 is a negative regulator of bone morphogenetic protein 4-induced migration and invasion in breast cancer (Cyr-Depauw et al., 2016). It was downregulated in *GHSROS*-overexpressing cell lines and in metastatic tumors compared to localized tumors in the Taylor and Grasso datasets. Interrogation of the Chandran prostate cancer dataset (60 localized tumors and 63 adjacent, normal prostate) (Chandran et al., 2005) suggests that *CHRDL1* is downregulated by prostate tumors in general. *CHRDL1* expression stratified the Taylor (*N* = 150; 27 metastatic tumors) dataset into two groups with a significant, 438-day difference in overall disease-free survival (relapse; Cox *P* = 0.0062, absolute hazard ratio (HR) = 2.5). A statistically significant, yet clinically negligible, difference in relapse (9 days; Cox *P* = 0.0071, absolute HR = 1.8) was observed in the TCGA-PRAD dataset (*N* = 489; no metastatic tumors) (Table S9). Survival analysis *P*-values (Kaplan–Meier and Cox proportional-hazard) and hazard ratios indicate whether there is a significant difference between two groups, but not the degree of difference. Evaluating statistically significant differences in survival (e.g., in days) between groups is therefore subjective. Given these data, we propose that *CHRDL1* may play an important role in metastatic tumors.

In contrast to *CHRDL1*, *ZNF467* stratified patients into clusters with an obvious difference in overall median survival (relapse) between groups in both the Taylor (697 days; Cox *P* = 0.0039, HR = 2.7) and TCGA-PRAD datasets (139 days; Cox *P* = 0.000026, HR = 2.5) (Table S9). ZNF467 has not been functionally characterized, however, a recent study suggests that it is a transcription factor which clusters in close proximity to the androgen receptor in a network associated with breast cancer risk (Castro et al., 2016), indicating that ZNF467 and AR regulate similar pathways. Clustering of patients into groups of either low or high *ZNF467* expression revealed that elevated expression of the gene associated with a worse relapse outcome (Fig. S10A–Fig. S10C). In agreement, *ZNF467* gene expression can distinguish low (≤6) from high (≥8) Gleason score prostate tumors in a Fred Hutchinson Cancer Research Center prostate cancer dataset (381 localized and 27 metastatic prostate tumors) (Jhun et al., 2017). *ZNF467* expression is also elevated in chemotherapy-resistant ovarian cancer (Zhu et al., 2015) and breast cancer (Davies et al., 2014) cell lines.

The 101 *GHSROS*-regulated genes were visualized in a scatter plot to reveal genes with particularly distinct (here ∼8-fold) differential expression in *GHSROS*-overexpressing prostate cancer cell lines –putative fundamental drivers of the observed tumorigenic phenotypes. This revealed that *PPP2R2C* (Fig. 5E), a gene encoding a subunit of the holoenzyme phosphatase 2A (PP2A) (Bluemn et al., 2013; Gonzalez-Alonso et al., 2015), was downregulated by forced overexpression of *GHSROS*. In the PC3-GHSROS RNA-seq dataset, *PPP2R2C* was the third most downregulated gene (−29.9-fold, moderated *t*-test *Q* = 3.4 × 10^−10^) (Table S3). Consistently, forced overexpression or knockdown of *GHSROS* in prostate cancer cell lines reciprocally regulated endogenous *PPP2R2C* expression (Fig. 5F; Figs. S9 and S11).

We observed that *GHSROS* was also able to reciprocally regulate androgen receptor (*AR*) expression in some prostate cancer cell lines (downregulated upon *GHSROS* overexpression in PC3 and LNCaP; upregulated upon *GHSROS* knockdown in DUCaP) (Fig. 5F). LNCaP-GHSROS xenografts showed a variable *AR* expression pattern, which may be linked to differences in available androgen, however, *PPP2R2C* expression was still significantly repressed in vivo (−3.7-fold, Student’s *t*-test *P* = 7.9 × 10^−3^) (Fig. S9). Similarly, while *AR* could not be detected in DU145 cells, *GHSROS*-overexpression decreased *PPP2R2C* expression in this cell line (Fig. 5F). The androgen receptor is also expressed by ovarian and lung cancer tumors and cell lines (Zhu et al., 2017; Harlos et al., 2015). Forced overexpression of *GHSROS* in the A549 lung adenocarcinoma cell line decreased *AR* and *PPP2R2C* expression (Student’s *t*-test, *P* ≤ 0.0001). *GHSROS* knockdown in the ES-2 ovarian clear cell carcinoma cell line, which does not express *PPP2R2C*, increased the expression of *AR* (Student’s *t*-test, *P* = 0.0029 and *P* = 0.0022) (Fig. 5F; Fig. S11).

## Discussion

Recent work suggests that a small proportion (∼3%) of long non-coding RNA genes are dysregulated in tumors and mediate cell growth (Liu et al., 2017). Herein, we demonstrate that the lncRNA *GHSROS* is one such gene. *GHSROS* expression is elevated across many different cancers, suggesting that it is a so-called pan-cancer lncRNA (Chiu et al., 2018; Cabanski et al., 2015). In prostate cancer *GHSROS* is detectable in normal tissue and expressed at higher levels in a subset (∼10%) of tumors. We have yet to determine which subgroups of prostate cancer demonstrate elevated *GHSROS*, however.

From assessing the function of *GHSROS* in immortalized prostate cancer cell lines we observed that forced overexpression of *GHSROS* enhances in vivo tumor growth and invasion, and in vitro cell viability and motility. We also demonstrate that forced overexpression of *GHSROS* facilitates survival and recalcitrance to the cytotoxic chemotherapy drug docetaxel. Critically, we show that endogenous *GHSROS* is elevated following docetaxel treatment. Docetaxel is commonly prescribed for late-stage, metastatic CRPC patients, but large, randomized trials suggest that it is also effective against recently-diagnosed, localized prostate tumors (Puente et al., 2017). These data suggest that *GHSROS* acts as a cell survival factor in prostate cancer. While the underlying mechanisms are unknown, two genes associated with chemotherapy resistance, *ZNF467* and *PPP1R1B* (also known as *DARPP-32*), were upregulated in PC3 and LNCaP cells overexpressing *GHSROS*. *PPP1R1B* is a potent anti-apoptotic gene which confers resistance in cancer cell lines to several chemotherapeutic agents when overexpressed (Belkhiri, Zhu & El-Rifai, 2016).

The expression and function of *GHSROS* in prostate cancer suggests that it belongs to a growing list of lncRNAs that function as *bona fide* oncogenes. Notable examples associated with aggressive cancer and adverse outcomes include *HOTAIR* (HOX transcript antisense RNA), which is upregulated in a range of cancers (Huarte, 2015), and the prostate cancer-specific *SCHLAP1* (SWI/SNF Complex Antagonist Associated With Prostate Cancer 1) (Prensner et al., 2013). To better understand how *GHSROS* mediates its effects in prostate cancer, we examined transcriptomes of prostate cancer cell lines with forced *GHSROS* overexpression: PC3 cells in culture (in vitro) and subcutaneous LNCaP xenografts in mice (in vivo). The 101 common differentially expressed genes included several transcription factors with established roles in prostate cancer and genes associated with metastasis and poor prognosis. Our study not only highlights genes modulated by *GHSROS*, but also genes (such as *ZNF467*, *CHRDL1*, and *PPP2R2C*) that may be generally relevant to prostate cancer progression.

Reactivation of the androgen receptor (*AR*) has long been considered a seminal event; supporting renewed tumor growth in a majority of metastatic CRPC patients (Ferraldeschi et al., 2015; Wyatt & Gleave, 2015). However, it is now increasingly recognized that, similar to other endocrine-related cancers, several subtypes of prostate cancer exist (Tosoian & Antonarakis, 2017; Shoag & Barbieri, 2016; Dagogo-Jack & Shaw, 2018). These include subtypes characterized by androgen pathway-independent growth (Bluemn et al., 2013; Wyatt & Gleave, 2015; luemn et al., 2017). In this context, our results on *PPP2R2C,* a gene which encodes a PP2A substrate-binding regulatory subunit, is of interest. We demonstrate that *PPP2R2C* expression in prostate cancer cell lines is repressed by forced *GHSROS* overexpression and increased by *GHSROS* knockdown. There is emerging evidence that inactivation of PP2A mediates CRPC in a subset of patients who display resistance to AR-targeting therapies (Bluemn et al., 2013; Gonzalez-Alonso et al., 2015). Loss of *PPP2R2C* expression alone is thought to reprogram prostate tumors towards AR pathway-independent growth and survival (Bluemn et al., 2013). Several independent lines of evidence suggest that *PPP2R2C* is a critical tumor suppressor involved in many cancers. Loss of *PPP2R2C* expression has been attributed to esophageal adenocarcinoma tumorigenesis (Peng et al., 2017), and *PPP2R2C* downregulation by distinct microRNAs positively correlates with increased proliferation of cultured cancer cells derived from the prostate (Bi et al., 2016), nasopharynx (Yan et al., 2017), and ovary (Wu et al., 2016). *PPP2R2C* also has a classical growth-inhibiting tumor suppressor role in brain cancers (Fan et al., 2013). A subtype of medulloblastoma, pediatric brain tumors, are characterized by high expression of the chemokine receptor CXCR4 and concordant suppression of *PPP2R2C* (Sengupta et al., 2012). Similarly, the gene is ablated in A2B5^+^ glioma stem-like cells, a population which mediates a particularly aggressive chemotherapy-resistant glioblastoma phenotype (Auvergne et al., 2013). Although seemingly paradoxical, *GHSROS* repression of *AR* and *PPP2R2C* in prostate cancer cell lines can be rationalized. Knockdown of *PPP2R2C* using small interfering RNA in cultured LNCaP and VCaP cells did not alter the expression of *AR* (Bluemn et al., 2013). In contrast, *AR* knockdown in androgen-independent LP50 cells (Gonzalez-Alonso et al., 2015) (a cell line derived from LNCaP) markedly decreased *PPP2R2C* expression (Fig. S12)—suggestive of an adaptive response to loss of androgen receptor expression (and function). Precisely how *GHSROS* mediates *PPP2R2C* downregulation and its effects on tumor growth remains to be determined, however, *GHSROS* is the first lncRNA shown to downregulate this critical tumor suppressor, suggesting a role in adaptive survival pathways and CRPC development. Taken together, we speculate that *GHSROS* primes prostate tumors for androgen receptor-independent growth.

In this study, the growth of *GHSROS*-overexpressing prostate cancer cell lines was assessed using subcutaneous prostate cancer cell lines xenografts. We appreciate that other models (including orthotopic xenografts) are critical for firmly establishing roles for a gene in cancer processes, including invasion and metastasis (Sonpavde et al., 2015), and we will assess these in a future study. An additional limitation to the present study is the relatively high levels of ectopically expressed *GHSROS* in our cell line models. To complement our overexpression studies, functional assays using modified ASOs in a range of prostate cell lines and patient-derived xenografts (PDX) should be conducted in vitro and in vivo. We recently performed in vivo experiments using LNA ASOs targeting *GHSROS* and observed no signs of toxicity or weight loss in mouse models (Thomas et al., unpublished data). This is similar to findings with other LNA ASOs of comparable lengths (16-mer to 21-mer) (Chi et al., 2005; Emmrich et al., 2009). The interaction between *GHSROS* and genomic regions, proteins, and other RNA transcripts also requires further elucidation. While this study firmly establishes that *GHSROS* plays a role in prostate cancer, the mechanism by which it reprograms gene expression remains unknown. LncRNAs are now considered critical components of the cellular machinery (Mattick & Rinn, 2015). Unlike protein-coding genes, which typically require sequence conservation to maintain function, the mechanisms of action of lncRNAs are usually not obvious and uncovering their precise, sometimes subtle, function remains a challenge (Mattick & Rinn, 2015). For example, some lncRNAs modulate the epigenetic regulation of gene expression and interact with chromatin, acting as scaffolds to guide other molecules (including RNA, proteins, and epigenetic enzymes) to influence gene expression (Mattick & Rinn, 2015; Huarte, 2015).

Although cancers are highly heterogeneous diseases and few therapies target molecular phenotypes, lncRNAs provide a largely untapped source for new molecular targets (Huarte, 2015). Here, we developed antisense oligonucleotides targeting *GHSROS* and assessed them in cultured cancer cells. We are currently refining our LNA oligonucleotides and their delivery for targeting in vivo xenografts and prostate cancer patient-derived organoids in order to further assess their clinical potential. Targeting *GHSROS* may present an opportunity for clinical intervention, however, it is appreciated that translational and regulatory challenges exist for oligonucleotide therapies (Stein & Castanotto, 2017).

In summary, we propose that *GHSROS* is an oncogene that regulates cancer hallmarks and the expression of a number of genes, including the tumor suppressor *PPP2R2C –* the loss of which is an emerging alternative driver of prostate cancer. Further studies are needed to elucidate the expression and function of *GHSROS* in more detail and to determine whether pharmacological targeting of this lncRNA could prove useful for treating cancer.

##  Supplemental Information

10.7717/peerj.10280/supp-1 Supplemental Information 1 Differentially expressed genes in LNCaP-GHSROS cells. Compared to empty vector control Red: higher expression in LNCaP-GHSROS cells; Black: lower expression in LNCaP-GHSROS cells. Fold-changes are log2 transformed; *Q*-value denotes the false discovery rate (FDR; Benjamini-Hochberg)-adjusted *P*-value (cutoff ≤ 0.05).

10.7717/peerj.10280/supp-2 Supplemental Information 2 Enrichment for GO terms in the category ‘biological process’ for genes upregulated in LNCaP-GHSROS cells (compared to empty-vector control) *P* ≤ 0.01, Fisher’s exact test

10.7717/peerj.10280/supp-3 Supplemental Information 3 Enrichment for GO terms in the category ‘biological process’ for genes downregulated in LNCaP-GHSROS cells (compared to empty-vector control) *P* ≤ 0.01, Fisher’s exact test

10.7717/peerj.10280/supp-4 Supplemental Information 4 Supplementary information

## Additional Information and Declarations

### Data Availability

The following information was supplied regarding data availability: The data are available at NCBI GEO: GSE86097, GSE103320. The code is available in a repository at GitHub: https://github.com/sciseim/GHSROS_MS.
