# Supplementary material for: The long non-coding RNA GHSROS reprograms prostate cancer cell lines toward a more aggressive phenotype
Source: PeerJ. 2021 Feb 1;9:e10280. doi: 10.7717/peerj.10280 (PMC7860111; doi:10.7717/peerj.10280)
Supplement: Supplemental Information 4 [file peerj-09-10280-s004.pdf]

Supplementary information

The long non-coding RNA *GHSROS* reprograms prostate cancer cell lines toward a more aggressive phenotype

Patrick B. Thomas[1,2,3], Penny L. Jeffery[1,2,3], Manuel D. Gahete[4,5,6,7,8], Eliza J. Whiteside[9,10,†], Carina Walpole[1,3], Michelle L. Maugham[1,2,3], Lidija Jovanovic[3], Jennifer H. Gunter[3], Elizabeth D. Williams[3], Colleen C. Nelson[3], Adrian C. Herington[1,3], Raúl M. Luque[4,5,6,7,8], Rakesh N. Veedu[11], Lisa K. Chopin[1,2,3,*], Inge Seim[1,2,3,12,*]

[1] Ghrelin Research Group, Translational Research Institute- Institute of Health and Biomedical Innovation, School of Biomedical Sciences, Queensland University of Technology (QUT), Woolloongabba, Queensland 4102, Australia.

[2] Comparative and Endocrine Biology Laboratory, Translational Research Institute-Institute of Health and Biomedical Innovation, School of Biomedical Sciences, Queensland University of Technology, Woolloongabba, Queensland 4102, Australia.

[3] Australian Prostate Cancer Research Centre–Queensland, Institute of Health and Biomedical Innovation, School of Biomedical Sciences, Queensland University of Technology (QUT), Princess Alexandra Hospital, Translational Research Institute, Woolloongabba, Queensland 4102, Australia.

[4] Maimonides Institute of Biomedical Research of Cordoba (IMIBIC), Córdoba, 14004, Spain.

[5] Department of Cell Biology, Physiology and Immunology, University of Córdoba, Córdoba, 14004, Spain.

[6] Hospital Universitario Reina Sofía (HURS), Córdoba, 14004, Spain.

[7] CIBER de la Fisiopatología de la Obesidad y Nutrición (CIBERobn), Córdoba, 14004, Spain.

[8] Campus de Excelencia Internacional Agroalimentario (ceiA3), Córdoba, 14004, Spain.

[9] Centre for Health Research, University of Southern Queensland, Toowoomba, Queensland 4350, Australia.

[10] Institute for Life Sciences and the Environment, University of Southern Queensland, Toowoomba, Queensland 4350, Australia.

[11] Centre for Comparative Genomics, Murdoch University & Perron Institute for Neurological and Translational Science, Perth, Western Australia 6150, Australia.

[12] Integrative Biology Laboratory, College of Life Sciences, Nanjing Normal University, 1 Wenyuan Road, 210023, Nanjing, China.

[†] Present address: Moffitt Cancer Center and Research Institute, Tampa, FL, 33612, USA.

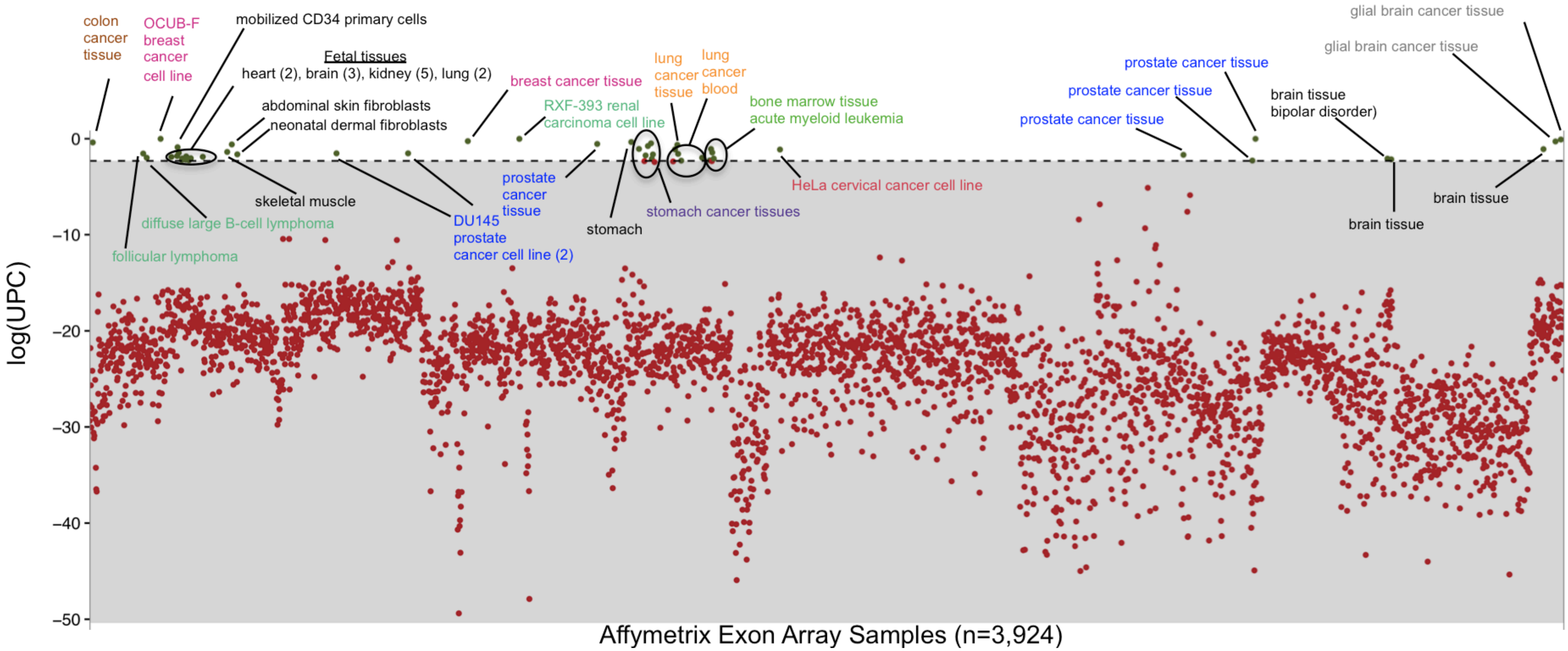

Supplementary Fig. S1. Scatterplot of *GHSROS* Universal exPression Code values in publicly available exon array datasets. The scatter plot shows the log of Universal exPression Code (UPC) values, an estimate on whether a gene is actively transcribed in exon array samples. The dotted horizontal line separates samples with a UPC ≥ 0.1.

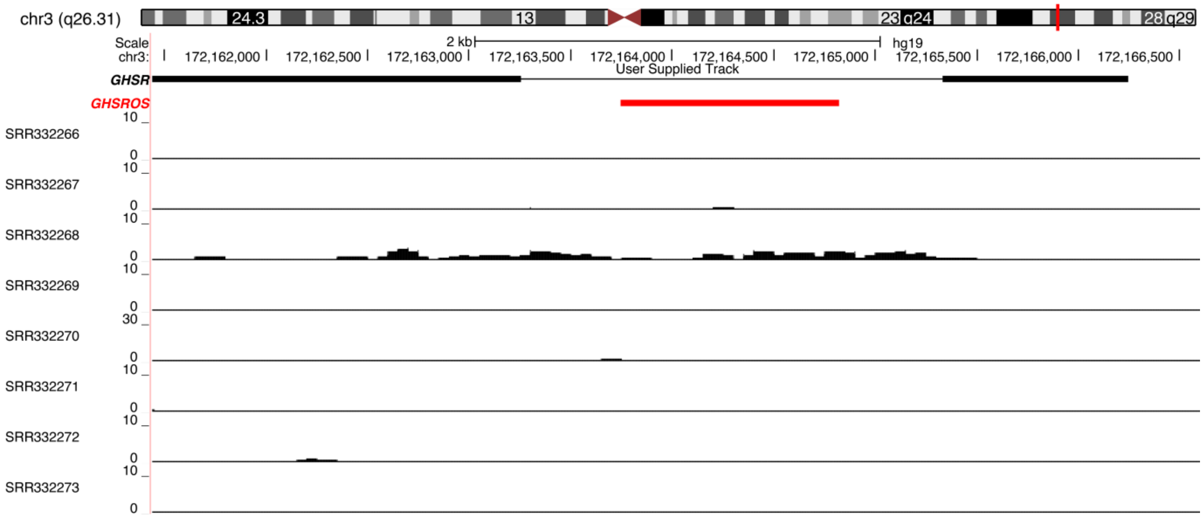

Supplementary Fig. S2. UCSC genome browser visualization of *GHSR*/*GHSROS* locus expression in castration-resistant prostate cancer. *GHSR* exons (black), antisense *GHSROS* exon (red). SRR332266 to SRR332273 denote NCBI Sequence Read Archive (SRA) database accession numbers. The *y*-axis represents read counts normalized to sequencing depth.

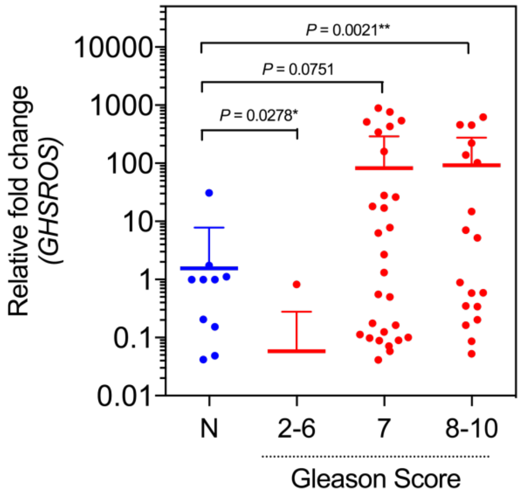

Supplementary Fig. S3. *GHSROS* expression in OriGene TissueScan Prostate Cancer Tissue qPCR panels stratified by Gleason score. *$P \leq 0.05$, **$P \leq 0.01$, Mann-Whitney-Wilcoxon test. Expression was normalized to the housekeeping gene *RPL32* and relative to a normal prostate sample.

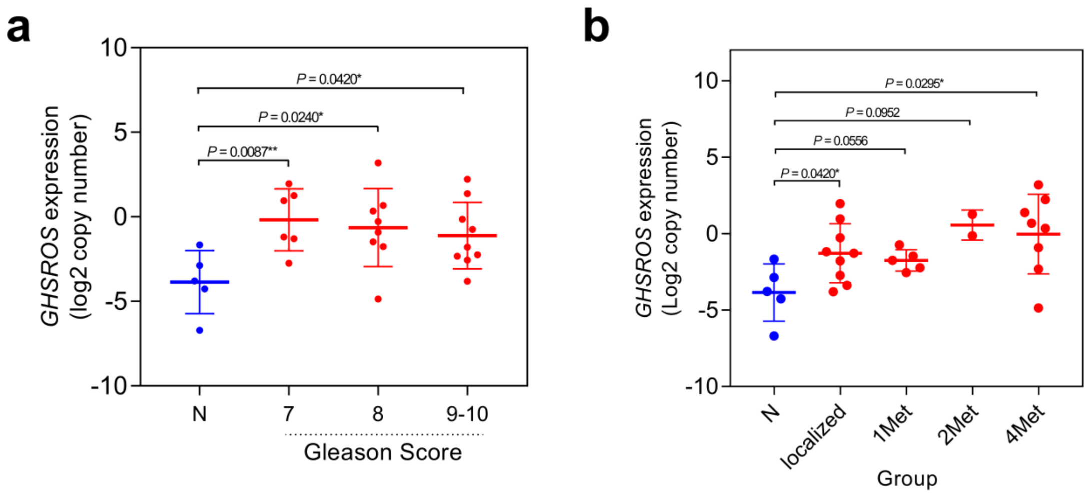

Supplementary Fig. S4. *GHSROS* expression in the Andalusian Biobank prostate tissue cohort. *GHSROS* expression in the Andalusian Biobank prostate tissue cohort stratified by **(a)** Gleason score and **(b)** number of metastatic sites. 1 Met denotes one and ≥ 2 Met two or more metastatic sites. Absolute expression levels were determined by qRT-PCR and adjusted by a normalization factor calculated from the expression levels of three housekeeping genes (*HPRT*, *ACTB*, and *GAPDH*). N denotes normal prostate. $^*P \leq 0.05$, $^{**}P \leq 0.01$, Mann-Whitney-Wilcoxon test.

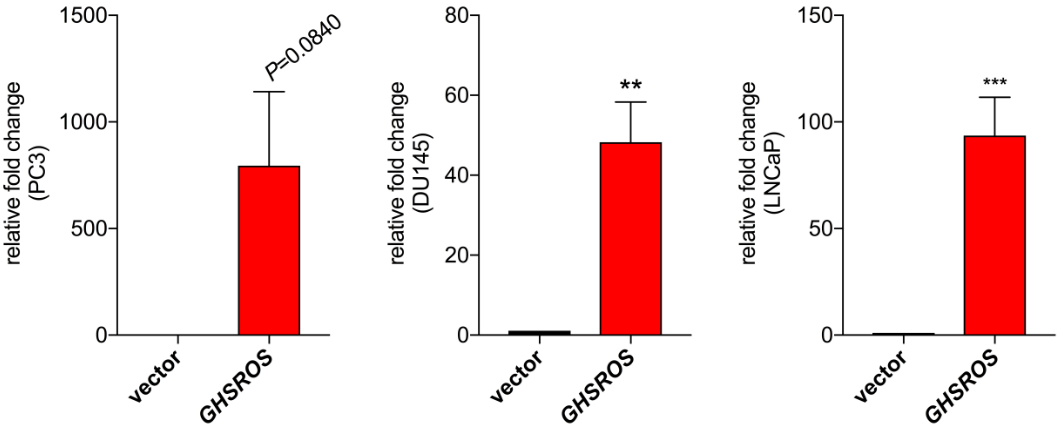

Supplementary Fig. S5. Confirmation of *GHSROS* overexpression in prostate cancer-derived cell lines. Bar graphs show qRT-PCR quantification of the relative expression levels of *GHSROS* when overexpressed in prostate-derived (PC3, DU145, and LNCaP) cancer cell lines. Expression was normalized to the housekeeping gene *RPL32* using the comparative $2^{-\Delta\Delta Ct}$ method of quantification. Results are relative to the respective vector control. Mean ± s.e.m., *n*=3, $^{**}P \leq 0.01$, $^{***}P \leq 0.001$, Student's *t*-test.

```
GUUUCACAGAGGAUUGAAUAUACAGUUAGGAUUAAGAAUCACUGAGAUGAAUGAUUCUAU 60
GCCACUUACAAUAGACAUAAGUAAGGCUAUUAUUAAACCAACUCAUAUAAAUUAUCAUCC 120
AUCCAAGUCUUCAUUUUGCCUAUGUAAGAUAAUUUUUAAUGAUGUCUAAAUGUUUAGAUU 180
GUUUUAAAUUCAGAAAUCAGGGAAAAAUAAUAACAAAUAAUUUUUUAGUCGUUGUAACAC 240
UUUAGUGAUCCCCUUCAAAUUGUCAAGGAAGAAGAAAGUUAACAUUGGUGUUGAGACACC 300
AAUGAAGAAAUUAAUGGCUUGAAAAUAAAAUUGGAGGAAAUUAUUAAACUUAUCCUUAAA 360
ACUAAGUUGUAUAGUAGCAACUUAACUGCCACAGUAACAAUGUGACUCUCGUAGGUGAGA 420
GCUUAGGAUCCCUUGGAGGACACUAGCAGGUUUAUAACCUAUGAGUCAUAUCACGAGAAU 480
CACCGGUCCAGAAAAUAAAAUCUUUGACAUUCCUUGAAUAAACUCUAUCACUUUAAUAUG 540
GAGGAUCCAGGAACUGUAAAAUUGGAAAUAUGAAAUCAUCCUGGAAUGGCUUCUAAGAAG 600
CCUUAAAAUAUGCCAUUAAGUAUGAAGUCUUGACUAUAAUAUCUAAAUAAUAAAAUAUAC 660
AGACCUUGGUUUUGAAAAUGCAAGUAGAGUUAAUAUUUUGCUGGGCUGUAACCAUAUUUA 720
UUAUUACUUCACUUGUACCAAACACAUAUACUUAAAGAAGCUGAAUUUGUGCAAUAAACA 780
UUCAGCAAAUCCAGUUAUGACAUUGUGCCCAGAUGUUCCUGAAGGGUCAUUUGGGGAAGU 840
AGUGAAUUCUGUUGGUUUCCACAAAGUCUCCUUUCCUCCUGUUGGAGCUUUGUUUAAUCU 900
AGUUUAUCCUUACCUUAUCCUAGUCUAUCCUACCUGAAGAGGCAGGUAAUCAUAAAAACA 960
AAUGAGACUUCACUGAUUUGUCACUGACUUCCUUACAAAGCUGUAUGAACAGCAGCAGGG 1020
UAAACAGAAUUGAUGCAAUUAUCUGAAAAAAUGCAGGGCAGAAAACAAAUCACAAUAUUG 1080
AAAGAAAAAUGAAUAAUCUC                                         1100
```

ASO LNA RNV104L and RNV124

qRT-PCR amplicon

Supplementary Fig. S6. Sequence of the lncRNA *GHSROS* and regions targeted by oligonucleotides. The 1.1kb *GHSROS* sequence. Locked nucleic antisense oligonucleotide (LNA-ASO) locations are highlighted in red and the qRT-PCR amplicon in yellow.

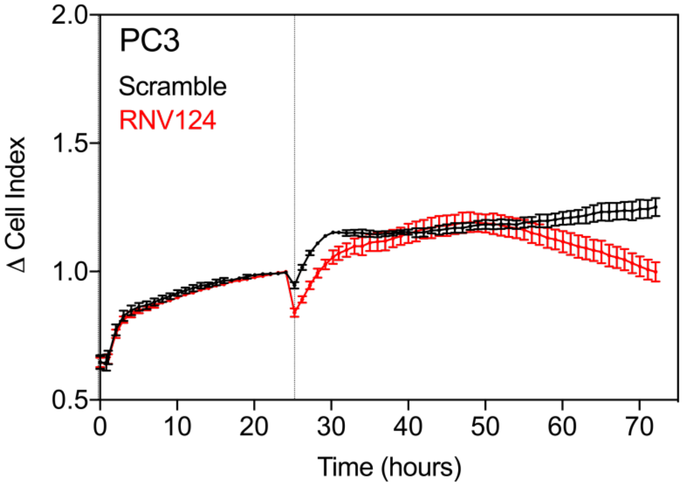

Supplementary Fig. S7. *GHSROS* knockdown attenuates PC3 cell survival upon serum starvation. Cells were transfected with the LNA-ASO RNV124 and grown for 24 hours (indicated by a vertical dotted line) prior to serum starvation. Results are relative to scrambled control. Mean ± s.e.m., n=3. At 48 hours after serum starvation, (72 hours after cells transfected as indicated on the x-axis), there was a significant difference in survival (P = 0.049, Student's t-test).

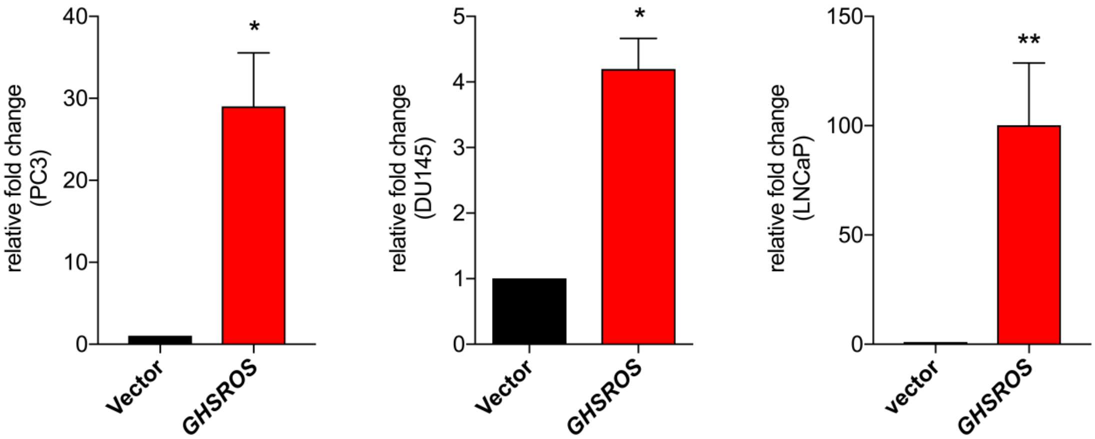

Supplementary Fig. S8. Validation of *GHSROS* overexpression in PC3, DU145, and LNCaP tumor xenografts by qRT-PCR. Expression changes were measured from excised PC3 (*n*=2 vector, *n*=3 *GHSROS*), DU145 (*n*=2 vector, *n*=3 *GHSROS*), and LNCaP xenografts (*n*=8 vector, *n*=5 *GHSROS*) at *in vivo* endpoint. Expression was normalized to the housekeeping gene *RPL32*. Results are relative to the respective vector control. Mean ± s.e.m., *n*=3, *$P \leq 0.05$, **$P \leq 0.01$, Student's *t*-test.

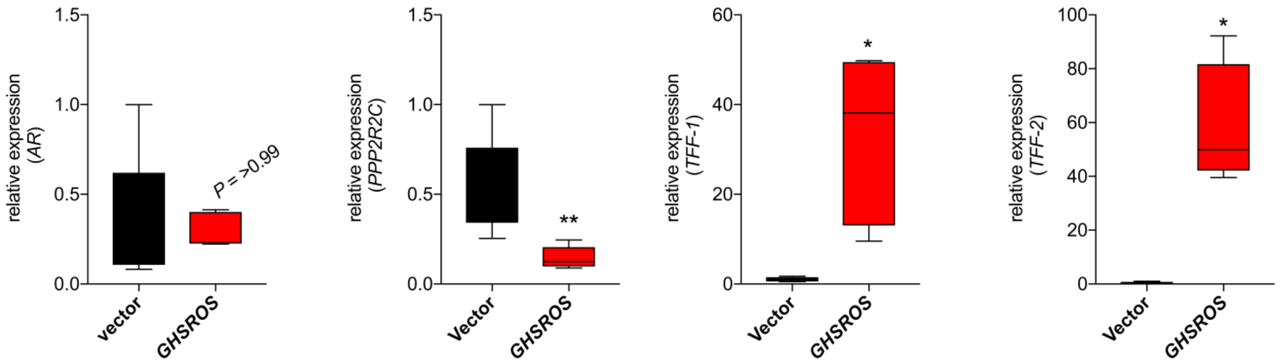

Supplementary Fig. S9. Expression of selected genes in LNCaP tumor xenografts overexpressing *GHSROS*. Expression changes were measured by qRT-PCR from excised LNCaP (n=4-8 vector, n=4-5 *GHSROS*) at *in vivo* endpoint. Expression was normalized to the housekeeping gene *RPL32*. Results are relative to the respective vector control. Mean, n=3, *$P \leq 0.05$, ***$P \leq 0.01$, Student's t-test.

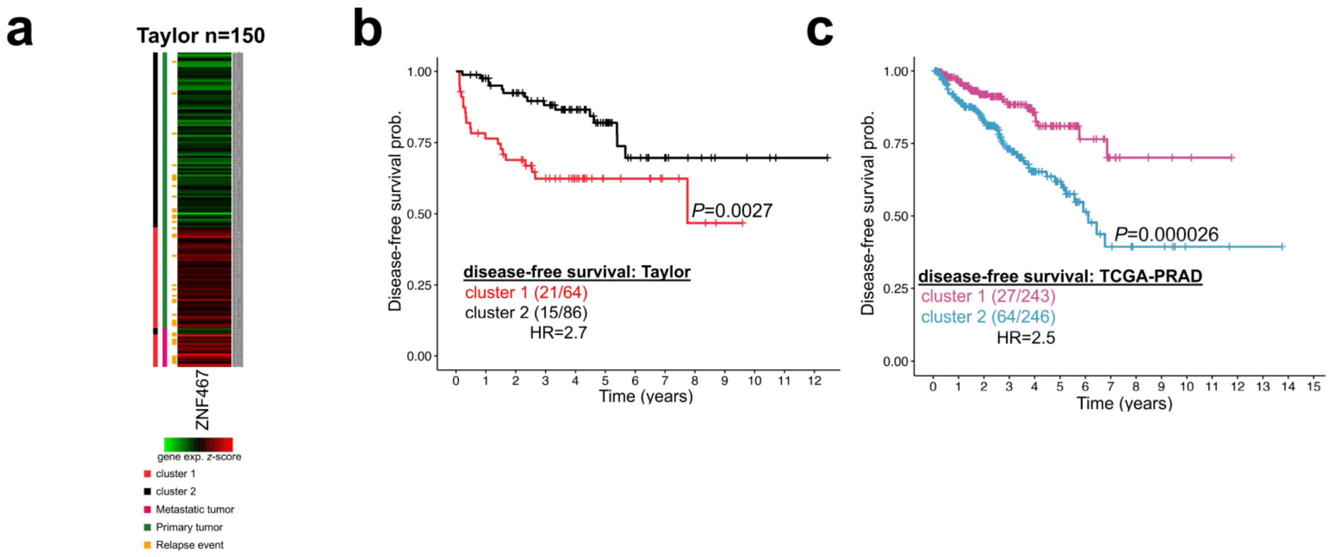

Supplementary Fig. S10. Zinc finger protein 467 (*ZNF467*), a gene induced by forced *GHSROS*-overexpression, is upregulated by metastatic tumors and associated with adverse relapse outcome. (a) Heat map of *ZNF467* expression in the Taylor cohort normalized to depict relative values within rows (samples) with high (red) and low expression (green). Vertical bars show patient grouping by *k*-means clustering (cluster 1, red; cluster 2, black), tumor type (primary, green; metastatic pink), and relapse status (relapse event, orange). **(b)** Kaplan-Meier analyses of *ZNF467* in the Taylor cohort. Patients were stratified by *k*-means clustering, as described in (a). **(c)** Kaplan-Meier analyses of *ZNF467* in the TCGA-PRAD cohort of 489 localized prostate tumors. Patients were stratified by *k*-means clustering (cluster 1, purple; cluster 2, turquoise).

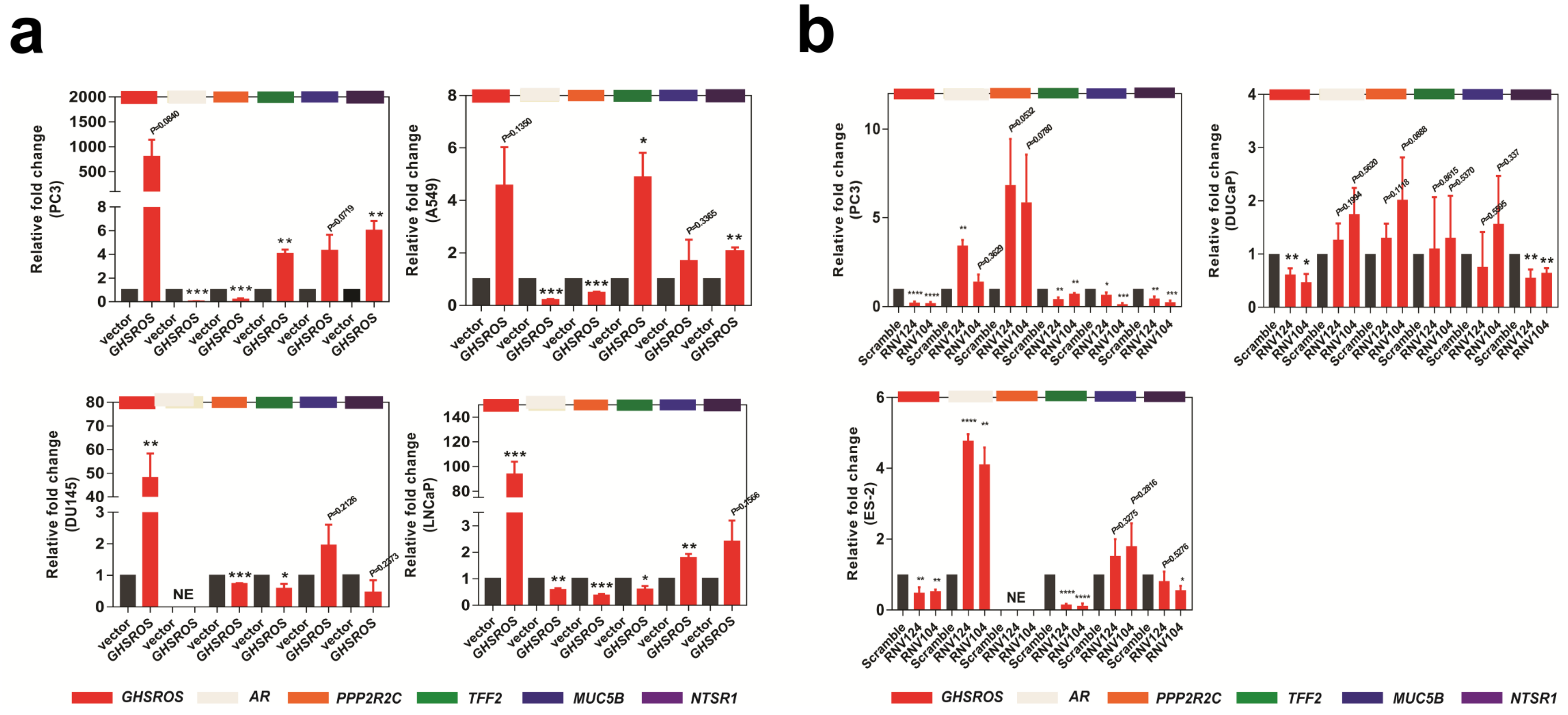

Supplementary Fig. S11. Effects of *GHSROS* perturbation in cultured cells assessed by qRT-PCR. (a) qRT-PCR validation of 5 genes regulated by *GHSROS*. Expression was normalized to the housekeeping gene RPL32. Results are relative to the respective vector control. Coloured bars indicate individual genes. Genes that were not expressed represented as no expression (NE). Mean ± s.e.m., n=3, *$P \leq 0.05$, **$P \leq 0.01$, ***$P \leq 0.001$, Student's t-test. **(b)** qRT-PCR validation of regulated genes following knockdown of *GHSROS* by transfection with LNA-ASOs for 48 hours. Expression was normalized to the housekeeping gene *RPL32*. Results are relative to the respective scrambled control. Annotated as in (a).

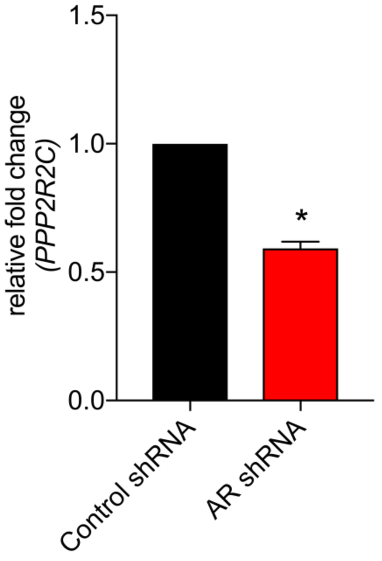

Supplementary Fig. S12. Effect of androgen receptor (AR) perturbation in LP50 prostate cancer cells on *PPP2R2C* expression. Assessed by microarray (NCBI GEO accession no. GSE22483). Mean ± s.e.m. n=2, *$Q \leq 0.05$, moderated t-test.

Supplementary Table S1. Correlation between *GHSROS* expression and clinicopathological parameters in OriGene TissueScan Prostate Cancer Tissue qPCR panels. Six samples were excluded due to missing clinical information. Relative *GHSROS* expression in tumors (T) stratified by clinical stage and Gleason score was compared to a normal prostate sample (N). *P*-values were calculated using the Mann-Whitney-Wilcoxon test. NA = not applicable, PD = other prostatic diseases.

**Clinicopathological parameters**	**Sample number (*n*)**	***P*-value**
**Age at diagnosis (mean ± SD)**	62.2 ± 7.80	
N/ T	24/88	0.1413
PD/ T	31/88	0.8001
N/ PD	24/31	0.0691
**Clinical stage**		
N (normal prostate)	24	
I	0	NA
II	47	0.311
III	33	0.0855
IV	3	0.0185
**Gleason score**		
N (normal prostate)	24	
2-6	15	0.0278
7	47	0.0751
8-10	25	0.00210

Supplementary Table S2. Correlation between *GHSROS* expression and clinicopathological parameters in the Andalusian Biobank prostate tissue cohort. Absolute levels of *GHSROS* expression in tumors were stratified by Gleason score and the number of metastatic tumor sites were compared to normal prostate (N). Tumors positive or negative for extraprostatic extension and perineural infiltration were compared to each other. *P*-values were calculated using the Mann-Whitney-Wilcoxon test. NA = not applicable.

**Clinicopathological parameters**	**Sample number (*n*)**	***P*-value**
**Age at diagnosis (mean ± SD)**	73.7 ± 9.81	
**Gleason score**		
N (normal prostate)	8	
7	6	0.00870
8	9	0.0240
9-10	13	0.0420
**Number of metastatic sites**		
N (normal prostate)	8	
primary prostate tumor: 0/localized	10	0.0420
primary prostate tumor: 1 metastatic site	6	0.0556
primary prostate tumor: ≥2 metastatic sites	12	0.0127
**Extraprostatic extension**		
-	16	0.379
+	11	
**Perineural infiltration**		
-	8	0.415
+	20	

Supplementary Table S3. Differentially expressed genes in PC3-GHSROS cells compared to empty vector control. Red: higher expression in PC3-GHSROS cells; Black: lower expression in PC3-GHSROS cells. Fold-changes are $\log_2$ transformed; *Q*-value denotes the false discovery rate (FDR; Benjamini-Hochberg)-adjusted *P*-value (cutoff ≤ 0.05).

Gene Symbol	**Gene Name**	**$\log_2$ Fold Change**	***P*-value**	***Q*-value**
AADACP1	arylacetamide deacetylase pseudogene 1	-1.9	8.2E-08	1.3E-06
AASS	aminoadipate-semialdehyde synthase	-2.4	2.5E-08	5.3E-07
AATK	apoptosis associated tyrosine kinase	1.8	5.1E-08	8.9E-07
ABCC3	ATP binding cassette subfamily C member 3	1.8	7.7E-13	7.1E-10
ABCG1	ATP binding cassette subfamily G member 1	2.1	1.7E-09	7.5E-08
ACHE	acetylcholinesterase (Cartwright blood group)	2.5	4.3E-10	3.0E-08
ACSS1	acyl-CoA synthetase short-chain family member 1	-1.9	7.4E-11	9.6E-09
ADAM23	ADAM metallopeptidase domain 23	-2.7	5.3E-10	3.5E-08
ADAM8	ADAM metallopeptidase domain 8	2.7	5.5E-14	1.5E-10
ADD2	adducin 2	-3.2	2.1E-09	8.8E-08
ADSSL1	adenylosuccinate synthase like 1	1.5	6.2E-08	1.0E-06
AFF2	AF4/FMR2 family member 2	-3.9	1.1E-11	3.0E-09
AGR2	anterior gradient 2, protein disulphide isomerase family member	2.1	3.9E-12	1.7E-09
AGTR1	angiotensin II receptor type 1	-2.1	3.6E-08	7.0E-07
AIF1L	allograft inflammatory factor 1 like	1.6	2.7E-08	5.6E-07
AMOT	angiomotin	-3.1	2.3E-11	4.7E-09
ANGPT1	angiopoietin 1	-2.8	1.0E-08	2.7E-07
ANGPTL4	angiopoietin like 4	1.6	2.0E-08	4.5E-07
ANK1	ankyrin 1	1.5	9.3E-06	5.5E-05
ANK2	ankyrin 2, neuronal	-3.1	2.4E-09	9.5E-08
ANOS1	anosmin 1	1.9	6.1E-09	1.8E-07
ANXA10	annexin A10	-2.3	1.1E-09	5.7E-08
APOBEC3G	apolipoprotein B mRNA editing enzyme catalytic subunit 3G	2.0	3.3E-08	6.5E-07
AR	androgen receptor	-3.6	1.4E-09	6.6E-08
ARHGAP44	Rho GTPase activating protein 44	-2.1	1.6E-08	3.8E-07
ARHGDIB	Rho GDP dissociation inhibitor beta	-3.4	5.3E-11	7.7E-09
ARHGEF16	Rho guanine nucleotide exchange factor 16	2.4	7.8E-10	4.6E-08
ARNT2	aryl hydrocarbon receptor nuclear translocator 2	-1.6	1.0E-07	1.5E-06
ASS1	argininosuccinate synthase 1	1.8	2.2E-08	4.8E-07
B3GALT5	beta-1,3-galactosyltransferase 5	-2.0	2.1E-06	1.7E-05

B3GALT5-AS1	B3GALT5 antisense RNA 1	-2.2	6.1E-09	1.8E-07
B3GNT7	UDP-GlcNAc:betaGal beta-1,3-N-acetylglucosaminyltransferase 7	1.6	4.2E-09	1.4E-07
B4GALNT1	beta-1,4-N-acetyl-galactosaminyltransferase 1	-1.8	8.8E-11	1.1E-08
BCAT1	branched chain amino acid transaminase 1	-3.9	1.5E-11	3.4E-09
BEX2	brain expressed X-linked 2	-1.8	4.2E-07	4.4E-06
BEX4	brain expressed X-linked 4	-1.8	1.1E-08	2.9E-07
BEX5	brain expressed X-linked 5	-1.7	8.1E-07	7.5E-06
BIK	BCL2 interacting killer	1.8	2.1E-06	1.6E-05
BLMH	bleomycin hydrolase	-1.8	6.6E-13	6.5E-10
BMP6	bone morphogenetic protein 6	-2.3	5.1E-09	1.6E-07
BTBD11	BTB domain containing 11	-3.1	5.7E-10	3.6E-08
BTG3	BTG family member 3	-2.0	1.1E-10	1.3E-08
C11orf45	chromosome 11 open reading frame 45	-1.8	2.0E-08	4.5E-07
C20orf166-AS1	C20orf166 antisense RNA 1	1.8	3.0E-07	3.5E-06
C9orf152	chromosome 9 open reading frame 152	1.8	5.3E-07	5.4E-06
CA9	carbonic anhydrase 9	2.5	3.1E-08	6.2E-07
CACNA2D2	calcium voltage-gated channel auxiliary subunit alpha2delta 2	-1.6	2.6E-08	5.5E-07
CADM4	cell adhesion molecule 4	2.3	8.8E-10	5.0E-08
CADPS2	calcium dependent secretion activator 2	-2.0	8.9E-12	2.6E-09
CALB1	calbindin 1	3.3	2.1E-10	1.9E-08
CAMK2N1	calcium/calmodulin dependent protein kinase II inhibitor 1	3.3	4.1E-12	1.7E-09
CAPN6	calpain 6	-2.5	3.3E-08	6.5E-07
CAV1	caveolin 1	1.6	5.2E-13	6.0E-10
CBLC	Cbl proto-oncogene C	1.8	2.8E-07	3.3E-06
CCBE1	collagen and calcium binding EGF domains 1	1.5	1.5E-08	3.7E-07
CCDC160	coiled-coil domain containing 160	-2.5	1.6E-08	3.8E-07
CCNB3	cyclin B3	-2.1	5.3E-08	9.2E-07
CD24	CD24 molecule	1.5	2.9E-12	1.5E-09
CD33	CD33 molecule	-2.0	5.7E-08	9.7E-07
CD70	CD70 molecule	-2.1	6.3E-07	6.2E-06
CDH1	cadherin 1	2.2	4.1E-08	7.8E-07
CDH12	cadherin 12	-1.6	8.8E-10	5.0E-08
CDH3	cadherin 3	2.0	1.5E-07	2.1E-06
CEACAM6	carcinoembryonic antigen related cell adhesion molecule 6	1.7	6.2E-07	6.1E-06
CEND1	cell cycle exit and neuronal differentiation 1	-2.0	2.4E-07	2.9E-06
CHD7	chromodomain helicase DNA binding protein 7	-1.6	1.1E-06	9.6E-06
CHRDL1	chordin-like 1	-3.0	5.4E-10	3.5E-08
CKB	creatine kinase B	1.8	2.6E-11	5.0E-09
CLDN7	claudin 7	2.1	1.4E-07	1.9E-06
CLMN	calmin (calponin-like, transmembrane)	-2.4	4.9E-09	1.6E-07

CNKSR2	connector enhancer of kinase suppressor of Ras 2	-2.4	9.2E-09	2.5E-07
CNTN1	contactin 1	-4.4	4.2E-11	6.6E-09
COBL	cordon-bleu WH2 repeat protein	-4.6	1.0E-11	2.9E-09
COL21A1	collagen type XXI alpha 1 chain	-1.9	1.2E-07	1.7E-06
COL5A1	collagen type V alpha 1	2.1	1.9E-09	8.2E-08
COX7B2	cytochrome c oxidase subunit 7B2	3.8	1.8E-09	7.6E-08
CPA6	carboxypeptidase A6	-2.3	1.5E-08	3.5E-07
CPEB1	cytoplasmic polyadenylation element binding protein 1	-2.5	5.4E-11	7.7E-09
CPZ	carboxypeptidase Z	1.6	1.7E-09	7.5E-08
CRABP1	cellular retinoic acid binding protein 1	4.1	1.1E-11	3.0E-09
CRABP2	cellular retinoic acid binding protein 2	2.4	1.1E-12	7.8E-10
CREB3L1	cAMP responsive element binding protein 3 like 1	4.0	2.4E-13	3.4E-10
CRIP1	cysteine rich protein 1	2.2	1.4E-10	1.5E-08
CRIP2	cysteine rich protein 2	2.3	9.6E-12	2.8E-09
CSMD2	CUB and Sushi multiple domains 2	-2.5	7.9E-09	2.2E-07
CST1	cystatin SN	-3.3	1.3E-10	1.4E-08
CST4	cystatin S	-2.6	1.8E-09	7.6E-08
CXADR	coxsackie virus and adenovirus receptor	-2.8	4.0E-11	6.5E-09
CXADRP2	coxsackie virus and adenovirus receptor pseudogene 2	-1.8	3.5E-07	3.9E-06
CXCL5	C-X-C motif chemokine ligand 5	1.5	1.0E-06	9.0E-06
CXorf57	chromosome X open reading frame 57	-3.0	4.7E-10	3.2E-08
CYP4F35P	cytochrome P450 family 4 subfamily F member 35, pseudogene	-1.5	1.4E-07	2.0E-06
CYP4V2	cytochrome P450 family 4 subfamily V member 2	-2.6	2.3E-09	9.1E-08
CYP7B1	cytochrome P450 family 7 subfamily B member 1	-2.3	2.2E-08	4.8E-07
DEPTOR	DEP domain containing MTOR-interacting protein	-1.7	6.7E-08	1.1E-06
DGKG	diacylglycerol kinase gamma	-2.7	1.0E-10	1.2E-08
DIRAS1	DIRAS family GTPase 1	2.2	2.8E-10	2.2E-08
DLX3	distal-less homeobox 3	-1.6	1.7E-06	1.4E-05
DMD	dystrophin	-3.0	4.0E-10	2.8E-08
DNAH5	dynein axonemal heavy chain 5	-1.7	3.3E-09	1.2E-07
DNAJA4	DnaJ heat shock protein family (Hsp40) member A4	-2.0	1.8E-08	4.1E-07
DNAJC6	DnaJ heat shock protein family (Hsp40) member C6	-2.4	4.6E-10	3.1E-08
DOCK3	dedicator of cytokinesis 3	-1.5	9.4E-09	2.6E-07
DPY19L2P1	DPY19L2 pseudogene 1	-1.9	1.2E-06	1.1E-05
DTX4	deltex 4, E3 ubiquitin ligase	-1.8	1.1E-08	2.8E-07
DYSF	dysferlin	-2.0	5.9E-11	8.3E-09
EDA	ectodysplasin A	-3.0	6.2E-09	1.8E-07
EGF	epidermal growth factor	-2.5	9.1E-10	5.1E-08
EGLN3	egl-9 family hypoxia inducible factor 3	-1.8	1.3E-07	1.8E-06
EHD2	EH domain containing 2	2.3	9.7E-09	2.6E-07
EHF	ETS homologous factor	1.9	8.5E-09	2.4E-07
ENTPD3	ectonucleoside triphosphate diphosphohydrolase 3	-1.8	1.1E-08	2.8E-07

EOMES	eomesodermin	-2.1	1.2E-09	6.1E-08
EPHB2	EPH receptor B2	-1.5	2.6E-10	2.1E-08
EPHB6	EPH receptor B6	1.7	1.4E-10	1.5E-08
ESM1	endothelial cell specific molecule 1	-1.8	4.3E-11	6.7E-09
EYA1	EYA transcriptional coactivator and phosphatase 1	-3.5	1.5E-10	1.6E-08
F2RL2	coagulation factor II thrombin receptor like 2	-1.6	2.0E-07	2.6E-06
FAM110C	family with sequence similarity 110 member C	-3.0	1.6E-10	1.6E-08
FAM131B	family with sequence similarity 131 member B	1.5	1.7E-11	3.6E-09
FAM134B	family with sequence similarity 134 member B	-1.5	6.3E-08	1.0E-06
FAM20A	family with sequence similarity 20 member A	2.0	1.3E-07	1.9E-06
FAM50B	family with sequence similarity 50 member B	1.7	8.5E-08	1.3E-06
FAM89A	family with sequence similarity 89 member A	-1.6	5.1E-08	8.9E-07
FBP1	fructose-bisphosphatase 1	2.5	3.1E-11	5.6E-09
FBXL16	F-box and leucine rich repeat protein 16	2.0	1.4E-08	3.5E-07
FBXL7	F-box and leucine rich repeat protein 7	-1.7	1.6E-08	3.7E-07
FCGBP	Fc fragment of IgG binding protein	-3.1	1.5E-10	1.6E-08
FEZF1-AS1	FEZF1 antisense RNA 1	-2.0	1.1E-06	9.8E-06
FGF13	fibroblast growth factor 13	-1.8	1.7E-08	4.0E-07
FNDC4	fibronectin type III domain containing 4	2.0	4.2E-08	7.9E-07
FOXL1	forkhead box L1	1.6	4.1E-10	2.9E-08
FOXRED2	FAD dependent oxidoreductase domain containing 2	3.6	2.3E-12	1.5E-09
FRMD4B	FERM domain containing 4B	-1.9	5.5E-09	1.7E-07
GAL	galanin and GMAP prepropeptide	-3.2	1.5E-09	6.7E-08
GALNT12	polypeptide N-acetylgalactosaminyltransferase 12	2.1	9.9E-10	5.3E-08
GALNT14	polypeptide N-acetylgalactosaminyltransferase 14	-1.6	1.3E-08	3.3E-07
GALNT16	polypeptide N-acetylgalactosaminyltransferase 16	-1.7	1.2E-06	1.0E-05
GAS6	growth arrest specific 6	1.6	2.1E-09	8.8E-08
GBP7	guanylate binding protein 7	-1.5	2.9E-06	2.1E-05
GCNT1	glucosaminyl (N-acetyl) transferase 1, core 2	-1.6	1.4E-07	1.9E-06
GCNT3	glucosaminyl (N-acetyl) transferase 3, mucin type	-2.4	6.8E-09	2.0E-07
GDF15	growth differentiation factor 15	1.5	1.5E-08	3.6E-07
GHSROS	Growth Hormone Secretagogue Receptor Opposite Strand	5.3	3.4E-13	4.3E-10
GJB3	gap junction protein beta 3	1.7	6.0E-10	3.8E-08
GNAI1	G protein subunit alpha i1	1.5	1.6E-10	1.6E-08
GPR153	G protein-coupled receptor 153	1.6	5.7E-13	6.1E-10
GPR63	G protein-coupled receptor 63	-2.3	2.5E-09	9.7E-08
GRID2	glutamate ionotropic receptor delta type subunit 2	1.7	1.5E-07	2.1E-06
GRIN2D	glutamate ionotropic receptor NMDA type subunit 2D	2.0	1.6E-08	3.7E-07
GRTP1	growth hormone regulated TBC protein 1	2.1	6.0E-08	1.0E-06
GUCA1B	guanylate cyclase activator 1B	1.5	6.7E-07	6.5E-06
HEPH	hephaestin	-2.4	2.4E-09	9.3E-08

HR	hair growth associated	2.2	5.3E-09	1.7E-07
HRASLS	HRAS like suppressor	-1.9	2.7E-07	3.2E-06
HSPA12A	heat shock protein family A (Hsp70) member 12A	-3.7	2.7E-11	5.1E-09
HSPB8	heat shock protein family B (small) member 8	-3.5	9.9E-10	5.3E-08
HTR7	5-hydroxytryptamine receptor 7	-2.0	2.4E-07	2.9E-06
HTRA3	HtrA serine peptidase 3	-1.8	1.5E-08	3.6E-07
IFI16	interferon gamma inducible protein 16	-2.2	9.4E-13	7.5E-10
IFITM1	interferon induced transmembrane protein 1	1.6	3.6E-06	2.5E-05
IGFBP5	insulin like growth factor binding protein 5	1.6	2.4E-12	1.5E-09
IGFBP6	insulin like growth factor binding protein 6	1.9	3.8E-11	6.3E-09
IL13RA2	interleukin 13 receptor subunit alpha 2	-3.0	6.4E-10	4.0E-08
ITGB2	integrin subunit beta 2	2.2	6.1E-11	8.4E-09
ITGB4	integrin subunit beta 4	1.9	9.2E-13	7.5E-10
ITM2A	integral membrane protein 2A	-2.7	7.3E-10	4.4E-08
JAM3	junctional adhesion molecule 3	-1.8	1.3E-08	3.3E-07
JUP	junction plakoglobin	1.5	5.9E-12	2.0E-09
KCNJ12	potassium voltage-gated channel subfamily J member 12	-2.2	4.3E-09	1.4E-07
KCNJ3	potassium voltage-gated channel subfamily J member 3	1.5	2.4E-05	1.2E-04
KCNN3	potassium calcium-activated channel subfamily N member 3	-2.3	5.9E-07	5.9E-06
KIAA0319	KIAA0319	-3.6	4.6E-11	6.9E-09
KIAA1210	KIAA1210	-1.7	2.9E-07	3.4E-06
KIAA1211	KIAA1211	-3.8	2.7E-12	1.5E-09
KIAA1644	KIAA1644	2.0	2.6E-07	3.1E-06
KIF26A	kinesin family member 26A	-1.7	4.5E-08	8.3E-07
KIF5C	kinesin family member 5C	-2.7	1.6E-11	3.5E-09
KLF9	Kruppel like factor 9	-3.3	2.5E-10	2.1E-08
KRT6B	keratin 6B	-2.2	1.7E-08	3.9E-07
KRT7	keratin 7	2.6	1.9E-14	6.7E-11
KRT75	keratin 75	-5.5	3.5E-16	2.5E-12
KRT81	keratin 81	1.5	3.1E-12	1.5E-09
LAMA1	laminin subunit alpha 1	-2.1	1.6E-08	3.8E-07
LAMC3	laminin subunit gamma 3	-1.7	2.1E-09	8.5E-08
LARGE2	LARGE xylosyl- and glucuronyltransferase 2	1.8	4.3E-08	8.0E-07
LCK	LCK proto-oncogene, Src family tyrosine kinase	1.5	2.6E-06	2.0E-05
LCP1	lymphocyte cytosolic protein 1	-2.3	3.4E-12	1.6E-09
LEF1	lymphoid enhancer binding factor 1	-1.5	6.7E-07	6.5E-06
LGR5	leucine rich repeat containing G protein-coupled receptor 5	-1.7	3.9E-09	1.3E-07
LIMCH1	LIM and calponin homology domains 1	2.2	6.1E-10	3.8E-08
LIMS2	LIM zinc finger domain containing 2	1.7	3.1E-09	1.2E-07
LINC00346	long intergenic non-protein coding RNA 346	1.5	8.0E-07	7.4E-06
LINC01133	long intergenic non-protein coding RNA 1133	1.5	4.1E-06	2.8E-05
LITAF	lipopolysaccharide induced TNF factor	3.1	1.1E-10	1.3E-08

LOC100506123	uncharacterized LOC100506123	-1.6	1.4E-09	6.7E-08
LOC101927870	uncharacterized LOC101927870	1.5	5.2E-09	1.6E-07
LOXL4	lysyl oxidase like 4	1.7	4.4E-12	1.8E-09
LRCH2	leucine rich repeats and calponin homology domain containing 2	-2.4	3.7E-09	1.3E-07
LRIG1	leucine rich repeats and immunoglobulin like domains 1	-2.0	1.6E-08	3.7E-07
LRP4	LDL receptor related protein 4	-1.5	1.8E-05	9.3E-05
LRRN2	leucine rich repeat neuronal 2	-1.5	8.3E-07	7.7E-06
MAGEH1	MAGE family member H1	-1.9	1.1E-08	2.8E-07
MAP2	microtubule associated protein 2	-1.5	3.0E-08	6.0E-07
MAP3K15	mitogen-activated protein kinase kinase kinase 15	-1.5	8.5E-09	2.3E-07
MARCH3	membrane associated ring-CH-type finger 3	1.5	2.2E-06	1.7E-05
MARK1	microtubule affinity regulating kinase 1	-2.9	2.7E-10	2.2E-08
MCOLN3	mucolipin 3	-1.8	2.1E-06	1.7E-05
MCTP2	multiple C2 domains, transmembrane 2	-3.0	3.1E-10	2.3E-08
MEGF6	multiple EGF like domains 6	2.0	4.0E-12	1.7E-09
MEST	mesoderm specific transcript	1.7	7.0E-09	2.0E-07
MFAP3L	microfibrillar associated protein 3 like	-2.5	1.3E-09	6.3E-08
MIR31HG	MIR31 host gene	1.7	2.4E-09	9.3E-08
MISP	mitotic spindle positioning	1.7	1.0E-10	1.2E-08
MLLT11	myeloid/lymphoid or mixed-lineage leukemia; translocated to, 11	-3.2	3.1E-16	2.5E-12
MMRN1	multimerin 1	-1.8	1.1E-08	2.9E-07
MN1	meningioma (disrupted in balanced translocation) 1	-2.6	1.2E-10	1.3E-08
MSX2	msh homeobox 2	-2.1	1.3E-08	3.3E-07
MTSS1	metastasis suppressor 1	-1.5	4.2E-07	4.4E-06
MUC2	mucin 2, oligomeric mucus/gel-forming	2.0	1.3E-08	3.3E-07
MUC3A	mucin 3A, cell surface associated	3.7	7.5E-12	2.4E-09
MUC5B	mucin 5B, oligomeric mucus/gel-forming	2.8	2.3E-09	9.2E-08
MUM1L1	MUM1 like 1	-3.5	1.1E-09	5.6E-08
MYT1	myelin transcription factor 1	-1.6	4.8E-06	3.2E-05
NACAD	NAC alpha domain containing	2.9	4.0E-09	1.4E-07
NAP1L2	nucleosome assembly protein 1 like 2	-1.7	1.4E-11	3.3E-09
NAP1L6	nucleosome assembly protein 1 like 6	-1.8	2.0E-06	1.6E-05
NAV3	neuron navigator 3	-1.6	4.3E-07	4.6E-06
NBEAP1	neurobeachin pseudogene 1	-1.8	6.9E-07	6.6E-06
NCR3LG1	natural killer cell cytotoxicity receptor 3 ligand 1	-1.7	8.9E-06	5.3E-05
NEK3	NIMA related kinase 3	2.0	1.4E-08	3.4E-07
NELL2	neural EGFL like 2	-4.3	2.6E-12	1.5E-09
NEO1	neogenin 1	-1.5	3.2E-06	2.3E-05
NFASC	neurofascin	-2.3	7.1E-11	9.5E-09
NLGN1	neuroligin 1	-1.7	1.9E-06	1.5E-05
NLRC5	NLR family CARD domain containing 5	-1.5	3.9E-07	4.2E-06
NOG	noggin	-1.5	1.3E-09	6.4E-08
NPR3	natriuretic peptide receptor 3	1.6	1.2E-07	1.7E-06

NPY1R	neuropeptide Y receptor Y1	1.9	3.0E-11	5.4E-09
NRXN3	neurexin 3	-1.9	1.2E-07	1.7E-06
NTSR1	neurotensin receptor 1 (high affinity)	4.0	1.4E-14	6.3E-11
NUDT10	nudix hydrolase 10	-2.6	9.7E-10	5.3E-08
NUDT11	nudix hydrolase 11	-4.9	2.4E-13	3.4E-10
OASL	2'-5'-oligoadenylate synthetase like	-1.6	1.8E-10	1.6E-08
OCLN	occludin	1.7	4.0E-09	1.4E-07
OPN3	opsin 3	1.5	5.9E-09	1.8E-07
OSBP2	oxysterol binding protein 2	1.8	1.5E-12	1.0E-09
OXTR	oxytocin receptor	1.7	7.0E-12	2.3E-09
PALD1	phosphatase domain containing, paladin 1	-2.2	2.0E-08	4.5E-07
PALM2	paralemmin 2	-2.1	5.0E-07	5.2E-06
PAQR8	progestin and adipoQ receptor family member 8	1.7	3.1E-09	1.1E-07
PARM1	prostate androgen-regulated mucin-like protein 1	-4.4	3.2E-12	1.5E-09
PARP8	poly(ADP-ribose) polymerase family member 8	-1.9	4.0E-09	1.4E-07
PCDH19	protocadherin 19	-1.7	2.0E-06	1.6E-05
PCDHGB2	protocadherin gamma subfamily B, 2	1.7	3.4E-07	3.8E-06
PCSK5	proprotein convertase subtilisin/kexin type 5	-1.9	2.4E-07	2.9E-06
PCSK9	proprotein convertase subtilisin/kexin type 9	1.7	4.8E-08	8.7E-07
PDGFA	platelet derived growth factor subunit A	1.6	1.9E-08	4.3E-07
PDLIM2	PDZ and LIM domain 2	1.9	8.0E-09	2.3E-07
PELI2	pellino E3 ubiquitin protein ligase family member 2	-1.6	2.3E-06	1.8E-05
PI3	peptidase inhibitor 3	1.7	5.8E-10	3.7E-08
PIANP	PILR alpha associated neural protein	1.6	8.1E-07	7.5E-06
PLAT	plasminogen activator, tissue type	1.8	1.9E-13	3.4E-10
PLBD1	phospholipase B domain containing 1	-1.5	4.1E-06	2.8E-05
PLCH2	phospholipase C eta 2	1.7	8.4E-10	4.8E-08
PLD5	phospholipase D family member 5	-2.1	4.5E-08	8.2E-07
PLEKHG6	pleckstrin homology and RhoGEF domain containing G6	1.5	3.3E-06	2.3E-05
PLLP	plasmolipin	1.5	9.7E-07	8.7E-06
PLXNA4	plexin A4	2.6	1.9E-07	2.4E-06
POF1B	premature ovarian failure, 1B	-1.5	1.6E-06	1.3E-05
POU3F2	POU class 3 homeobox 2	-3.6	2.7E-11	5.1E-09
PPL	periplakin	2.2	1.7E-10	1.6E-08
PPP1R1B	protein phosphatase 1 regulatory inhibitor subunit 1B	3.2	5.9E-10	3.7E-08
PPP1R3G	protein phosphatase 1 regulatory subunit 3G	2.3	4.3E-09	1.4E-07
PPP2R2C	protein phosphatase 2 regulatory subunit Bgamma	-4.9	1.9E-13	3.4E-10
PREX2	phosphatidylinositol-3,4,5-trisphosphate dependent Rac exchange factor 2	-1.6	8.3E-07	7.7E-06
PRKXP1	protein kinase, X-linked, pseudogene 1	-1.5	6.6E-06	4.2E-05
PRR15	proline rich 15	1.8	4.8E-08	8.6E-07
PTGER2	prostaglandin E receptor 2	1.9	1.5E-09	6.9E-08

PTGFRN	prostaglandin F2 receptor inhibitor	-1.6	4.6E-10	3.2E-08
PTGS2	prostaglandin-endoperoxide synthase 2	1.7	4.9E-07	5.1E-06
PTPN20	protein tyrosine phosphatase, non-receptor type 20	1.8	5.1E-08	9.0E-07
PTPRG	protein tyrosine phosphatase, receptor type G	-2.5	5.3E-09	1.7E-07
RAB39B	RAB39B, member RAS oncogene family	-2.1	9.3E-08	1.4E-06
RASD1	ras related dexamethasone induced 1	2.4	4.9E-09	1.6E-07
RASEF	RAS and EF-hand domain containing	-1.8	3.6E-07	4.0E-06
RASL11A	RAS like family 11 member A	1.9	6.8E-11	9.2E-09
RBM11	RNA binding motif protein 11	-3.2	1.4E-10	1.5E-08
RBP4	retinol binding protein 4	1.6	1.3E-11	3.2E-09
RCOR2	REST corepressor 2	1.6	1.3E-06	1.1E-05
REG4	regenerating family member 4	2.2	5.1E-09	1.6E-07
RFTN1	raftlin, lipid raft linker 1	-1.8	6.6E-09	1.9E-07
RGAG4	retrotransposon gag domain containing 4	-1.6	5.0E-06	3.3E-05
RGCC	regulator of cell cycle	1.7	3.3E-06	2.3E-05
RHOU	ras homolog family member U	1.7	1.5E-07	2.0E-06
RIMKLB	ribosomal modification protein rimK-like family member B	-1.6	1.5E-08	3.6E-07
RNASEL	ribonuclease L	-2.4	1.4E-09	6.6E-08
RNF128	ring finger protein 128, E3 ubiquitin protein ligase	-3.8	1.5E-11	3.5E-09
RNF152	ring finger protein 152	-1.5	2.7E-06	2.0E-05
ROBO4	roundabout guidance receptor 4	-2.1	2.0E-11	4.1E-09
RPS6KA2	ribosomal protein S6 kinase A2	1.5	6.3E-08	1.0E-06
RRAGD	Ras related GTP binding D	-3.0	8.0E-11	1.0E-08
RUNDC3B	RUN domain containing 3B	-1.8	2.8E-06	2.0E-05
RYR2	ryanodine receptor 2	-2.9	3.2E-09	1.2E-07
S100A2	S100 calcium binding protein A2	2.1	5.4E-11	7.7E-09
S100A9	S100 calcium binding protein A9	-1.5	8.9E-06	5.3E-05
S100P	S100 calcium binding protein P	1.9	9.6E-07	8.6E-06
SCIN	scinderin	1.5	7.2E-07	6.8E-06
SCN3A	sodium voltage-gated channel alpha subunit 3	-1.9	2.4E-08	5.1E-07
SEMA3B	semaphorin 3B	1.9	3.4E-10	2.5E-08
SEMA6B	semaphorin 6B	2.3	5.3E-10	3.5E-08
SERPINA1	serpin family A member 1	-2.1	3.2E-08	6.4E-07
SERPINB2	serpin family B member 2	-2.9	2.4E-11	4.7E-09
SERTAD4	SERTA domain containing 4	-1.6	2.3E-06	1.7E-05
SESN3	sestrin 3	-2.6	5.4E-10	3.5E-08
SGK494	uncharacterized serine/threonine-protein kinase SgK494	-1.5	3.1E-08	6.2E-07
SH2D1B	SH2 domain containing 1B	-2.2	1.5E-08	3.5E-07
SIRPB1	signal regulatory protein beta 1	-2.3	6.3E-11	8.7E-09
SIRPB2	signal regulatory protein beta 2	-1.6	3.2E-06	2.3E-05
SLC16A9	solute carrier family 16 member 9	1.7	4.0E-11	6.5E-09
SLC1A3	solute carrier family 1 member 3	-3.7	4.2E-11	6.6E-09
SLC25A25-AS1	SLC25A25 antisense RNA 1	-1.5	3.2E-08	6.3E-07
SLC26A10	solute carrier family 26 member 10	-1.8	1.6E-10	1.6E-08
SLC26A5	solute carrier family 26 member 5	1.5	1.4E-05	7.6E-05
SLC29A4	solute carrier family 29 member 4	1.6	2.6E-08	5.4E-07

SLC38A11	solute carrier family 38 member 11	1.7	2.7E-07	3.2E-06
SLC44A5	solute carrier family 44 member 5	-2.1	6.3E-08	1.1E-06
SLC47A1	solute carrier family 47 member 1	1.8	2.6E-07	3.1E-06
SLC6A11	solute carrier family 6 member 11	-2.3	4.9E-08	8.8E-07
SLC7A8	solute carrier family 7 member 8	-3.4	4.0E-10	2.8E-08
SLC9A2	solute carrier family 9 member A2	-3.6	2.1E-11	4.2E-09
SLFN13	schlafen family member 13	-2.3	2.9E-10	2.2E-08
SLITRK4	SLIT and NTRK like family member 4	-1.8	1.7E-09	7.5E-08
SOD3	superoxide dismutase 3, extracellular	1.7	7.5E-09	2.1E-07
SPDEF	SAM pointed domain containing ETS transcription factor	1.5	2.3E-10	2.0E-08
SPOCK3	sparc/osteonectin, cwcv and kazal-like domains proteoglycan (testican) 3	1.5	7.9E-08	1.3E-06
SPTB	spectrin beta, erythrocytic	-1.7	1.0E-09	5.5E-08
SRPX	sushi repeat containing protein, X-linked	-1.5	1.2E-09	6.1E-08
ST6GAL1	ST6 beta-galactoside alpha-2,6-sialyltransferase 1	-3.8	2.8E-12	1.5E-09
ST6GAL2	ST6 beta-galactoside alpha-2,6-sialyltransferase 2	2.0	9.2E-09	2.5E-07
ST6GALNAC2	ST6 N-acetylgalactosaminide alpha-2,6-sialyltransferase 2	2.4	1.6E-09	7.2E-08
STARD8	StAR related lipid transfer domain containing 8	-1.8	1.7E-07	2.2E-06
STAT6	signal transducer and activator of transcription 6	1.9	1.3E-11	3.2E-09
STC1	stanniocalcin 1	-1.5	4.5E-08	8.3E-07
STMN3	stathmin 3	1.5	3.8E-08	7.3E-07
STOX2	storkhead box 2	-2.6	5.3E-09	1.7E-07
SUSD3	sushi domain containing 3	1.8	1.9E-07	2.5E-06
SYT14	synaptotagmin 14	-2.2	1.0E-07	1.5E-06
TACSTD2	tumor-associated calcium signal transducer 2	-2.0	3.0E-09	1.1E-07
TAGLN3	transgelin 3	-1.5	5.2E-09	1.6E-07
TBC1D30	TBC1 domain family member 30	-1.8	7.1E-08	1.2E-06
TCN1	transcobalamin 1	-5.4	1.7E-13	3.4E-10
TDO2	tryptophan 2,3-dioxygenase	-2.0	1.3E-06	1.1E-05
TENM1	teneurin transmembrane protein 1	-1.8	7.8E-09	2.2E-07
TFF1	trefoil factor 1	3.6	5.2E-12	1.9E-09
TFF2	trefoil factor 2	3.4	3.4E-10	2.5E-08
TGFBI	transforming growth factor beta induced	-1.6	2.2E-07	2.7E-06
THBS1	thrombospondin 1	1.5	5.5E-09	1.7E-07
TLR3	toll like receptor 3	-1.9	2.1E-07	2.6E-06
TMC6	transmembrane channel like 6	1.7	2.4E-09	9.3E-08
TMEM108-AS1	TMEM108 antisense RNA 1	-3.1	9.8E-11	1.2E-08
TMEM229B	transmembrane protein 229B	-1.5	8.4E-08	1.3E-06
TMEM27	transmembrane protein 27	-1.7	4.4E-11	6.8E-09
TMEM74	transmembrane protein 74	-1.8	1.7E-07	2.2E-06
TMOD2	tropomodulin 2	-2.3	3.0E-10	2.3E-08
TMPRSS15	transmembrane protease, serine 15	2.4	2.8E-08	5.7E-07
TMPRSS3	transmembrane protease, serine 3	2.1	3.3E-07	3.8E-06
TMX4	thioredoxin related transmembrane protein 4	-1.7	8.7E-12	2.6E-09

TNFRSF11B	tumor necrosis factor receptor superfamily member 11b	-1.9	8.9E-08	1.4E-06
TNFRSF14	tumor necrosis factor receptor superfamily member 14	1.7	1.0E-08	2.7E-07
TNFSF9	tumor necrosis factor superfamily member 9	-1.5	1.5E-07	2.0E-06
TNXB	tenascin XB	2.6	2.8E-10	2.2E-08
TP53I11	tumor protein p53 inducible protein 11	2.5	1.0E-08	2.7E-07
TPTE	transmembrane phosphatase with tensin homology	-2.1	2.4E-08	5.1E-07
TSPEAR	thrombospondin type laminin G domain and EAR repeats	1.9	3.8E-08	7.2E-07
TSPOAP1	TSPO associated protein 1	1.7	4.7E-07	4.9E-06
TTC3P1	tetratricopeptide repeat domain 3 pseudogene 1	-1.8	9.6E-08	1.5E-06
TTC9	tetratricopeptide repeat domain 9	1.6	6.7E-07	6.5E-06
UBE2QL1	ubiquitin conjugating enzyme E2 Q family like 1	-2.4	1.3E-11	3.2E-09
UNC80	unc-80 homolog, NALCN activator	-3.6	3.0E-11	5.4E-09
VASH1	vasohibin 1	-1.6	2.8E-10	2.2E-08
VAV3	vav guanine nucleotide exchange factor 3	-1.6	2.5E-07	3.0E-06
VSTM2L	V-set and transmembrane domain containing 2 like	2.0	1.2E-08	3.1E-07
VWA5A	von Willebrand factor A domain containing 5A	-3.4	1.5E-10	1.6E-08
WDR72	WD repeat domain 72	-3.2	3.7E-11	6.2E-09
WNT2B	Wnt family member 2B	-1.5	2.1E-06	1.7E-05
YPEL2	yippee like 2	-1.5	1.7E-07	2.3E-06
ZFPM2	zinc finger protein, FOG family member 2	-3.8	5.1E-12	1.9E-09
ZG16B	zymogen granule protein 16B	1.6	8.2E-08	1.3E-06
ZNF334	zinc finger protein 334	3.1	4.5E-09	1.5E-07
ZNF415	zinc finger protein 415	2.5	2.0E-10	1.8E-08
ZNF43	zinc finger protein 43	2.0	2.5E-07	3.0E-06
ZNF467	zinc finger protein 467	3.5	1.7E-10	1.6E-08
ZNF470	zinc finger protein 470	2.5	5.3E-09	1.7E-07
ZNF521	zinc finger protein 521	-1.5	7.8E-07	7.3E-06
ZNF585B	zinc finger protein 585B	-2.3	2.9E-08	5.9E-07
ZNF607	zinc finger protein 607	2.4	3.0E-09	1.1E-07
ZNF702P	zinc finger protein 702, pseudogene	2.2	3.2E-09	1.2E-07
ZNF818P	zinc finger protein 818, pseudogene	2.0	1.5E-07	2.0E-06
ZNF85	zinc finger protein 85	3.9	1.6E-11	3.5E-09

Supplementary Table S4. Enrichment for GO terms in the category 'biological process' for genes upregulated in PC3-GHSROS cells (compared to empty-vector control). $P \leq 0.01$, Fisher's exact test.

GO term	Description	Count	%	Genes	Fold Enrichment	Fisher Exact P-value
GO:0010669	epithelial structure maintenance	4	2.6	*MUC2, RBP4, MUC3A, TFF1*	70	1.1E-07
GO:0030277	maintenance of gastrointestinal epithelium	4	2.6	*MUC2, RBP4, MUC3A, TFF1*	70	1.1E-07
GO:0070482	response to oxygen levels	9	5.9	*PLAT, CAV1, CA9, PDGFA, OXTR, CD24, THBS1, SOD3, ANGPTL4*	7	7.5E-06
GO:0009725	response to hormone stimulus	13	8.5	*RBP4, CAV1, PTGS2, PDGFA, FBP1, OXTR, NPY1R, ABCG1, CA9, PCSK9, CD24, TFF1, THBS1*	4	4.4E-05
GO:0001666	response to hypoxia	8	5.2	*PLAT, CAV1, CA9, PDGFA, CD24, THBS1, SOD3, ANGPTL4*	6	4.0E-05
GO:0022600	digestive system process	5	3.3	*MUC2, RBP4, MUC3A, OXTR, TFF1*	16	1.6E-05
GO:0009719	response to endogenous stimulus	13	8.5	*RBP4, CAV1, PTGS2, PDGFA, FBP1, OXTR, NPY1R, ABCG1, CA9, PCSK9, CD24, TFF1, THBS1*	3	1.2E-04
GO:0048545	response to steroid hormone stimulus	9	5.9	*CAV1, PTGS2, CA9, PDGFA, OXTR, TFF1, NPY1R, CD24, THBS1*	5	8.6E-05
GO:0043627	response to estrogen stimulus	7	4.6		7	6.1E-05
GO:0008285	negative regulation of cell proliferation	12	7.8	*MUC2, RBP4, CAV1, TP53I11, IFITM1, PTGS2, IGFBP6, SCIN, TNFRSF14, CD24, THBS1, IGFBP5*	4	1.6E-04
GO:0051241	negative regulation of multicellular organismal process	8	5.2	*RBP4, CAV1, ACHE, PTGS2, PDGFA, PCSK9, CD24, THBS1*	5	1.6E-04
GO:0042493	response to drug	9	5.9	*CAV1, PTGS2, CA9, PDGFA, LCK, OXTR, CDH1, CDH3, SLC47A1*	4	2.1E-04
GO:0032355	response to estradiol stimulus	5	3.3	*PTGS2, PDGFA, OXTR, TFF1, NPY1R*	10	1.5E-04
GO:0007586	digestion	6	3.9	*MUC2, RBP4, MUC3A, TFF2, OXTR, TFF1*	7	2.2E-04
GO:0042127	regulation of cell proliferation	17	11.1	*MUC2, RBP4, CAV1, PTGER2, TP53I11, PTGS2, IFITM1, CXCL5, PDGFA, CRIP2, IGFBP6, TNFRSF14, STAT6, SCIN, CD24, THBS1, IGFBP5*	2	1.2E-03

GO:0010033	response to organic substance	16	10.5	*RBP4, CAV1, PTGS2, PDGFA, FBP1, OXTR, CDH1, NPY1R, ABCG1, STAT6, CA9, PCSK9, CREB3L1, CD24, TFF1, THBS1*	2	1.3E-03
GO:0031644	regulation of neurological system process	7	4.6	*PLAT, ACHE, S100P, PTGS2, GRIN2D, OXTR, CALB1*	5	6.2E-04
GO:0050730	regulation of peptidyl-tyrosine phosphorylation	5	3.3	*CAV1, PDGFA, TNFRSF14, ITGB2, CD24*	8	4.5E-04
GO:0007267	cell-cell signaling	14	9.2	*PLAT, GUCA1B, ACHE, CXCL5, PDGFA, OXTR, ITGB2, NTSR1, GRIN2D, GRID2, CEACAM6, SEMA3B, CD24, GDF15*	2	1.6E-03
GO:0042632	cholesterol homeostasis	4	2.6	*CAV1, PCSK9, CD24, ABCG1*	11	4.4E-04
GO:0055092	sterol homeostasis	4	2.6	*CAV1, PCSK9, CD24, ABCG1*	11	4.4E-04
GO:0007155	cell adhesion	15	9.8	*CLDN7, ACHE, CADM4, TNXB, ITGB4, ITGB2, CDH1, CDH3, PCDHGB2, COL5A1, JUP, CD24, ADAM8, THBS1, MUC5B*	2	2.5E-03
GO:0022610	biological adhesion	15	9.8	*CLDN7, ACHE, CADM4, TNXB, ITGB4, ITGB2, CDH1, CDH3, PCDHGB2, COL5A1, JUP, CD24, ADAM8, THBS1, MUC5B*	2	2.6E-03
GO:0045907	positive regulation of vasoconstriction	3	2.0	*CAV1, PTGS2, NPY1R*	24	2.2E-04
GO:0035249	synaptic transmission, glutamatergic	3	2.0	*PLAT, GRIN2D, GRID2*	23	2.8E-04
GO:0044057	regulation of system process	9	5.9	*PLAT, CAV1, ACHE, S100P, PTGS2, GRIN2D, OXTR, NPY1R, CALB1*	3	2.7E-03
GO:0048878	chemical homeostasis	12	7.8	*RBP4, CAV1, LCK, GRID2, PCSK9, OXTR, PLLP, NPY1R, CD24, ABCG1, TMPRSS3, CKB*	2	3.4E-03
GO:0050804	regulation of synaptic transmission	6	3.9	*PLAT, ACHE, S100P, PTGS2, OXTR, CALB1*	5	1.8E-03
GO:0042592	homeostatic process	15	9.8	*RBP4, MUC2, CAV1, OXTR, NPY1R, TMPRSS3, ABCG1, CKB, MUC3A, LCK, GRID2, PCSK9, PLLP, CD24, TFF1*	2	4.9E-03

GO:0055088	lipid homeostasis	4	2.6	*CAV1, PCSK9, CD24, ABCG1*	8	1.4E-03
GO:0051969	regulation of transmission of nerve impulse	6	3.9	*PLAT, ACHE, S100P, PTGS2, OXTR, CALB1*	4	2.7E-03
GO:0007610	behavior	11	7.2	*S100P, PTGS2, CXCL5, PDGFA, PPP1R1B, GRIN2D, OXTR, ITGB2, NPY1R, NTSR1, CALB1*	2	4.9E-03
GO:0032570	response to progesterone stimulus	3	2.0	*CAV1, OXTR, THBS1*	16	8.4E-04
GO:0016337	cell-cell adhesion	8	5.2	*JUP, CLDN7, CDH1, ITGB2, CD24, ADAM8, CDH3, PCDHGB2*	3	4.7E-03
GO:0032101	regulation of response to external stimulus	6	3.9	*CAV1, PTGS2, PDGFA, GRID2, CD24, THBS1*	4	4.0E-03
GO:0051050	positive regulation of transport	7	4.6	*RBP4, CAV1, ACHE, SCIN, PCSK9, OXTR, CDH1*	3	5.3E-03
GO:0001894	tissue homeostasis	4	2.6	*MUC2, RBP4, MUC3A, TFF1*	7	3.0E-03
GO:0048167	regulation of synaptic plasticity	4	2.6	*PLAT, S100P, PTGS2, CALB1*	7	3.1E-03
GO:0033273	response to vitamin	4	2.6	*RBP4, PTGS2, PDGFA, MEST*	6	3.5E-03
GO:0050865	regulation of cell activation	6	3.9	*STAT6, PDGFA, LCK, TNFRSF14, CD24, THBS1*	4	6.4E-03
GO:0006873	cellular ion homeostasis	9	5.9	*CAV1, LCK, GRID2, OXTR, PLLP, NPY1R, CD24, TMPRSS3, CKB*	3	9.1E-03
GO:0051480	cytosolic calcium ion homeostasis	5	3.3	*CAV1, LCK, OXTR, NPY1R, CD24*	4	5.2E-03
GO:0007618	mating	3	2.0	*PPP1R1B, PI3, OXTR*	12	2.0E-03
GO:0055082	cellular chemical homeostasis	9	5.9	*CAV1, LCK, GRID2, OXTR, PLLP, NPY1R, CD24, TMPRSS3, CKB*	3	1.0E-02
GO:0001568	blood vessel development	7	4.6	*PLAT, CAV1, PDGFA, CCBE1, THBS1, COL5A1, ANGPTL4*	3	8.7E-03
GO:0010648	negative regulation of cell communication	7	4.6	*CBLC, CAV1, ACHE, PTGS2, STMN3, THBS1, IGFBP5*	3	9.3E-03
GO:0008544	epidermis development	6	3.9	*PTGS2, PDGFA, PPL, CRABP2, GJB3, COL5A1*	3	8.1E-03
GO:0043588	skin development	3	2.0	*PDGFA, GJB3, COL5A1*	11	2.5E-03
GO:0001944	vasculature development	7	4.6	*PLAT, CAV1, PDGFA, CCBE1, THBS1, COL5A1, ANGPTL4*	3	9.9E-03
GO:0010038	response to metal ion	5	3.3	*CAV1, PTGS2, TFF1, THBS1, SOD3*	4	7.6E-03
GO:0007270	nerve-nerve synaptic transmission	3	2.0	*PLAT, GRIN2D, GRID2*	10	3.1E-03

GO:0006875	cellular metal ion homeostasis	6	3.9	*CAV1, LCK, OXTR, NPY1R, CD24, TMPRSS3*	3	1.1E-02
GO:0044092	negative regulation of molecular function	8	5.2	*CBLC, CAV1, GNAI1, HR, PCSK9, NPY1R, NPR3, ANGPTL4*	3	1.4E-02
GO:0031667	response to nutrient levels	6	3.9	*RBP4, CAV1, PTGS2, PDGFA, PCSK9, MEST*	3	1.1E-02
GO:0032526	response to retinoic acid	3	2.0	*RBP4, PDGFA, MEST*	10	3.7E-03

Supplementary Table S5. Enrichment for GO terms in the category 'biological process' for genes downregulated in PC3-GHSROS cells (compared to empty-vector control). $P \leq 0.01$, Fisher's exact test.

GO term	Description	Count	%	Genes	Fold Enrichment	Fisher Exact *P*-value
GO:0007155	cell adhesion	22	1.1	*MTSS1, COL21A1, ADAM23, NRXN3, LRRN2, NELL2, NLGN1, NFASC, LEF1, NEO1, MMRN1, CXADR, PCDH19, CDH12, LAMA1, SRPX, LAMC3, CD33, TGFBI, CNTN1, FCGBP, CXADRP2, EDA*	3	5.8E-06
GO:0022610	biological adhesion	22	1.1	*MTSS1, COL21A1, ADAM23, NRXN3, LRRN2, NELL2, NLGN1, NFASC, LEF1, NEO1, MMRN1, CXADR, PCDH19, CDH12, LAMA1, SRPX, LAMC3, CD33, TGFBI, CNTN1, FCGBP, CXADRP2, EDA*	3	6.0E-06
GO:0007267	cell-cell signaling	16	0.8	*AR, NRXN3, S100A9, NLGN1, CD70, FGF13, GAL, TNFSF9, SLC1A3, KCNN3, HTR7, CD33, DMD, TMOD2, STC1, PCSK5*	2	7.6E-04
GO:0000904	cell morphogenesis involved in differentiation	9	0.4	*NOG, SLITRK4, SLC1A3, NRXN3, KIF5C, NFASC, EOMES, LEF1, EPHB2*	3	1.3E-03
GO:0000902	cell morphogenesis	11	0.5	*LAMA1, NOG, SLITRK4, SLC1A3, NRXN3, DMD, KIF5C, NFASC, EOMES, LEF1, EPHB2*	3	1.7E-03
GO:0001655	urogenital system development	6	0.3	*AGTR1, EYA1, AR, NOG, LEF1, PCSK5*	5	1.2E-03
GO:0043009	chordate embryonic development	10	0.5	*EYA1, AR, NOG, CHD7, ARNT2,*	3	3.2E-03

				EOMES, LEF1, AMOT, ZFPM2, PCSK5		
GO:0009792	embryonic development ending in birth or egg hatching	10	0.5	*EYA1, AR, NOG, CHD7, ARNT2, EOMES, LEF1, AMOT, ZFPM2, PCSK5*	3	3.4E-03
GO:0032989	cellular component morphogenesis	11	0.5	*LAMA1, NOG, SLITRK4, SLC1A3, NRXN3, DMD, KIF5C, NFASC, EOMES, LEF1, EPHB2*	3	3.8E-03
GO:0030509	BMP signaling pathway	4	0.2	*MSX2, NOG, CHRDL1, BMP6*	8	1.3E-03
GO:0006928	cell motion	12	0.6	*LAMA1, MTSS1, VAV3, NRXN3, KIF5C, S100A9, NFASC, AMOT, POU3F2, DNAH5, EPHB2, ARHGDIB*	2	5.4E-03
GO:0003013	circulatory system process	7	0.3	*AGTR1, CHD7, HTR7, RYR2, AMOT, KCNJ12, PCSK5*	3	4.0E-03
GO:0008015	blood circulation	7	0.3	*AGTR1, CHD7, HTR7, RYR2, AMOT, KCNJ12, PCSK5*	3	4.0E-03
GO:0048732	gland development	6	0.3	*EYA1, AR, NOG, LEF1, POU3F2, EDA*	4	3.4E-03
GO:0001837	epithelial to mesenchymal transition	3	0.1	*NOG, EOMES, LEF1*	15	8.9E-04
GO:0021545	cranial nerve development	3	0.1	*SLC1A3, CHD7, EPHB2*	15	1.1E-03
GO:0030182	neuron differentiation	11	0.5	*SLITRK4, SLC1A3, MCOLN3, NRXN3, DGKG, DMD, KIF5C, MAP2, NFASC, POU3F2, EPHB2*	2	7.9E-03
GO:0001822	kidney development	5	0.2	*AGTR1, EYA1, NOG, LEF1, PCSK5*	5	3.8E-03
GO:0001501	skeletal system development	9	0.4	*MSX2, EYA1, TNFRSF11B, NOG, CHD7, CHRDL1, STC1, PCSK5, BMP6*	3	7.8E-03
GO:0035108	limb morphogenesis	5	0.2	*MSX2, NOG, CHD7, LEF1, PCSK5*	5	4.3E-03
GO:0035107	appendage morphogenesis	5	0.2	*MSX2, NOG, CHD7, LEF1, PCSK5*	5	4.3E-03

GO:0048736	appendage development	5	0.2	*MSX2, NOG, CHD7, LEF1, PCSK5*	4	5.1E-03
GO:0060173	limb development	5	0.2	*MSX2, NOG, CHD7, LEF1, PCSK5*	4	5.1E-03
GO:0043627	response to estrogen stimulus	5	0.2	*TNFRSF11B, ARNT2, ANGPT1, SERPINA1, GAL*	4	5.6E-03
GO:0021675	nerve development	3	0.1	*SLC1A3, CHD7, EPHB2*	11	2.4E-03

Supplementary Table S6. Oncomine concepts analysis of positively and negatively correlated PC3-*GHSROS* gene signature. Red: positively correlated gene signature; Black: negatively correlated gene signature. $P \leq 0.01$, Fisher's exact test.

Concept 1 ID	Concept 1 Name	Concept 2 ID	Concept 2 Name	P-value	Odds Ratio	Overlap Size
C41610	PC3 GHSROS downregulated gene list	17697	Cancer Type: Prostate Cancer - Top 10% Over-expressed (Bittner Multi-cancer)	2.17E-08	3.7	32
C41610	PC3 GHSROS downregulated gene list	122189617	Prostate Cancer - Metastasis - Top 10% Under-expressed (Taylor Prostate 3)	3.14E-06	3.1	28
C41610	PC3 GHSROS downregulated gene list	122210891	Cancer Type: Prostate Cancer - Top 5% Under-expressed (Garnett CellLine)	3.94E-06	4.5	16
C41610	PC3 GHSROS downregulated gene list	122208916	Cancer Type: Prostate Cancer - Top 5% Under-expressed (Barretina CellLine)	3.55E-05	3.5	17
C41610	PC3 GHSROS downregulated gene list	122213069	Prostate Cancer - Metastasis - Top 5% Under-expressed (Grasso Prostate)	9.84E-05	3.3	16
C41610	PC3 GHSROS downregulated gene list	28483	Prostate Cancer - Metastasis - Top 5% Under-expressed (Varambally Prostate)	4.13E-04	3	15
C41610	PC3 GHSROS downregulated gene list	23100	Prostate Cancer - Metastasis - Top 10% Under-expressed (LaTulippe Prostate)	4.20E-04	3	16
C41610	PC3 GHSROS downregulated gene list	28344	Prostate Cancer - Metastasis - Top 10% Under-expressed (Vanaja Prostate)	0.001	2.3	21
17697	Cancer Type: Prostate Cancer - Top 10% Over-expressed (Bittner Multi-cancer)	122189617	Prostate Cancer - Metastasis - Top 10% Under-expressed (Taylor Prostate 3)	1.55E-120	4.2	559
17697	Cancer Type: Prostate Cancer - Top 10% Over-expressed (Bittner Multi-cancer)	122213069	Prostate Cancer - Metastasis - Top 5% Under-expressed (Grasso Prostate)	1.33E-120	6.4	356
17697	Cancer Type: Prostate Cancer - Top 10% Over-expressed (Bittner Multi-cancer)	28483	Prostate Cancer - Metastasis - Top 5% Under-expressed (Varambally Prostate)	3.67E-134	6.8	377
17697	Cancer Type: Prostate Cancer - Top 10% Over-expressed (Bittner Multi-cancer)	23100	Prostate Cancer - Metastasis - Top 10% Under-expressed (LaTulippe Prostate)	1.22E-54	4.1	250
17697	Cancer Type: Prostate Cancer - Top 10% Over-expressed (Bittner Multi-cancer)	28344	Prostate Cancer - Metastasis - Top 10% Under-expressed (Vanaja Prostate)	3.10E-189	6.1	619
122189617	Prostate Cancer - Metastasis - Top 10% Under-expressed (Taylor Prostate 3)	122210891	Cancer Type: Prostate Cancer - Top 5% Under-expressed (Garnett CellLine)	1.63E-12	2.1	143

122189617	Prostate Cancer - Metastasis - Top 10% Under-expressed (Taylor Prostate 3)	122208916	Cancer Type: Prostate Cancer - Top 5% Under-expressed (Barretina CellLine)	1.94E-26	2.5	218
122189617	Prostate Cancer - Metastasis - Top 10% Under-expressed (Taylor Prostate 3)	122213069	Prostate Cancer - Metastasis - Top 5% Under-expressed (Grasso Prostate)	6.85E-300	15.4	554
122189617	Prostate Cancer - Metastasis - Top 10% Under-expressed (Taylor Prostate 3)	28483	Prostate Cancer - Metastasis - Top 5% Under-expressed (Varambally Prostate)	1.26E-181	8.7	446
122189617	Prostate Cancer - Metastasis - Top 10% Under-expressed (Taylor Prostate 3)	23100	Prostate Cancer - Metastasis - Top 10% Under-expressed (LaTulippe Prostate)	7.69E-149	8.1	422
122189617	Prostate Cancer - Metastasis - Top 10% Under-expressed (Taylor Prostate 3)	28344	Prostate Cancer - Metastasis - Top 10% Under-expressed (Vanaja Prostate)	1.66E-141	4.8	574
122210891	Cancer Type: Prostate Cancer - Top 5% Under-expressed (Garnett CellLine)	122208916	Cancer Type: Prostate Cancer - Top 5% Under-expressed (Barretina CellLine)	6.57E-135	13.7	232
122210891	Cancer Type: Prostate Cancer - Top 5% Under-expressed (Garnett CellLine)	122213069	Prostate Cancer - Metastasis - Top 5% Under-expressed (Grasso Prostate)	4.37E-06	1.9	65
122210891	Cancer Type: Prostate Cancer - Top 5% Under-expressed (Garnett CellLine)	23100	Prostate Cancer - Metastasis - Top 10% Under-expressed (LaTulippe Prostate)	3.26E-04	1.6	73
122208916	Cancer Type: Prostate Cancer - Top 5% Under-expressed (Barretina CellLine)	122213069	Prostate Cancer - Metastasis - Top 5% Under-expressed (Grasso Prostate)	9.74E-20	2.9	118
122208916	Cancer Type: Prostate Cancer - Top 5% Under-expressed (Barretina CellLine)	28483	Prostate Cancer - Metastasis - Top 5% Under-expressed (Varambally Prostate)	1.44E-09	2.1	93
122208916	Cancer Type: Prostate Cancer - Top 5% Under-expressed (Barretina CellLine)	23100	Prostate Cancer - Metastasis - Top 10% Under-expressed (LaTulippe Prostate)	1.04E-05	1.7	87
122213069	Prostate Cancer - Metastasis - Top 5% Under-expressed (Grasso Prostate)	28483	Prostate Cancer - Metastasis - Top 5% Under-expressed (Varambally Prostate)	1.06E-199	14.8	332
122213069	Prostate Cancer - Metastasis - Top 5% Under-expressed (Grasso Prostate)	23100	Prostate Cancer - Metastasis - Top 10% Under-expressed (LaTulippe Prostate)	5.82E-85	7.7	221
122213069	Prostate Cancer - Metastasis - Top 5% Under-expressed (Grasso Prostate)	28344	Prostate Cancer - Metastasis - Top 10% Under-expressed (Vanaja Prostate)	1.21E-75	4.7	288
28483	Prostate Cancer - Metastasis - Top 5%	23100	Prostate Cancer - Metastasis - Top 10%	1.59E-59	5.7	192

	Under-expressed (Varambally Prostate)		Under-expressed (LaTulippe Prostate)			
28483	Prostate Cancer - Metastasis - Top 5% Under-expressed (Varambally Prostate)	28344	Prostate Cancer - Metastasis - Top 10% Under-expressed (Vanaja Prostate)	3.84E-80	4.7	302
23100	Prostate Cancer - Metastasis - Top 10% Under-expressed (LaTulippe Prostate)	28344	Prostate Cancer - Metastasis - Top 10% Under-expressed (Vanaja Prostate)	2.79E-54	4	258
C41610	PC3 GHSROS downregulated gene list	122199554	Prostate Carcinoma - Dead at 3 Years - Top 10% Under-expressed (Setlur Prostate)	0.003	2.9	12
C41610	PC3 GHSROS downregulated gene list	122189630	Prostate Carcinoma - Advanced Gleason Score - Top 5% Under-expressed (Taylor Prostate 3)	0.004	2.5	13
C41610	PC3 GHSROS downregulated gene list	122189606	Prostate Carcinoma - Recurrence at 5 Years - Top 5% Under-expressed (Taylor Prostate 3)	0.004	2.5	13
122199554	Prostate Carcinoma - Dead at 3 Years - Top 10% Under-expressed (Setlur Prostate)	122189630	Prostate Carcinoma - Advanced Gleason Score - Top 5% Under-expressed (Taylor Prostate 3)	3.07E-37	4.7	141
122199554	Prostate Carcinoma - Dead at 3 Years - Top 10% Under-expressed (Setlur Prostate)	122189606	Prostate Carcinoma - Recurrence at 5 Years - Top 5% Under-expressed (Taylor Prostate 3)	3.29E-24	3.4	130
122189630	Prostate Carcinoma - Advanced Gleason Score - Top 5% Under-expressed (Taylor Prostate 3)	122189606	Prostate Carcinoma - Recurrence at 5 Years - Top 5% Under-expressed (Taylor Prostate 3)	0.00E+00	26.5	493
C41601	PC3 GHSROS upregulated gene list_RNAseq	29459	Prostate Carcinoma vs. Normal - Top 1% Over-expressed (Yu Prostate)	4.78E-04	12.5	4
C41602	PC3 GHSROS upregulated gene list_RNAseq	122189633	Prostate Carcinoma - Advanced N Stage - Top 5% Over-expressed (Taylor Prostate 3)	0.002	3.5	9
C41603	PC3 GHSROS upregulated gene list_RNAseq	17807	Prostate Adenocarcinoma - Advanced Stage - Top 1% Over-expressed (Bittner Prostate)	0.003	7.5	4
C41604	PC3 GHSROS upregulated gene list_RNAseq	23091	Prostate Carcinoma vs. Normal - Top 10% Over-expressed (LaTulippe Prostate)	0.003	3.4	10
29459	Prostate Carcinoma vs. Normal - Top 1% Over-expressed (Yu Prostate)	17807	Prostate Adenocarcinoma - Advanced Stage - Top 1% Over-expressed (Bittner Prostate)	3.33E-04	7.2	6
29459	Prostate Carcinoma vs. Normal - Top 1% Over-expressed (Yu Prostate)	23091	Prostate Carcinoma vs. Normal - Top 10% Over-expressed (LaTulippe Prostate)	5.61E-07	3.8	25
122189633	Prostate Carcinoma - Advanced N Stage - Top 5% Over-	23091	Prostate Carcinoma vs. Normal - Top 10% Over-	3.73E-06	2	61

	expressed (Taylor Prostate 3)		expressed (LaTulippe Prostate)			
17807	Prostate Adenocarinoma - Advanced Stage - Top 1% Over-expressed (Bittner Prostate)	23091	Prostate Carcinoma vs. Normal - Top 10% Over-expressed (LaTulippe Prostate)	6.07E-04	2.5	20

Supplementary Table S7. Differentially expressed genes in PC3-GHSROS and LNCaP-GHSROS cells compared to the Grasso Oncomine dataset. The Grasso dataset includes 59 localized and 35 metastatic prostate tumors. Red: higher expression in metastatic tumors; Black: lower expression in metastatic tumors. Fold-changes are log_2 transformed; *Q*-value denotes the false discovery rate (FDR; Benjamini-Hochberg)-adjusted *P*-value.

Gene Symbol	**Gene Name**	**Reporter ID**	**Fold Change**	***P*-value**	***Q*-value**
AASS	aminoadipate-semialdehyde synthase	A_23_P8754	-1.5	6.0E-03	2.5E-02
CHRDL1	chordin-like 1	A_24_P168925	-48.0	3.6E-21	2.9E-18
CNTN1	contactin 1	A_23_P204541	-19.3	1.6E-16	3.4E-14
DIRAS1	DIRAS family, GTP-binding RAS-like 1	A_23_P386942	2.2	3.5E-07	4.4E-06
FBXL16	F-box and leucine-rich repeat protein 16	A_23_P406385	6.0	2.5E-08	4.7E-07
IFI16	interferon, gamma-inducible protein 16	A_23_P160025	-2.2	1.1E-05	8.8E-05
MUM1L1	melanoma associated antigen (mutated) 1-like 1	A_23_P73571	-8.8	7.9E-11	2.5E-09
TFF2	trefoil factor 2	A_23_P57364	1.3	6.4E-04	3.1E-03
TP53I11	tumor protein p53 inducible protein 11	A_23_P150281	1.5	4.8E-05	3.1E-04
ZNF467	zinc finger protein 467	A_23_P59470	3.5	5.4E-07	6.3E-06

Supplementary Table S8. Differentially expressed genes in PC3-GHSROS and LNCaP-GHSROS cells compared to the Taylor Oncomine dataset. The Taylor dataset includes 123 localized and 35 metastatic prostate tumors. Red: higher expression in metastatic tumors; Black: lower expression in metastatic tumors. Fold-changes are log_2 transformed; *Q*-value denotes the false discovery rate (FDR; Benjamini-Hochberg)-adjusted *P*-value.

Gene Symbol	**Gene Name**	**Reported ID**	**Fold Change**	***P*-value**	***Q*-value**
AASS	aminoadipate-semialdehyde synthase	10093	-1.5	3.9E-05	1.7E-03
CHRDL1	chordin-like 1	20828	-5.0	1.6E-18	1.3E-15
CNTN1	contactin 1	6403	-3.5	4.2E-22	6.8E-19
DIRAS1	DIRAS family, GTP-binding RAS-like 1	20799	1.1	1.2E-02	1.4E-01
FBXL16	F-box and leucine-rich repeat protein 16	21824	1.1	8.0E-03	1.2E-01
IFI16	interferon, gamma-inducible protein 16	9878	-1.5	8.4E-05	3.3E-03
MUM1L1	melanoma associated antigen (mutated) 1-like 1	21313	-1.3	1.0E-02	1.3E-01
TFF2	trefoil factor 2	9774	1.1	3.6E-02	2.4E-01
TP53I11	tumor protein p53 inducible protein 11	4038	1.1	2.2E-02	1.9E-01
ZNF467	zinc finger protein 467	25037	1.3	2.8E-04	1.7E-02

Supplementary Table S9. Disease-free survival (DFS) analysis of differentially expressed genes (in PC3-GHSROS cells, LNCaP-GHSROS cells and clinical metastatic tumors) in human datasets. Patients, in the Taylor (*N*=150; *n*=123 localized and *n*=27 metastatic tumors) and TCGA-PRAD (*N*=489; localized tumors) datasets, were stratified into two groups by *k*-means clustering of gene expression (*k*=2). The log-rank test, was used to assign statistical significance, with $P \leq 0.05$ considered significant (shown in bold). The Cox *P*-value and absolute hazard ratio (HR) between *k*-means cluster 1 and 2 for each gene are indicated. Overall median disease-free survival (DFS) in days are indicated for each cluster.

	**Taylor (*N*=150)**					**TCGA-PRAD (*N*=489)**				
**gene**	**log-rank *P***	**Cox *P***	**Absolute HR**	**Overall median DFS cluster 1**	**Overall median DFS cluster 2**	**log-rank *P***	**Cox *P***	**Absolute HR**	**Overall median DFS cluster 1**	**Overall median DFS cluster 2**
ZNF467	**0.0027**	**0.0039**	2.7	174	871	**0.000050**	**0.000026**	2.5	546	685
CHRDL1	**0.0047**	**0.0062**	2.5	840	402	**0.0079**	**0.0071**	1.8	649	640
FBXL16	**0.017**	**0.020**	2.2	300	871	0.089	0.087	1.5	627	663
DIRAS1	0.09	0.099	1.7	709	329	**0.012**	**0.011**	1.7	425	723
TFF2	0.11	0.11	1.7	840	125	0.84	0.089	1.1	648	896
CNTN1	0.13	0.14	1.6	701	457	0.10	0.094	1.4	627	691
IFI16	0.27	0.28	1.5	579	181	0.95	0.95	1.0	671	648
AASS	0.62	0.63	1.2	843	348	0.35	0.35	1.2	552	697
MUM1L1	0.78	0.78	1.1	472	676	0.14	0.14	1.4	765	426
TP53I11	0.98	0.98	1.0	122	843	0.57	0.57	1.1	533	751

Supplementary Table S10. Overview of human Affymetrix exon array datasets interrogated.

resource	unique ID	tissue/cell type	type	N	reference
ArrayExpress	E-MEXP-2644	lung (18 benign and 18 cancer)	tissues	36	[86]
ArrayExpress	E-MEXP-3931	THP1 (acute monocytic leukemia)	cell lines	12	[87]
ArrayExpress	E-MTAB-1273	induced pluripotent stem (iPS) cells derived from glioblastoma-derived neural stem cells	primary cells	16	[88]
ArrayExpress	E-MTAB-2471	large B-cell lymphoma	tissues	16	[89]
Affymetrix web site	goo.gl/rBWrFv	breast, cerebellum, heart, kidney, liver, muscle, pancreas, prostate, spleen, testes, thyroid, mixture	tissues	53	-
Affymetrix web site	goo.gl/Yack5K	colon cancer (10 benign and 10 cancer)	tissues	20	-
GEO	GSE11967	thymus (4 benign and 4 cancer)	tissues	8	[90]
GEO	GSE16732	breast (cancer)	cell lines	41	[91]
GEO	GSE18927	NIH Epigenomics Roadmap Initiative (stem cells and primary *ex vivo* tissues)	tissues and primary cells	99	[92,93,94,94,96,97]
GEO	GSE19090	ENCODE Project Consortium (84 cell lines and primary cells)	cell lines and primary cells	182	[98, 99]
GEO	GSE19891	HeLa (cervical cancer)	cell lines	15	[100]
GEO	GSE20342	MCF7 (breast cancer)	cell lines	32	[101]
GEO	GSE20567	HL60, THP-1, U937 (myeloid leukemia)	cell lines	17	[102]
GEO	GSE21034	prostate (cancer)	tissues	310	[24]
GEO	GSE21163	pancreas (1 benign and 6 cancer)	cell lines	22	[103, 104]
GEO	GSE21337	acute myeloid leukemia	tissues	64	[105]
GEO	GSE21840	MCF7 (breast cancer)	cell lines	6	[106, 107]
GEO	GSE23361	lung (cancer)	tissues	12	[108]
GEO	GSE23514	HeLa S3 (cervical cancer)	cell lines	12	[109]
GEO	GSE23768	breast, lung, ovarian and prostate cancer	tissues	153	[110]
GEO	GSE24778	K562 (chronic myelogenous leukemia)	cell lines	10	[111]
GEO	GSE29682	breast, central nervous system, colon, leukemia, melanoma, lung, ovary, prostate, kidney (cancer)	cell lines	178	[112, 113]
GEO	GSE29778	HEK293 (embryonic kidney)	cell lines	12	[114]
GEO	GSE30472	brain (cancer; glioma)	tissues	55	[115]
GEO	GSE30521	prostate (benign and cancer)	tissues	23	[116]
GEO	GSE30727	stomach (cancer)	tissues	60	[117]
GEO	GSE32875	LNCaP (prostate cancer)	cell lines	8	[118]
GEO	GSE37138	lung (cancer)	tissues	117	[119]
GEO	GSE40871	acute myeloid leukemia	primary cells	67	[120]
GEO	GSE43107	brain (cancer; glioma)	tissues	95	[121, 122]
GEO	GSE43754	bone marrow stem and progenitor cells (chronic myeloid leukemia)	cells	20	[123]
GEO	GSE43830	WI38 (fetal lung fibroblasts)	cell lines	6	[124]
GEO	GSE45379	HeLa (cervical cancer)	cell lines	6	-
GEO	GSE46691	prostate (cancer)	tissues	545	[125, 126]

GEO	GSE47032	kidney (cancer)	tissues	40	[127]
GEO	GSE53405	MCF10A (benign)	cell lines	26	-
GEO	GSE57076	THP1 (acute monocytic leukemia)	cell lines	7	[128]
GEO	GSE57933	bladder (cancer)	tissues	199	[129]
GEO	GSE58598	breast (cancer)	tissues	10	-
GEO	GSE62116	prostate (cancer)	tissues	235	[126, 130, 131]
GEO	GSE62667	prostate (cancer)	tissues	182	[132]
GEO	GSE67312	bladder (cancer)	primary xenografts	10	[133]
GEO	GSE68591	sarcoma (84 cancer) and 5 benign	cell lines	75	[134]
GEO	GSE71010	neutrophils (cystic fibrosis and healthy controls)	cells	93	[135]
GEO	GSE72291	prostate (cancer)	tissues	139	[136]
GEO	GSE78246	brain (schizophrenia, bipolar disorder, major depressive disorder, and controls)	tissues	20	[137]
GEO	GSE79956	prostate (cancer)	tissues	211	-
GEO	GSE79957	prostate (cancer)	tissues	260	-
GEO	GSE80683	prostate (cancer)	tissues	17	-
GEO	GSE9342	T-cell acute lymphoblastic leukaemia	cell lines	17	-
GEO	GSE9385	brain (26 glioblastomas, 22 oligodendrogliomas and 6 control brain samples)	tissues	55	[138]

Supplementary Table S11. Primers used in this study.

Primer	Gene name	Primer sequence (5'-3')
GHSROS	growth hormone secretagogue receptor opposite strand	ACATTCAGCAAATCCAGTTAATGACA
		CGACTGGAGCACGAGGACACTTGA
GHSROS-RT linker		CGACTGGAGCACGAGGACACTGACAACAGAATTCACTACTTCCCCAAA
AR	androgen receptor	CTGGACACGACAACAACCAG
		CAGATCAGGGGCGAAGTAGA
NTSR1	neurotensin receptor 1 (high affinity)	Proprietary – QIAGEN QuantiTect Primer Assay QT00018494
TFF1	trefoil factor 1	Proprietary - QIAGEN QuantiTect Primer Assay QT00209608
TFF2	trefoil factor 2	Proprietary - QIAGEN QuantiTect Primer Assay QT00001785
MUC5B	mucin 5B, oligomeric mucus/ gel-forming	Proprietary - QIAGEN QuantiTect Primer Assay QT01322818
PPP2R2C	protein phosphatase 2, regulatory subunit B, gamma	Proprietary - QIAGEN QuantiTect Primer Assay QT01006383
RPL32	ribosomal protein L32 (housekeeping gene)	CCCCTTGTGAAGCCCAAGA
		GACTGGTGCCGGATGAACTT
ACTB	actin beta (housekeeping gene)	ACTCTTCCAGCCTTCCTTCCT
		CAGTGATCTCCTTCTGCATCCT
GAPDH	glyceraldehyde-3-phosphate dehydrogenase (housekeeping gene)	AATCCCATCACCATCTTCCA
		AAATGAGCCCCAGCCTTC
HPRT	hypoxanthine phosphoribosyltransferase 1 (housekeeping gene)	CAGTCAACGGGGGACATAAA
		AGAGGTCCTTTTCACCAGCAA

Supplementary Dataset 1. Differentially expressed genes in LNCaP-GHSROS cells. Compared to empty vector control. Red: higher expression in LNCaP-GHSROS cells; Black: lower expression in LNCaP-GHSROS cells. Fold-changes are $\log_2$ transformed; *Q*-value denotes the false discovery rate (FDR; Benjamini-Hochberg)-adjusted *P*-value (cutoff ≤ 0.05).

(provided in a separate file).

Supplementary Dataset 2. Enrichment for GO terms in the category ‘biological process’ for genes upregulated in LNCaP-GHSROS cells (compared to empty-vector control). $P \leq 0.01$, Fisher's exact test.

(provided in a separate file).

Supplementary Dataset 3. Enrichment for GO terms in the category 'biological process' for genes downregulated in LNCaP-GHSROS cells (compared to empty-vector control). $P \leq 0.01$, Fisher's exact test.

(provided in a separate file).

SUPPLEMENTARY METHODS

Identification of *GHSROS* transcription in exon array datasets

To assess *GHSROS* expression, we interrogated Affymetrix GeneChip Exon 1.0 ST arrays, strand-specific oligonucleotide microarrays with probes for known and predicted exons (hereafter termed exon arrays). Exon arrays are comparable to RNA-seq in experiments aimed at assessing exon expression (*i.e.* gene isoforms) and suitable for experiments where the exon of interest is known[139, 140]. In the Exon 1.0 ST array, known (genes and ESTs) and putative exons are combined to form 'transcript clusters', with each exon defined as a probe set (typically, a set of 2-4 probes). By combining all probe sets, the expression of a transcript cluster (known or putative gene) can be measured (see https://goo.gl/4RSTG3). To identify probe set(s) corresponding to *GHSROS*, we downloaded the Exon 1.0 ST probe annotation file from NCBI (NCBI Gene Expression Omnibus (GEO) accession no. GPL5188). Full-length *GHSROS* (1.1 kb) was aligned to the human genome (NCBI36/hg18; March 2006 assembly) to generate genomic coordinates compatible with the probe file (chr3:173,646,439-173,647,538). Next, the probe annotation file (GPL5188) was interrogated to reveal probe sets spanning *GHSROS* by entering the following command in a UNIX terminal window:

```
sed 's/#.*//' GPL5188.txt | awk -F " " '{print $2}' $1 | grep $(echo
"chr3:173646846-173647446" | perl -ne 'print if /\bchr3\:173646[0-
9][0-9][0-9]-173647[0-9][0-9][0-9]/' $1) GPL5188.txt
```

This revealed a probe set, 2652604, consisting of 4 probes complementary to *GHSROS*.

Cell and tissue exon array data were downloaded from NCBI GEO[141], EBI ArrayExpress[142] and the Affymetrix web site (see Supplementary Table S10). GEO datasets were bulk-downloaded using v3.6.2.117442 of the Aspera Connect Linux software (Aspera, Emeryville, CA, USA). In total, 3,924 samples were downloaded, corresponding to ~46% of all exon array data deposited in the NCBI GEO database. Arrays (individual CEL files) were normalized (output on a $\log_2$ scale, centered at 0) using the *SCAN* function in the R package 'SCAN.UPC'[143, 144]. SCAN normalizes each array (sample) individually by removing background noise (probe- and array-specific) data from within the array. Next, arrays were interrogated using the *UPC* function in 'SCAN.UPC'. UPC outputs standardized expression values (UPC value), ranging from 0 to 1, which indicate whether a gene is actively transcribed in a sample of interest: higher values indicate that a gene is 'active'[143]. UPC scores are platform-independent and allow cross-experimental and cross-platform integration.

Evaluation of *GHSR/GHSROS* transcription in deep RNA-seq dataset

It has been estimated that reliable detection of low abundance transcripts in humans warrants very deep sequencing (> 200 million reads per sample[145]) – far beyond most current datasets. To illustrate, we considered the expression of *GHSR*/*GHSROS* in a comparable clinical dataset. Publicly available RNA-seq data (NCBI GEO accession no. GSE31528) from eight subjects with metastatic castration-resistant prostate cancer (bone marrow metastases)[146] were interrogated. Briefly, total RNA-seq was performed on random-primed paired end read libraries, to ensure consistent transcript coverage[147, 148,149], generating an average of 160M reads per sample. Paired-end FASTQ files were aligned to the human genome (UCSC build hg19) using the spliced-read mapper TopHat

(v2.0.9)[148] and reference gene annotations to guide the alignment. BigWig sequencing tracks for the UCSC genome browser[150] were obtained from TopHat-generated BAM files (indexed by samtools v1.2[151]) using a local instance of the *bamCoverage* command in deepTools v2.5.4[152]. BigWig files were visualized in the UCSC genome browser (hg19). A region with less than ~10 supporting reads can be considered to have low coverage, rendering active transcription difficult to interpret[152, 153].

RNA secondary structure prediction
The ViennaRNA web server was employed[154] to predict the secondary structure of *GHSROS* and its minimum free energy[155].

RNA sequencing of PC3-GHSROS cells
RNA was extracted from *in vitro* cultured PC3-GHSROS cells and controls, as outlined in the manuscript body. RNA purity was analysed using an Agilent 2100 Bioanalyzer, and RNA with an RNA Integrity Number (RIN) above 7 used for RNA-seq. Strand-specific RNA-sequencing (RNA-seq) was performed by Macrogen, South Korea. A TruSeq stranded mRNA library (Illumina) was constructed and RNA sequencing performed (50 million reads) on a HiSeq 2000 instrument (Illumina) with 100bp paired end reads. Pre-processing of raw FASTQ reads, including elimination of contamination adapters, was performed with scythe v0.994 (https://github.com/vsbuffalo/scythe). Paired-end human FASTQ files were aligned to the human genome, UCSC build hg19 using the spliced-read mapper TopHat (v2.0.9)[148] and reference gene annotations to guide the alignment.

Raw gene counts were computed from TopHat-generated BAM files using featureCounts v1.4.5-p1[156], counting coding sequence (CDS) features of the UCSC hg19 gene annotation file (gtf). FeatureCounts output files were analysed using the R programming language (v.3.2.2). Briefly, raw counts were normalized by Trimmed Mean of M-values (TMM) correction[157, 158]. Library size-normalized read counts (per million; CPM) were subjected to the voom function (variance modelling at the observation-level) in limma v3.22.1 (Linear Models for Microarray Data)[159, 160], with trend=TRUE for the eBayes function and correction for multiple testing (Benjamini-Hochberg false discovery rate of cut-off, *Q*-value, set at 0.05). Genes with at least a 1.5 $\log_2$ fold-change difference in expression between PC3-GHSROS and PC3-vector (empty vector) cells were defined as differentially expressed. Although validation is not required, as RNA-seq gives very accurate measurements of relative expression across a broad dynamic range[160], selected differentially regulated genes were validated using quantitative reverse-transcription PCR (qRT-PCR) (see manuscript body and table S11). Detailed gene annotations were obtained by querying Ensembl with the R/Bioconductor package 'biomaRt'[161].

RNA sequencing of LNCaP-GHSROS cells

RNA was extracted from LNCaP-GHSROS xenograft tumors and controls (empty vector control lentiviral constructs), as outlined in the manuscript body. RNA purity was analysed using an Agilent 2100 Bioanalyzer, and RNA with an RNA Integrity Number (RIN) above 7 used for RNA-seq. Strand-specific RNA-seq was performed by the South Australian Health and Medical Research Institute (SAHMRI, Adelaide, SA, Australia). A TruSeq stranded mRNA library (Illumina) was constructed and RNA sequencing performed (35 million reads) on a Nextseq 500 instrument (Illumina) with 75bp single end reads. Pre-processing of raw FASTQ reads, including elimination of contamination adapters, was performed with scythe v0.994 (https://github.com/vsbuffalo/scythe). Human (xenograft tumor; the graft) and mouse (the host) RNA-seq reads were separated using Xenome[162] on the trimmed FASTQ files, leaving ~20M human reads. Reads were aligned to the human genome and processed as described for PC3-GHSROS cells above. Genes differentially expressed in LNCaP-GHSROS cells (cutoff set at $\log_2$ 1.5-fold-change and $Q \leq 0.05$) were imported into the GSEA (Gene Set Enrichment Analysis) program[163].

Survival analysis

Two datasets were interrogated: Taylor[23](123 localized and 27 metastatic prostate tumors) and TCGA-PRAD from The Cancer Genomics Atlas (TCGA) consortium, which contains tumors from patients with moderate- (~39% Gleason 6 and 3 + 4) and high- (~61% Gleason 4+3 and Gleason 8-10) risk localized prostate carcinoma[24]. Briefly, in the case of TCGA-PRAD, the UCSC Xena Browser[32] was used to obtain normalized gene expression values, represented as $\log_2$(normalized counts+1), from the 'TCGA TARGET GTeX' dataset consisting of ~12,000 tissue samples from 31 cancers[161]. To obtain up-to-date overall survival (OS) and disease-free survival (DFS) information, we manually queried cBioPortal for Cancer Genomics[34, 35] (last accessed 05.08.16).

We performed non-hierarchical *k*-means clustering[164, 165] to partition patients into groups with similar gene expression patterns[166]. The following 10 genes obtained by Oncomine meta-analysis (see above) were assessed: *AASS*, *CHRDL1*, *CNTN1*, *DIRAS1*, *FBXL16*, *IFI16*, *MUM1L1*, *TP53I11*, *TFF2*, and *ZNF467.* Clustering was performed using the *kmeans* function in the R package 'stats' with two clusters/groups (*k=2*) and the best cluster pair after 500 runs (*nstart=500*) was retained[167]. Kaplan-Meier survival analysis[168] was performed with the R package 'survival'[169], fitting survival curves (*survfit*) and computing log-rank *P*-values using the *survdiff* function, with *rho=0* (equivalent to the method employed by UCSC Xena; see https://goo.gl/4knf62). Survival curves were plotted when survival was significantly different between two groups (log-rank $P \leq 0.05$). We used the *coxph* function in the R package 'survival' to test the prognostic significance of genes (that is: we implemented the Cox proportional hazard model to analyze the association of gene expression with patient survival)[170], with $P \leq 0.05$ (Wald test) considered significant. Because there is a single categorical covariate (*k*-means cluster; group), the *P*-values from the log-rank and the Cox regression tests are comparable. We considered groups (clusters) that had fewer than 10 samples with a recorded event unreliable.

A scaled heat map (unsupervised hierarchical clustering by Euclidean distance) was generated in R using heatmap.3 (available at https://goo.gl/Yd9aTY) and a custom R script.

.

SUPPLEMENTARY REFERENCES

86 Langer W. *et al*. Exon array analysis using re-defined probe sets results in reliable identification of alternatively spliced genes in non-small cell lung cancer. *BMC Genomics* 2010; **11**: 676.

87 Folkard D.L. *et al*. Suppression of LPS-induced transcription and cytokine secretion by the dietary isothiocyanate sulforaphane. *Mol Nutr Food Res* 2014; **58**: 2286-2296.

88 Stricker S.H. *et al*. Widespread resetting of DNA methylation in glioblastoma-initiating cells suppresses malignant cellular behavior in a lineage-dependent manner. *Genes Dev* 2013; **27**: 654-669.

89 Riihijarvi S. *et al*. Prognostic influence of macrophages in patients with diffuse large B-cell lymphoma: a correlative study from a Nordic phase II trial. *Haematologica* 2015; **100**: 238-245.

90 Soreq L. *et al*. Identifying alternative hyper-splicing signatures in MG-thymoma by exon arrays. *PLoS One* 2008; **3**: e2392.

91 Riaz M. *et al*. Low-risk susceptibility alleles in 40 human breast cancer cell lines. *BMC Cancer* 2009; **9**: 236.

92 Bernstein B.E. *et al*. The NIH Roadmap Epigenomics Mapping Consortium. *Nat Biotechnol* 2010; **28**: 1045-1048.

93 Maurano M.T. *et al*. Systematic localization of common disease-associated variation in regulatory DNA. *Science* 2012; **337**: 1190-1195.

94 Neph S. *et al*. An expansive human regulatory lexicon encoded in transcription factor footprints. *Nature* 2012; **489**: 83-90.

95 Polak P. *et al*. Cell-of-origin chromatin organization shapes the mutational landscape of cancer. *Nature* 2015; **518**: 360-364.

96 Polak P. *et al*. Reduced local mutation density in regulatory DNA of cancer genomes is linked to DNA repair. *Nat Biotechnol* 2014; **32**: 71-75.

97 Schultz M.D. *et al*. Human body epigenome maps reveal noncanonical DNA methylation variation. *Nature* 2015; **523**: 212-216.

98 Hansen R.S. *et al*. Sequencing newly replicated DNA reveals widespread plasticity in human replication timing. *Proc Natl Acad Sci U S A* 2010; **107**: 139-144.

99 Thurman R.E. *et al*. The accessible chromatin landscape of the human genome. *Nature* 2012; **489**: 75-82.

100 Younis I. *et al*. Rapid-response splicing reporter screens identify differential regulators of constitutive and alternative splicing. *Mol Cell Biol* 2010; **30**: 1718-1728.

101 Dutertre M. *et al*. Estrogen regulation and physiopathologic significance of alternative promoters in breast cancer. *Cancer Res* 2010; **70**: 3760-3770.

102 Kitamura H. *et al*. Ubiquitin-specific protease 2-69 in macrophages potentially modulates metainflammation. *FASEB J* 2013; **27**: 4940-4953.

103 Omura N. *et al*. Cyclooxygenase-deficient pancreatic cancer cells use exogenous sources of prostaglandins. *Mol Cancer Res* 2010; **8**: 821-832.

104 Vincent A. *et al*. Genome-wide analysis of promoter methylation associated with gene expression profile in pancreatic adenocarcinoma. *Clin Cancer Res* 2011; **17**: 4341-4354.

105 Risueno A. *et al*. A robust estimation of exon expression to identify alternative spliced genes applied to human tissues and cancer samples. *BMC Genomics* 2014; **15**: 879.

106 Dutertre M. *et al*. A recently evolved class of alternative 3'-terminal exons involved in cell cycle regulation by topoisomerase inhibitors. *Nat Commun* 2014; **5**: 3395.

107 Dutertre M. *et al*. Cotranscriptional exon skipping in the genotoxic stress response. *Nat Struct Mol Biol* 2010; **17**: 1358-1366.

108 Calverley D.C. *et al*. Significant downregulation of platelet gene expression in metastatic lung cancer. *Clin Transl Sci* 2010; **3**: 227-232.

109 Llorian M. *et al*. Position-dependent alternative splicing activity revealed by global profiling of alternative splicing events regulated by PTB. *Nat Struct Mol Biol* 2010; **17**: 1114-1123.

110 Kan Z. *et al*. Diverse somatic mutation patterns and pathway alterations in human cancers. *Nature* 2010; **466**: 869-873.

111 Pencovich N., Jaschek R., Tanay A., Groner Y. Dynamic combinatorial interactions of RUNX1 and cooperating partners regulates megakaryocytic differentiation in cell line models. *Blood* 2011; **117**: e1-e14.

112 Kohn K.W., Zeeberg B.M., Reinhold W.C., Pommier Y. Gene expression correlations in human cancer cell lines define molecular interaction networks for epithelial phenotype. *PLoS One* 2014; **9**: e99269.

113 Reinhold W.C. *et al*. Exon array analyses across the NCI-60 reveal potential regulation of TOP1 by transcription pausing at guanosine quartets in the first intron. *Cancer Res* 2010; **70**: 2191-2203.

114 Mukherjee N. *et al*. Integrative regulatory mapping indicates that the RNA-binding protein HuR couples pre-mRNA processing and mRNA stability. *Mol Cell* 2011; **43**: 327-339.

115 Gravendeel L.A. *et al*. Gene expression profiles of gliomas in formalin-fixed paraffin-embedded material. *Br J Cancer* 2012; **106**: 538-545.

116 Agell L. *et al*. A 12-gene expression signature is associated with aggressive histological in prostate cancer: SEC14L1 and TCEB1 genes are potential markers of progression. *Am J Pathol* 2012; **181**: 1585-1594.

117 Hong S.H. *et al*. Upregulation of adenylate cyclase 3 (ADCY3) increases the tumorigenic potential of cells by activating the CREB pathway. *Oncotarget* 2013; **4**: 1791-1803.

118 Rajan P. *et al*. Identification of novel androgen-regulated pathways and mRNA isoforms through genome-wide exon-specific profiling of the LNCaP transcriptome. *PLoS One* 2011; **6**: e29088.

119 Baty F. *et al*. EGFR exon-level biomarkers of the response to bevacizumab/erlotinib in non-small cell lung cancer. *PLoS One* 2013; **8**: e72966.

120 Klco J.M. *et al*. Genomic impact of transient low-dose decitabine treatment on primary AML cells. *Blood* 2013; **121**: 1633-1643.

121 Erdem-Eraslan L. *et al*. Intrinsic molecular subtypes of glioma are prognostic and predict benefit from adjuvant procarbazine, lomustine, and vincristine chemotherapy in combination with other prognostic factors in anaplastic oligodendroglial brain tumors: a report from EORTC study 26951. *J Clin Oncol* 2013; **31**: 328-336.

122 Mack S,C. *et al*. Epigenomic alterations define lethal CIMP-positive ependymomas of infancy. *Nature* 2014; **506**: 445-450.

123 Gerber J.M. *et al*. Genome-wide comparison of the transcriptomes of highly enriched normal and chronic myeloid leukemia stem and progenitor cell populations. *Oncotarget* 2013; **4**: 715-728.

124 Tripathi V. *et al*. Long noncoding RNA MALAT1 controls cell cycle progression by regulating the expression of oncogenic transcription factor B-MYB. *PLoS Genet* 2013; **9**: e1003368.

125 Erho N. *et al*. Discovery and validation of a prostate cancer genomic classifier that predicts early metastasis following radical prostatectomy. *PLoS One* 2013; **8**: e66855.

126 Zhao S.G. *et al*. The Landscape of Prognostic Outlier Genes in High-Risk Prostate Cancer. *Clin Cancer Res* 2016; **22**: 1777-1786.

127 Valletti A. *et al*. Genome-wide analysis of differentially expressed genes and splicing isoforms in clear cell renal cell carcinoma. *PLoS One* 2013; **8**: e78452.

128 Silva-Martinez G.A. *et al*. Arachidonic and oleic acid exert distinct effects on the DNA methylome. *Epigenetics* 2016; **11**: 321-334.

129 Mitra A.P. *et al*. Discovery and validation of novel expression signature for postcystectomy recurrence in high-risk bladder cancer. *J Natl Cancer Inst* 2014; **106**.

130 Karnes R.J. *et al*. Validation of a genomic classifier that predicts metastasis following radical prostatectomy in an at risk patient population. *J Urol* 2013; **190**: 2047-2053.

131 Tsai H. *et al*. Cyclin D1 Loss distinguishes prostatic small-cell carcinoma from most prostatic adenocarcinomas. *Clin Cancer Res* 2015; **21**: 5619-5629.

132 Klein E.A. *et al*. A genomic classifier improves prediction of metastatic disease within 5 years after surgery in node-negative high-risk prostate cancer patients managed by radical prostatectomy without adjuvant therapy. *Eur Urol* 2015; **67**: 778-786.

133 Jager W. *et al*. Patient-derived bladder cancer xenografts in the preclinical development of novel targeted therapies. *Oncotarget* 2015; **6**: 21522-21532.

134 Teicher B.A. *et al*. Sarcoma cell line screen of oncology drugs and investigational agents identifies patterns associated with gene and microRNA expression. *Mol Cancer Ther* 2015; **14**: 2452-2462.

136 Hu Z., Jiang K., Frank M.B., Chen Y., Jarvis J.N. Complexity and specificity of the neutrophil transcriptomes in juvenile idiopathic arthritis. *Sci Rep* 2016; **6**: 27453.

137 Morgan L.Z. *et al*. Quantitative trait locus and brain expression of HLA-DPA1 offers evidence of shared immune alterations in psychiatric disorders. *Microarrays (Basel)* 2016; **5**: 6.

138 French P.J. *et al*. Identification of differentially regulated splice variants and novel exons in glial brain tumors using exon expression arrays. *Cancer Res* 2007; **67**: 5635-5642.

139 Dapas M., Kandpal M., Bi Y., Davuluri R.V. Comparative evaluation of isoform-level gene expression estimation algorithms for RNA-seq and exon-array platforms. *Brief Bioinform* 2017; **18**: 260-269.

140 Griffith M. *et al*. Alternative expression analysis by RNA sequencing. *Nat Methods* 2010; **7**: 843-847.

141 Barrett T. *et al*. NCBI GEO: archive for functional genomics datasets--update. *Nucleic Acids Res* 2013; **41**: D991-995.

142 Brazma A. *et al*. ArrayExpress--a public repository for microarray gene expression data at the EBI. *Nucleic Acids Res* 2003; **31**: 68-71.

143 Piccolo S.R. *et al*. A single-sample microarray normalization method to facilitate personalized-medicine workflows. *Genomics* 2012; **100**: 337-344.

144 Piccolo S.R., Withers M.R., Francis O.E., Bild A.H., Johnson W.E. Multiplatform single-sample estimates of transcriptional activation. *Proc Natl Acad Sci U S A* 2013; **110**: 17778-17783.

145 Tarazona S., Garcia-Alcalde F., Dopazo J., Ferrer A., Conesa A. Differential expression in RNA-seq: a matter of depth. *Genome Res* 2011; **21**: 2213-2223.

146 Sowalsky AG. *et al*. Whole transcriptome sequencing reveals extensive unspliced mRNA in metastatic castration-resistant prostate cancer. *Mol Cancer Res* 2015; **13**: 98-106.

147 Adiconis X. *et al*. Comparative analysis of RNA sequencing methods for degraded or low-input samples. *Nat Methods* 2013; **10**: 623-629.

148 Kim D. *et al*. TopHat2: accurate alignment of transcriptomes in the presence of insertions, deletions and gene fusions. *Genome Biol* 2013; **14**: R36.

149 Kent W.J. *et al*. The human genome browser at UCSC. *Genome Res* 2002; **12**: 996-1006.

150 Karolchik D. *et al*. The UCSC Table Browser data retrieval tool. *Nucleic Acids Res* 2004; **32**: D493-496.

151 Li H. *et al*. The Sequence Alignment/Map format and SAMtools. *Bioinformatics* 2009; **25**: 2078-2079.

152 Ramirez F. *et al*. deepTools2: a next generation web server for deep-sequencing data analysis. *Nucleic Acids Res* 2016; **44**: W160-W165.

153 Sims D., Sudbery I., Ilott N.E., Heger A., Ponting C.P. Sequencing depth and coverage: key considerations in genomic analyses. *Nat Rev Genet* 2014; **15**: 121-132.

154 Gruber A.R., Bernhart S.H., Lorenz R. The ViennaRNA web services. *Methods Mol Biol* 2015; **1269**: 307-326.

155 Zuker M., Stiegler P. Optimal computer folding of large RNA sequences using thermodynamics and auxiliary information. *Nucleic Acids Res* 1981; **9**: 133-148.

156 Liao Y., Smyth G.K., Shi W. featureCounts: an efficient general purpose program for assigning sequence reads to genomic features. *Bioinformatics* 2014; **30**: 923-930.

157 Robinson M.D., McCarthy D.J., Smyth G.K. edgeR: a Bioconductor package for differential expression analysis of digital gene expression data. *Bioinformatics* 2010; **26**: 139-140.

158 Robinson M.D., Oshlack A. A scaling normalization method for differential expression analysis of RNA-seq data. *Genome Biol* 2010; **11**: R25.

159 Law C.W., Chen Y., Shi W., Smyth G.K. voom: Precision weights unlock linear model analysis tools for RNA-seq read counts. *Genome Biol* 2014; **15**: R29.

160 Wang E.T. *et al*. Alternative isoform regulation in human tissue transcriptomes. *Nature* 2008; **456**: 470-476.

161 Durinck S., Spellman P.T., Birney E., Huber W. Mapping identifiers for the integration of genomic datasets with the R/Bioconductor package biomaRt. *Nat Protoc* 2009; **4**: 1184-1191.

162 Conway T. *et al*. Xenome--a tool for classifying reads from xenograft samples. *Bioinformatics* 2012; **28**: i172-178.

163 Subramanian A. *et al*. Gene set enrichment analysis: a knowledge-based approach for interpreting genome-wide expression profiles. *Proc Natl Acad Sci U S A* 2005; **102**: 15545-15550.

164 Huber W. *et al*. Orchestrating high-throughput genomic analysis with Bioconductor. *Nat Methods* 2015; **12**: 115-121.

165 Hartigan J.A., Wong M.A. Algorithm AS 136: A k-means clustering algorithm. *J R Stat Soc Ser C Appl Stat* 1979; **28**: 100-108.

166 Quackenbush J. Computational analysis of microarray data. *Nat Rev Genet* 2001; **2**: 418-427.

167 Weichselbaum R.R. *et al*. An interferon-related gene signature for DNA damage resistance is a predictive marker for chemotherapy and radiation for breast cancer. *Proc Natl Acad Sci* U S A 2008; **105**: 18490-18495.

168 Rich J.T. *et al*. A practical guide to understanding Kaplan-Meier curves. *Otolaryngol Head Neck Surg* 2010; **143**: 331-336.

169 Therneau T. *A package for survival analysis in S*, doi: 10.1093/survival/43.3.1 (2015).

170 Cox D.R. Regression models and life-tables. *Breakthroughs in Statistics*. 527-541 (Springer, 1992).

171 Efron B. The efficiency of Cox's likelihood function for censored data. *J Am Stat Assoc* 1977; **72**: 557-565.
